# Diversity of Solid Forms Promoted by Ball Milling: Characterization and Intrinsic Dissolution Studies of Pioglitazone Hydrochloride and Fluvastatin Sodium Drug–Drug Systems

**DOI:** 10.3390/ph16060781

**Published:** 2023-05-24

**Authors:** Marco Villeda-Villegas, José C. Páez-Franco, Guadalupe Coyote-Dotor, Alejandra Núñez-Pineda, Alejandro Dorazco-González, Inés Fuentes-Noriega, Kenneth Rubio-Carrasco, Helen P. Toledo Jaldín, David Morales-Morales, Juan Manuel Germán-Acacio

**Affiliations:** 1 Red de Apoyo a la Investigación, Coordinación de la Investigación Científica-UNAM, Instituto Nacional de Ciencias Médicas y Nutrición SZ, Ciudad de Mexico 14000, Mexico; villedamarco@gmail.com (M.V.-V.); paez@cic.unam.mx (J.C.P.-F.); ibtcoyotedg@gmail.com (G.C.-D.); 2 Centro Conjunto de Investigación en Química Sustentable CCIQS UAEM-UNAM Carretera Toluca-Atlacomulco km 14.5, Toluca 50200, Mexico; anp@unam.mx; 3 Instituto de Química, Universidad Nacional Autónoma de Mexico, Circuito Exterior, Ciudad Universitaria, Ciudad de Mexico 04510, Mexico; adg@unam.mx (A.D.-G.); damor@unam.mx (D.M.-M.); 4 Laboratorio de Biofarmacia, Departamento de Farmacia, Facultad de Química-UNAM, Ciudad de México 04510, Mexico; ifuentes@unam.mx (I.F.-N.); krc@quimica.unam.mx (K.R.-C.); 5 Technological Superior Studies Tianguistenco, Mechanical Engineering, Santiago Tianguistenco 52650, Mexico; helenpao@hotmail.com

**Keywords:** drug–drug coamorphous, drug–drug salt–cocrystal continuum, mechanochemical reactions, intrinsic dissolution experiments, fluvastatin sodium, pioglitazone hydrochloride, XPS experiments

## Abstract

Coamorphous salt in a 1:1 ratio prepared by ball milling from Fluvastatin sodium (FLV) and Pioglitazone hydrochloride (PGZ·HCl) can be selectively formed by neat grinding (NG). Furthermore, the salt–cocrystal continuum was preferably formed by employing liquid-assisted grinding (LAG) using ethanol (EtOH). Attempts to prepare the coamorphous salt starting from the salt–cocrystal continuum by NG were unsuccessful. Interestingly, through ball milling by NG or LAG, a great diversity of solid forms (PGZ·HCl-FLV 1:1) could be accessed: NG and hexane (coamorphous); ethyl acetate (physical mixture); EtOH (salt–cocrystal continuum); and water (which presents two T_g_, indicating immiscibility of the components). An exploration was performed at different drug-to-drug ratios by NG. By differential scanning calorimetry (DSC), the presence of two endothermic events was observed in this screening: incongruous melting point (solidus) and excess of one of the components (liquidus), except in the 1:1 solid form. From these results, eutectic behavior was observed. Through the construction of a binary phase diagram, it was determined that the 1:1 molar ratio gives rise to the formation of the most stable coamorphous composition. Dissolution profile studies of these solid forms were carried out, specifically on pure FLV and the solid forms of PGZ⋅HCl-FLV (1:2; 1:4; and 1:6), together with the coamorphous 1:1 salt. By itself, pure FLV presented the highest K_int_ (13.6270 ± 0.8127 mg/cm^2^⋅min). On the other hand, the coamorphous 1:1 showed a very low K_int_ (0.0220 ± 0.0014 mg/cm^2^·min), indicating very fast recrystallization by the FLV, which avoids observing a sudden release of this drug in the solution. This same behavior was observed in the eutectic composition 1:2. In the other solid forms, the value of K_int_ increases along with the %w of FLV. From the mechanochemical point of view, ball milling by NG or LAG became an important synthetic tool since it allows obtaining a great variety of solid forms to explore the solid-state reactivity of the drug–drug solid-form PGZ HCl-FLV.

## 1. Introduction

**Scheme 1:**
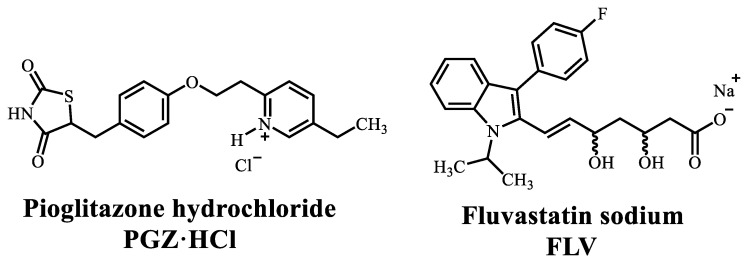
Molecular structures of PGZ·HCl and FLV.

Fluvastatin sodium (FLV) belongs to the family of statins, drugs specialized in lowering cholesterol [1,2,3] (Scheme 1). Statins inhibit HMG-CoA reductase, implying a reduction in cholesterol synthesis in the blood [1,3]. Likewise, pioglitazone hydrochloride (PGZ·HCl) is part of the class of thiazolidinediones (TZDs) and rosiglitazone (Scheme 1). TZDs are insulin-sensitizing drugs, also known as PPARγ inhibitors [4]. These molecules can reduce plasma glucose and insulin levels, improving the lipid disorders that patients with type 2 diabetes mellitus may present [5]. In this sense, combining statins with TZD may benefit treating dyslipidemic diabetic individuals [4,5]. FLV was approved by the Food and Drug Administration (FDA) in 1998 and was patented in 1994 as Lescol®, which expired in 2011 [2]. Lescol® was presented to the market as immediate release (capsule) or sustained release (8 h 80 mg matrix tablet, Lescol® XL) [6]. FLV exhibits various crystalline structures, which impacts this drug’s solubility performance and dissolution profiles. Many of these different crystalline structures have been patented. Still, the solid-state and dissolution profile characterization has been vague and poorly informed [2]. 

In this sense, Cardoso et al. studied intrinsic dissolution rates (IDR) of various crystalline forms of FLV. They evaluated different crystalline forms: FLV raw material (RM), FLV USP (reference substance), and FLV crystallized in multiple solvents, ethanol, and acetonitrile. In general, FLV (RM) presented the best dissolution profile, and the authors comment that these IDR values may depend on the polymorphism and morphology of FLV [2]. On the other hand, there is a discrepancy in the literature in determining whether FLV has solubility problems. Some authors mention that it belongs to class II [7] according to the biopharmaceutical classification system (BCS) (low solubility and high permeability). In this way, Sharma et al. mention that FLV is class II. For this, they prepared solid dispersions (SDs) by kneading, combining FLV with polyethylene glycol 6000 (PEG 6000) and polyvinyl pyrrolidone K-30 (PVP K30), to improve the solubility of the drug in question [7]. The findings showed improvements in the in vitro experiments (solubility and dissolution profiles) compared to the pure drug. Before this article, Sharma published the preparation of SDs (FLV in the presence of PEG 6000 and PVP K30) but prepared differently [8]. They mention that FLV is practically insoluble in water; additionally, they do not specify what happened to the solubility of the FLV-SDs systems. El-Helw has also published the preparation of nanostructured lipid carriers containing FLV (FLV-NLC) to increase the aqueous solubility of the statin [9]. This work aimed to prepare these FLV-NLCs to improve the bioavailability of FLV. The drug-release experiments indicated it to be a sustained-release system that considerably enhanced bioavailability.

On the other hand, Bikiaris et al. mention that FLV is highly soluble in water (>50 g/L) [10]. The authors prepared different SDs (Eudragit RS 100, Chitosan, and PVP) with FLV. They only performed drug release profiles for FLV-PVP since they described that PVP dissolves rapidly in water. The highest release rate was observed for FLV-PVP containing 10–20 %w of FLV. As the FLV content increased in the SDs, the release rate decreased. Although they do not specify whether they conducted release rate experiments with the other SDs, the authors mention that depending on the drug carrier, it produces completely different dissolution profiles for each SD, suggesting that each matrix follows a different drug release mechanism.

FLV is highly hygroscopic, making it unstable as it exhibits various conformational changes. These conformational changes are due to modifications in intermolecular interactions, which can alter its solubility in water and its bioavailability [11].

In this sense, there are disagreements about whether FLV should be considered a class II drug or a highly water-soluble compound. In this way, we want to follow the premise that Cardoso mentioned, depending on the crystalline form of FLV, which can affect its solubility and dissolution profiles. This paper describes the mechanochemical preparation by ball milling the solid-form drug–drug PGZ·HCl-FLV in different stoichiometric ratios. Due to the great synthetic versatility that mechanochemistry has shown in recent years, these solid forms will be prepared using neat grinding (NG) and liquid-assisted grinding (LAG) [12,13]. We chose PGZ⋅HCl as a drug coformer due to the potential therapeutic benefit in combinatorial therapy presented by TZD statins in diabetic patients with dyslipidemia [4,5]. It should be remembered that coamorphous materials belong to the SD family and are based on combining two or more low-molecular-weight components to form a homogeneous amorphous monophasic solid form [14,15,16]. In this sense, IDR studies will be carried out on the solid forms obtained through mechanochemical exploration to determine if these properties were modified. Furthermore, IDR studies explain the relationship between the dissolution rate and the solid form in which the drug is found [17]. In this way, these studies are vital in formulating pharmaceutical dosage forms. 

## 2. Materials and Methods

### 2.1. Materials

All the pharmaceutical reagents were purchased from Tokyo Chemical Industry™ (PGZ·HCl: P1901) or Merck-Supelco México™ (FLV: PHR1620) and were used as received. The solvents were purchased from Tecsiquim™ and were used as received.

### 2.2. Methods 

#### 2.2.1. NG or LAG Solvent Screening (Stoichiometry Ratio 1:1)

NG or LAG solvent screening was performed to prepare the solid forms using a Planetary Micro Mill Pulverisette^TM^ 7 Fritsch device. [12] PGZ·HCl (97.46 mg, 0.248 mmol) and FLV (107.49 mg, 0.248 mmol) were used in a 1:1 stoichiometric ratio. For every LAG experiment, 100 μL of solvent was added. The solvents used were hexane, ethyl acetate (AcOEt), ethanol (EtOH), and water [12,13]. Stainless steel bowls of 20 mL containing 10 stainless steel balls (10 mm diameter) were used. The NG or LAG experiments were conducted at 600 rpm for 30 min.

#### 2.2.2. Evaluation of the Formation of the Multicomponent Salt PGZ-FLV (EtOH, Stoichiometric Ratio 1:1) at Different Grinding Times

Mechanochemical studies of PGZ-FLV (LAG with EtOH) were carried out by lengthening the milling times (Planetary Micro Mill Pulverisette^TM^ 7 Fritsch device) and applying heat treating (H.T.) at 140 °C. PGZ·HCl (194.92 mg, 0.496 mmol) and FLV (214.98 mg, 0.496 mmol) were used. At the beginning, 100 μL EtOH was added. Stainless steel bowls of 20 mL containing 10 stainless steel balls (10 mm diameter) were used. The experiments were carried out at 600 rpm. A sample was periodically withdrawn to be analyzed by XRPD at 30, 60, 90, and 120 min. To the sample at 120 min was applied H.T. (120 min + H.T.). H.T. was carried out using an OMH60 Heratherm Thermo Scientific^®^ mechanical convection oven. An initial temperature of 50 °C was started, with a heating rate of 10 °C/min, and the maximum temperature reached 140 °C (for 1 h). Once the powders have been removed from the oven, they are ground while hot in an agate mortar for 30 min (120 min + H.T.). 

#### 2.2.3. Evaluation of the Amorphization Ability of the PGZ·HCl

Starting with 250 mg of PGZ·HCl, it was ball-milled in a Planetary Micro Mill Pulverisette^TM^ 7 Fritsch device for 150 min under NG. Stainless steel bowls of 20 mL containing 10 stainless steel balls (10 mm diameter) were used. The experiments were carried out at 600 rpm. A sample was periodically withdrawn to be analyzed at 30, 60, 90, and 120 min. It should be noted that approximately 40 mg of the sample was periodically removed (30, 60, 90, 120, and 150 min) to be characterized by XRPD and DSC-TGA.

#### 2.2.4. Evaluation of the Formation of the PGZ·HCl-FLV Solid Forms (1:2; 1:4; 1:6; 1:8 and 1:10)

Table 2 shows the amounts in mg of each drug used to prepare the corresponding solid forms. For each solid form, NG was used for 30 min. A Planetary Micro Mill Pulverisette^TM^ 7 Fritsch device was used. Stainless steel bowls of 20 mL containing 10 stainless steel balls (10 mm diameter) were used. The experiments were carried out at 600 rpm.

#### 2.2.5. Evaluation of the Formation of the PGZ·HCl-FLV Solid Forms (2:1; 4:1; 6:1; 8:1 and 10:1)

Table 4 shows the amounts in mg of each drug used to prepare the corresponding solid forms. For each solid form, NG was used for 30 min. A Planetary Micro Mill Pulverisette^TM^ 7 Fritsch device was used. Stainless steel bowls of 20 mL containing 10 stainless steel balls (10 mm diameter) were used. The experiments were carried out at 600 rpm.

#### 2.2.6. Thermal Analysis

Different types of equipment were used interchangeably to carry out the DSC and TGA experiments. A simultaneous thermal analyzer Netzsch STA 449 F3 Jupiter was used. A DSC Q100 V9.9 Build 303 (TA instruments) was used. Additionally, a TGA Q5000 V3.17 Build 265 (TA instruments) equipment was employed. The samples were placed (2–4 mg) in sealed non-hermetic aluminum pans and were scanned at a heating rate of 10 °C/min from 30−400 °C under a dry nitrogen atmosphere. The calculated glass temperature (T_g_) values of synthesized solid forms were predicted employing the Gordon–Taylor equation [18].

$$T_{gmix}=\frac{w_{1}T_{g1}+w_{2}T_{g2}K}{w_{1}+w_{2}K}\;K=\frac{T_{g1}\cdot \rho_{1}}{T_{g2}\cdot \rho_{2}}$$

T_g1_ and T_g2_ are glass transition of components 1 (FLV: 69.5 °C) [10] and 2 (PGZ·HCl: 64.4 °C) [19], w_1_ and w_2_ are weight fractions of the components, and T_gmix_ is the glass transition of the coamorphous mixture. Density values were obtained from the literature: FLV (1.20 g/cm^3^) [20] and PGZ·HCl (1.26 g/cm^3^) [21]. 

The crystallinity of the participating drugs within the coamorphous mixture was determined using the Rawlinson equation [22].

$$\%Crystallinity=\frac{\Delta H_{m\;coamorphous}}{\Delta H_{m\;drug}\cdot w}\cdot 100$$

where ΔH_m coamorphous_ is the enthalpy of the coamorphous mixture (J/g), ΔH_m drug_ is the enthalpy of the pure drug (J/g), and w is the weight fraction of the drug in the coamorphous mixture.

#### 2.2.7. X-ray Powder Diffraction (XRPD)

XRPD experiments were carried out in a Bruker D8 Advance diffractometer with Bragg–Bretano geometry, Cu Kα radiation (1.54060 Å), and a Linxeye detector. Each sample was measured by a continuous scan between 5 and 60° in 2θ, with a step time of 151.19°/min and a step size of 0.0198°. 

#### 2.2.8. Nuclear Magnetic Resonance

CP MAS solid-state NMR (SSNMR) spectra were recorded in a Bruker Avance II 300 spectrometer (operating at ^1^H 300 MHz, ^13^C 75 MHz, and ^15^N 30 MHz). SSNMR measurements were carried out on a 4 mm double rotor resonance CP-MAS probe at a 5–6 kHz spinning rate with a cross-polarization contact time of 2 ms and delay of 5 s. In addition, HMBC and HSQC experiments were carried out in a Bruker Avance III 500 (operating at ^1^H 500 MHz, ^13^C 125 MHz, and ^15^N de 50 MHz). Solution NMR measurements were carried out on a 4 mm broadband probe with two channels; the heteronuclear channel can be tuned ^31^P (202 MHz) until ^107^Ag (27 MHz), with Z-axis gradients. The HMBC and HSQC ^15^N experiments used NH_3(l)_ δ = 0 ppm as the internal reference and glycine (δ = 38 ppm) as the secondary standard. 

#### 2.2.9. FT-IR

An ALPHA II Platinum ATR Bruker spectrometer with a device for solid-state samples was used. In total, 64 scans were performed on each sample.

#### 2.2.10. Intrinsic Dissolution Studies

The intrinsic dissolution constants (K_int_) were determined according to the conditions established in the Pharmacopoeia of the United Mexican States (PUMS) 13th edition [23]. The experiments used tablets prepared with a hydraulic press with a pressing force of 250 kg/cm^2^. Dissolution rates were determined using Wood’s apparatus according to the pharmacopoeial technique (PUMS 13th edition). Dissolution profiles were performed using distilled water (pH = 6.50) as the established dissolution medium for Fluvastatin sodium salt (PUMS 13th edition). Distilled water was obtained from an Elix-3 Essential system at 15 mΩ·cm resistivity. The experiments were carried out in triplicate at 37 °C under constant stirring (100 rpm) in a continuous volume of 900 mL. The profiles were quantified using an Agilent 1260 series Infinity II HPLC equipment, with a high-performance autosampler (G1367E) under the following chromatographic conditions: mobile phase MeOH and 0.01 M H_3_PO_4_, pH = 2 (75:25), a flow of 1 mL/min, using a Zorbax Eclipse XDB-C18 column, 4.6 × 150 mm, a particle size of 5 μm, a diode array UV-vis detector, and samples were measured at a wavelength of 238 nm.

#### 2.2.11. Eutectic Binary Mixture Screening by DSC Data

The binary phase diagram was constructed from the thermograms of the different molar ratios prepared by NG. In the case of molar proportions 2:1; 4:1; 6:1; 8:1, and 10:1, the appearance of two endothermic events (solidus and liquidus points) was clear [24]. It should be noted that these samples were run at a heating rate of 10 °C/min. However, in the case of molar ratios 1:1; 1:2; 1:4; 1:6; 1:8, and 1:10, this differentiation of the solidus and liquidus points was not so evident, and they were run at 2 °C/min since we have previously seen that, to improve the visualization precision in some thermal events, it is necessary to change the rates heating [25]. These samples were run on a Netzsch STA 449 F3 Jupiter simultaneous thermal analyzer. In ratios 1:8 and 1:10, it was impossible to differentiate the two events of incongruent fusion and excess of PGZ⋅HCl. In addition, the Tammann triangle plot was constructed using the values of ΔH_m_ solidus run at 10 °C/min [26]. The ΔH_m_ solidus of outcomes 1:8 and 1:10 were not considered in the plot because they could not be determined precisely on the thermogram.

#### 2.2.12. Scanning Electron Microscopy Studies (SEM)

SEM evaluated the morphology of each solid form on a JEOL (JSM-6610) microscope. For sample preparation, the specimen was dried and fixed on a stub with carbon double-stick tape and then coated with gold for 90 s under vacuum using a Denton IV sputtering chamber.

#### 2.2.13. X-ray Photoelectron Spectroscopy (XPS)

XPS analyses were carried out in an ultra-high vacuum (UHV) system scanning XPS microprobe PHI 5000 Versa Probe II, with an Al K_α_ X-ray source (hν = 1486.6 eV) monochromatic with a 100 µm beam diameter and a multi-channel detector (MCD) analyzer. The XPS spectra were obtained at 45° to the normal surface with constant analyzing energy (CAE) E_0_ = 117.40 and 11.75 eV survey surface and a high-resolution narrow scan. The peak positions were referenced to the background Ag 3d_5/2_ photopeak at 368.20 eV, with an FWHM of 0.56 eV, and C 1s hydrocarbon groups at 285.00 eV, Au 4f_7/2_ at 84.00 eV central peak core level position. The XPS spectrum was fitted with the MultiPak PHI software [27] and a spectral data processor SDP v 4.1 [28].

## 3. Results

### 3.1. NG and LAG Solvent Screening (Stoichiometry 1:1)

**Figure 1:**
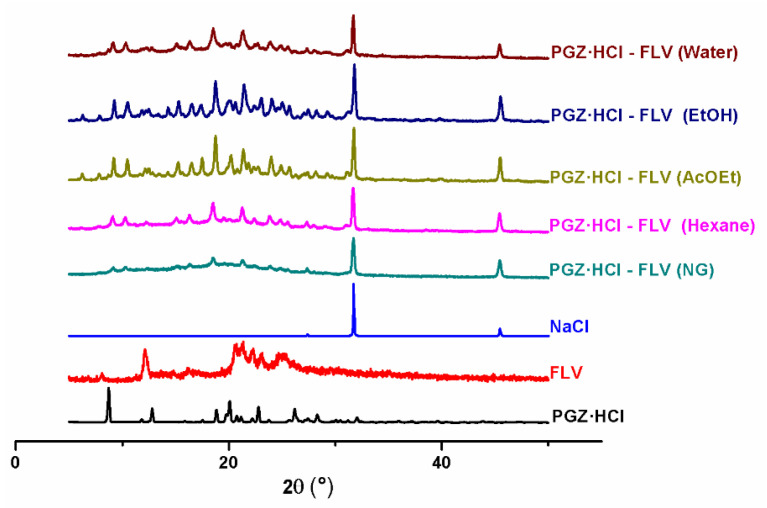
Diffractograms of the solid phases PGZ⋅HCl-FLV (1:1) prepared by NG or LAG solvent screening.

The first studies for preparing this solid binary form PGZ·HCl-FLV were carried out in the stoichiometric ratio (1:1). This molar ratio was used as a reaction model to explore mechanochemical reactions by NG or LAG. LAG employed various solvents (hexane, AcOEt, EtOH, and water) to see their effect on forming the solid form. Initially, the results were analyzed by XRPD (Figure 1). At first glance, the solid binary forms obtained by NG or LAG present a different diffractogram than pure drugs. In fact, in the case of NG, it was the one that showed a considerable amorphous contribution; however, incipient reflections due to PGZ·HCl are noted. In addition, the presence of NaCl, an expected byproduct of the grinding reaction, is observed. In the case of the diffractogram of the solid form (EtOH), the corresponding result is 120 + H.T. (Section 2.2.2).

**Figure 2:**
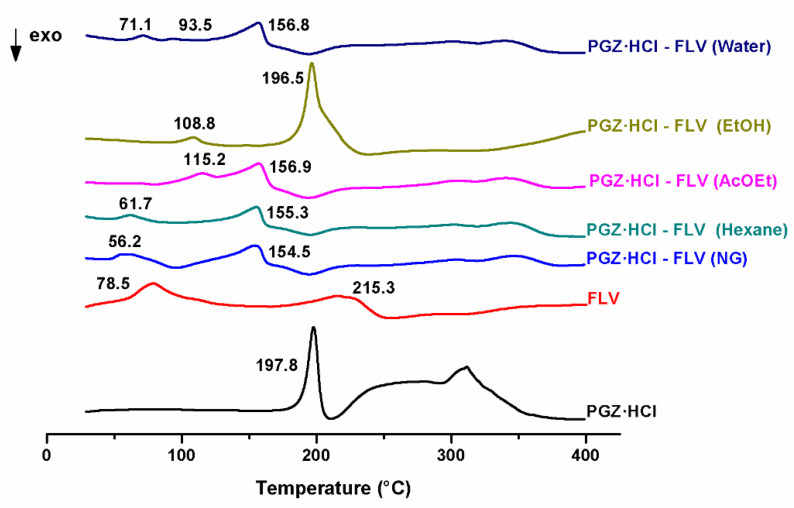
DSC thermograms of the solid phases PGZ⋅HCl-FLV (1:1) prepared by NG or LAG solvent screening.

Additionally, these solid phases were also evaluated by DSC (Figure 2). In the case of the thermogram of the solid form (EtOH), the corresponding result is 120 + H.T. (Section 2.2.2). The Supplementary Material (Figure S1) presents the individual DSC-TGA thermograms of the solid binary forms and pure drugs. The thermodynamic data of all the outcomes are concentrated in Table 1.

In the case of FLV, a first thermal event is observed at 78.5 °C and corresponds to the release of a water molecule of hydration, concerning the data provided by the TGA. In contrast, 215.3 °C corresponds to the melting temperature (T_m_) [2]. PGZ·HCl shows a DSC thermogram with a single event corresponding to the melting temperature of 197.8 °C [29]. The DSC thermogram of the solid-form PGZ·HCl-FLV (NG) presents three thermal events: (T_g_: 56.2 °C), crystallization temperature (T_c_: 95.8 °C, exo), and T_m_: 154.5 °C. The presence of a single T_g_ value suggests the miscibility of the components to form an amorphous single-phase (coamorphous) mixture since if two T_g_ values were observed, it would indicate that the mixture of the constituents would be separated into two phases [30]. The T_g_ event presents an enthalpy relaxation endotherm [18]. Typically, a T_g_ signal does not exhibit an endothermic contribution. When it has one, it is due to an enthalpic relaxation (ΔH) due to the aging or relaxation of the amorphous sample [18]. This endothermic enthalpy relaxation effect increases as the solid form ages or relaxes. The degree of relaxation that one of the components in the mixture can present is a function of the enthalpy change. This should be considered an equilibrium of going from a glassy state to a supercooled liquid [31]. In this case, the solid-form PGZ·HCl-FLV 1:1 prepared by NG presented an enthalpy relaxation contribution of 11.66 J/g (Figure S1). The crystallization event (ΔH_c_) shows an enthalpy value of 22.1 J/g. This T_c_ event is due to PGZ·HCl, as discussed in Section 3.3. PGZ·HCl is reluctant to being amorphized by ball milling. The crystallinity percentages of each drug within the coamorphous mixture were determined using the Rawlinson equation (Section 2.2.6) (Table 1). PGZ·HCl (65.85%) and FLV (75.13%) values were found. Therefore, it is suggested that once the coamorphous (single-phase amorphous) mixture is formed, both drugs cannot stabilize each other in an amorphous form, undergoing recrystallization in both cases. It should be noted that even when this coamorphous mixture establishes strong interactions (see Section 3.2, Figure 8), it does not serve as a stabilizing factor to avoid the enthalpy effect of relaxation. This recrystallization process of one of the components within coamorphous systems has already been seen previously in tadalafil-repaglinide [32].

The solid phase PGZ·HCl-FLV (hexane) presents only two events–T_g_: 61.7 °C and T_m_: 155.3 °C. The calculation of the percentage crystallinity of the components was FLV (89.55%) and PGZ·HCl (78.49%). Comparing these results with those obtained for the solid form prepared by NG, the latter presents a greater amorphic contribution. Based on this, the formation of the coamorphous 1:1 mixture can be further favored using NG. The binary phase PGZ HCl-FLV (AcOEt) from the DSC is a physical mixture since it presents two fusion events (115.2 and 156.9 °C). The solid phase PGZ·HCl-FLV (EtOH) exhibits an enthalpy relaxation endotherm (108.8 °C), and the melting temperature is observed at T_m_: 196.5 °C. Finally, PGZ·HCl-FLV (water) presents two values of T_g_ (71.1 and 93.5 °C). After these events, the T_m_ event is observed at 156.6 °C. The presence of two T_g_ indicates that the components are physically separated [30]. 

The T_g_ value for NG was calculated (67.07 °C) using the Gordon–Taylor equation (Section 2.2.6). It should be considered that this equation does not consider the interactions that the components may present [33,34]. In this way, it was argued that when the calculated value is below the experimental value, it is due to an establishment of interactions not contemplated. In our case, the opposite happens; the estimated value is higher. In this regard, it can be mentioned that with sucrose inhibiting its crystallization, adding various polymers can prevent this due to the additives establishing interactions with the carbohydrate [35]. The authors point out that molecular mobility should not be viewed as the only factor controlling the inhibition of sucrose crystallization. Other thermodynamic and geometric factors must be considered within the nucleation processes that give rise to recrystallization [35]. Thus, apart from the fact that the Gordon–Taylor equation does not consider the interaction between the components, other unforeseen factors must exert an influence, hence this deviation. The relaxation enthalpy effects are not considered in this equation which may affect the fact that the experimental and calculated values correlate poorly. 

With these results, it can be said that the solid form obtained from NG is coamorphous.

On the other hand, employing LAG using EtOH favored the formation of the salt–cocrystal continuum. Later in this section, we will describe how this material was determined to have formed using X-ray photoelectron spectroscopy (XPS). Note the great difference in ΔH_m_ values among the coamorphous form obtained by NG and the salt–cocrystal continuum obtained by LAG using EtOH (Table 1). This solid form presents a ΔH_m_ value 5.77 higher than the coamorphous mixture obtained by NG. 

We evaluated the stability of the salt–cocrystal continuum at different milling times and applied H.T. to prove if this may favor coamorphous formation (Figure S2). Unfortunately, we could not observe any change from the diffractograms once the salt–cocrystal continuum was formed, increasing the milling times and applying H.T. This does not favor the formation of the coamorphous mixture.

Rades et al. have recently published the obtaining of coamorphous systems by ball milling (carbamazepine (CBZ)) starting from the cocrystal CBZ-TAR 1:1 (TAR: tartaric acid) [36]. Notably, other cocrystals were explored in an attempt to amorphize them by ball milling, but the results were unsuccessful. They observed that in physical mixtures (CBZ−MEA, CBZ−TAR, and CBZ−SAC, MEA: maleic acid and SAC: saccharin), it was possible to access the formation of the corresponding coamorphous mixture by ball milling, although they showed short stability times and tended to recrystallize. Likewise, they also found that these CBZ-coformer systems, starting from a physical mixture, are more feasible to obtain the coamorphous mixture by ball milling than to try to prepare the coamorphous mixture from the cocrystal. This is mentioned by the unsuccessful attempt to obtain the coamorphous mixture from the salt–cocrystal continuum by extending the milling times (Figure S2). This may be because the ΔH_m_ value is so high (Table 1) compared to pure drugs. As a result, stronger intermolecular interactions are established within the crystal lattice of the salt–cocrystal continuum. The energy provided by ball milling is not enough to break them down and amorphize the components to give rise to the coamorphous mixture. Something interesting to mention is that the LAG screening solvent and NG promoted different binary solid forms: NG and hexane (coamorphous); AcOEt (physical mixture); EtOH (salt–cocrystal continuum) and water (which presents two T_g_, indicating immiscibility of the components). It has already been mentioned that LAG confers mobility to the components involved, imparting additional degrees of freedom (orientational and conformational) to the molecules that affect the result of the reaction, which otherwise cannot be accessed by NG [37]. This diversity in obtaining different solid forms prepared by LAG or NG can allow studying the formation mechanisms of cocrystals since at least three mechanisms have been proposed: molecular diffusion, eutectic formation, and mediation by an amorphous phase [12]. This demonstrates the potential of mechanochemistry to obtain solid forms that can be considered intermediates to propose reaction mechanisms. Studies show a cocrystal can be formed via an amorphous state [38,39].

**Figure 3:**
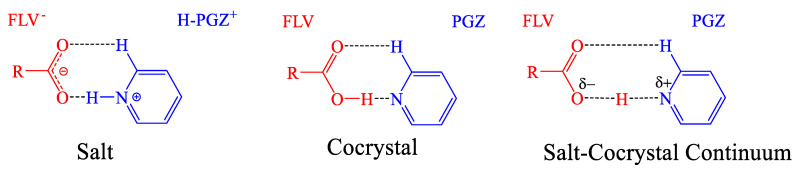
Molecular recognition −COO^−^⋅⋅⋅^+^H-N_pyr_ (salt), −COOH⋅⋅⋅N_pyr_ (cocrystal), or −COO^δ−^⋅⋅⋅H⋅⋅⋅^δ+^N_pyr_ (salt-cocrystal continuum).

As mentioned, this section will discuss why the solid form obtained by LAG using EtOH is a salt–cocrystal continuum, mainly using SSNMR and XPS. In this case, the outcome 120 + H.T. was used. The salt–cocrystal continuum form is when a proton is between the acid and the base (quasi-proton state, equidistant between the two) [40]. Using these analytical techniques, we can differentiate what situation this solid form is in depending on the synthon it has formed (Figure 3).

**Figure 4:**
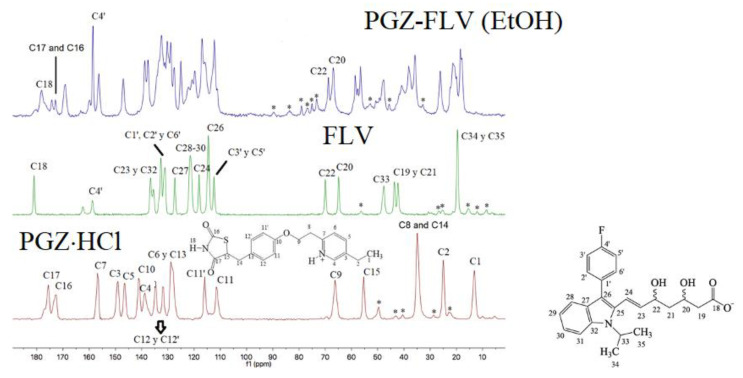
^13^C SSNMR spectra of PGZ∙HCl, FLV, and the solid-form PGZ-FLV (EtOH) (stoichiometry 1:1). * Spinning sidebands.

**Figure 5:**
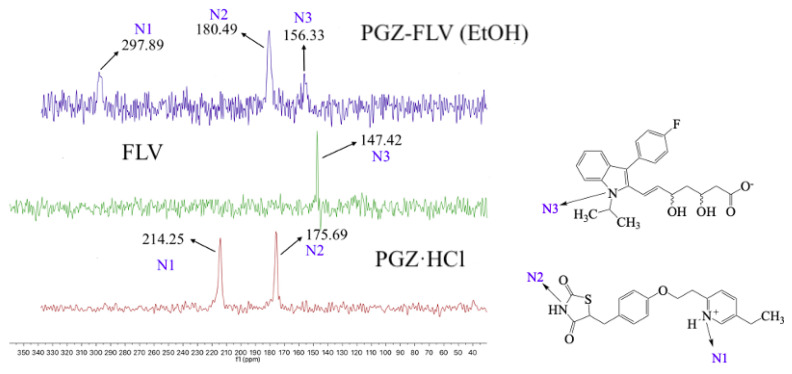
^15^N SSNMR spectra of PGZ∙HCl, FLV, and the solid-form PGZ·HCl-FLV (EtOH).

This solid-form PGZ-FLV (EtOH) was analyzed by ^13^C and ^15^N SSNMR (Figure 4 and Figure 5). The assignment of δ in ^13^C SSNMR of FLV was made based on what has already been reported [2]. In ^13^C, SSNMR of PGZ∙HCl was determined by comparing the already reported NMR in solution in d_6_-DMSO [41]. As can be seen in Figure 4, the ^13^C SSNMR spectra of PGZ∙HCl, FLV and the solid-form PGZ-FLV (EtOH) are found. Notably, in the case of the FLV spectrum, the signals at 158.1 and 162.5 ppm are due to a splitting of C4’ due to a scalar spin–spin C4’-F coupling of 267 Hz [2]. On the other hand, in the solid-form PGZ-FLV (EtOH) spectrum, only the most important ^13^C atoms that participate in relevant molecular interactions are indicated. For example, in the first instance, a Δδ = −2.54 ppm (δ 178.5 _solid-form PGZ-FLV (EtOH)_ − δ 181.04 _FLV_) can be observed in the C18 atom. This indicates that C18 underwent an upfield shift experiencing protection because the -COO^−^ group of FLV established a new intermolecular interaction once it was formed, suggesting the formation of a new synthon. Subsequently, C17 and C16 in the solid-form PGZ-FLV (EtOH) a ΔδC17 = −1.3 ppm (δ 174.4 _solid-form PGZ-FLV (EtOH)_ − δ(75.7 _PGZ∙HCl_) and ΔδC16 = 0.8 ppm (δ (73.2 _solid-form PGZ-FLV (EtOH)_) − δ 172.4 _PGZ∙HCl_) are observed. C17 and C16 correspond to the carbonyl groups -C=O of the TZD ring of PGZ·HCl.

Additionally, C4’ of the FLV once the solid-form PGZ-FLV (EtOH) is formed, a ΔδC4’ = 0 ppm (δ 158.8 _solid-form PGZ-FLV (EtOH)_ − δ 158.8 _PGZ∙HCl_) is observed; however, a decrease in the scalar spin–spin coupling is detected C4’-F of 112.5 Hz. Subsequently, shifts are perceived in carbons C22 and C20 (−C-OH) of the FLV in the solid-form PGZ-FLV (EtOH), a ΔδC22 = −0.8 ppm (δ 69.2 _solid-form PGZ-FLV (EtOH)_ − δ 70.0 _PGZ∙HCl_) and ΔδC20 = 2.5 ppm (δ 67.4 _solid-form PGZ-FLV (EtOH)_ − δ 64.9 _PGZ∙HCl)_). 

In addition, HSQC and HMBC spectroscopy experiments were performed. Figures S3 and S4 show the HMBC and HSQC ^1^H-^15^N NMR spectra of PGZ∙HCl in d_6_-DMSO. These experiments determined values (in solution) of the ^15^N nuclei in PGZ·HCl [41]. These δ determinations served to assign the δ values of ^15^N SSNMR.

Concerning ^15^N SSNMR spectra for PGZ·HCl, FLV, and the solid-form PGZ-FLV (EtOH), they are presented in Figure 5. The N1 (PGZ⋅HCl) signal, compared to N1 of the solid binary form EtOH, shows a Δδ = 83.64 ppm (δ 297.89 _solid-form PGZ-FLV (EtOH)_ − δ 214.25 _PGZ∙HCl_), indicating that a new interaction was formed [42,43,44,45,46,47,48,49,50,51]. In the first instance, the chemical shift value of N1 in the solid-form PGZ-FLV (EtOH) indicates that it is a deprotonated pyridine moiety [43,48]. This suggests that the carboxylate abstracted the proton. In this way, molecular recognition of the type −COOH⋅⋅⋅N_pyr_ (cocrystal) or −COO^δ−^⋅⋅⋅H⋅⋅⋅^δ+^N_pyr_ (salt–cocrystal continuum) was established (Figure 3). Unfortunately, several attempts to crystallize this solid binary form (EtOH) failed to prove that proton transfer existed. However, XPS can be an important tool for differentiating whether a multicomponent solid form is a salt, cocrystal, or salt–cocrystal continuum [40,43,52]. According to the pK_a_ rule of three (ΔpKa = pKa_[base]_ − pKa_[acid]_), [53] considering the pK_a_ values of the initial components PGZ⋅HCl (4.02_strongest acidic_ and 5.65_strongest basic_) [54] and FLV (4.5), ref.[55] ΔpK_a_ < 0 indicates a neutral binary adduct (cocrystal). Although the found value of ΔpKa was 0.48, it should not be considered a salt since it must satisfy ΔpKa > 3, while if the difference is less than 1, a cocrystal is obtained. It should be remembered that this is an empiric rule. The N 1s XPS spectra of PGZ⋅HCl, FLV, and the solid binary form (EtOH) are shown in Figure S5. They exhibit peaks corresponding to the photoemissions of C=NH^+^ (C7=N1) and C-NH (C4-N1 and C16/17-N2) for PGZ⋅HCl, C-N (C25/32/33-N3) for FLV, and C=N (C7=N1), C-NH (C4-N1 and C16/17-N2), and C-N (C25/32/33-N3) for PGZ-FLV (EtOH). The photoemission corresponding to C=NH^+^ (398.65 eV) for PGZ⋅HCl experienced a shift of −0.20 eV in agreement with that observed in PGZ-FLV (EtOH) (398.45 eV). The chemical shift was calculated according to the formula (ΔBE = BE_binary adduct_ − BE_initial component_; binding energy (BE)). This change in chemical shift to lower binding energy indicates the disappearance of localized positive charge, implying that N1 is being deprotonated [52,56]. Recently, Tothadi et al., using XPS N 1s BE combined with single-crystal X-ray diffraction, were able to unequivocally assign in which situation different binary adducts are found according to the different synthons described in Figure 3 [40]. It is concluded in a general way that, according to N 1s BE, the cocrystals containing the moieties above, the values will be found in the interval of 398.7–398.9 eV, and the case of salts, in 400.1–401.1 eV. By comparing the reported binary solid forms, the values of ΔBE can be taken as a reference to determine where our results fit. According to this article, the ΔBE to decide whether it is a cocrystal–salt continuum is 0.4–0.6 eV. However, in the case of the binary system 3,5-Dinitrobenzoic Acid-4-Cyanopyridine with a ΔBE of 0.0 eV, they found that it was a salt–cocrystal continuum. When the salt has been formed, a ΔBE of 0.8–2.7 eV is observed. In the case of cocrystals, a ΔBE of 0.0 eV was reported. From our results, we can propose that the salt–cocrystal continuum was formed (Figure 3); however, we cannot fully state this without single-crystal X-ray diffraction results. However, what can be confirmed is that the H-PGZ^+^ pyridinium fragment is deprotonated once it interacts with FLV, according to SSNMR and XPS. In the other peaks corresponding to the photoemissions of C-NH (400.20 eV) for PGZ⋅HCl and C-N (399.85 eV) for FLV, no chemical shift change is observed according to the spectrum of PGZ-FLV (EtOH) (Figure S5).

Subsequently, in the ^15^N SSNMR spectra, N2 shows a Δδ = 4.80 ppm and N3 a Δδ = 8.91 ppm. The very small Δδ observed in N2 and N3 reflect that these atoms are participating in intermolecular interactions of moderate strength. Thus, it is observed that by utilizing NG or LAG, it is possible to selectively access the formation of the coamorphous mixture (NG, stoichiometry 1:1) or, on the other hand, the salt–cocrystal continuum. 

In this way, employing NG, ball milling will be carried out to form the coamorphous mixture of the solid-form PGZ·HCl-FLV in different stoichiometric ratios.

The salt–cocrystal continuum sample was also analyzed by FT-IR, and the full and expanded spectra (2000–1200 cm^−1^) are presented in Figure S6. The table with the frequency values is also shown in Figure S6. The vibrational modes −C=O_PGZ_ (a and b; 1744 and 1690 cm^−1^) [57] and −C=O_FLV_ (1576 cm^−1^) [2] of the pure drugs were evaluated. Shifts at high frequencies were observed in all vibrational modes analyzed: −C=O_PGZ_ (a and b; Δν = 18 and 21) and −C=O_FLV_ (Δν = 39). According to what has been published, it is described that the carboxylate fragment in the Fluvastatin sodium salt was recorded as 1576 and 1573 cm^−1^ [10,11]. In the specific case of −C=O_FLV_, the shift at high frequencies is consistent with that observed in XPS, where the pyridinium fragment is deprotonating in the presence of the carboxylate.

### 3.2. Evaluation of the Formation of the PGZ·HCl-FLV Solid Forms (1:2; 1:4; 1:6; 1:8 and 1:10)

**Figure 6:**
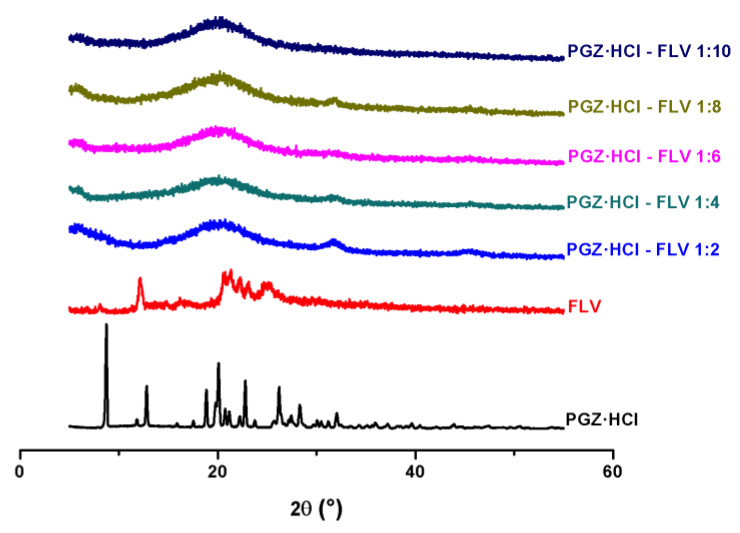
XRPD diffractograms of the solid forms of PGZ·HCl-FLV at different stoichiometric ratios (1:2; 1:4; 1:6; 1:8, and 1:10).

Different molar ratios (1:2; 1:4; 1:6; 1:8, and 1:10) were explored and evaluated by XRPD (Figure 6), keeping PGZ⋅HCl constant and varying FLV. 

**Figure 7:**
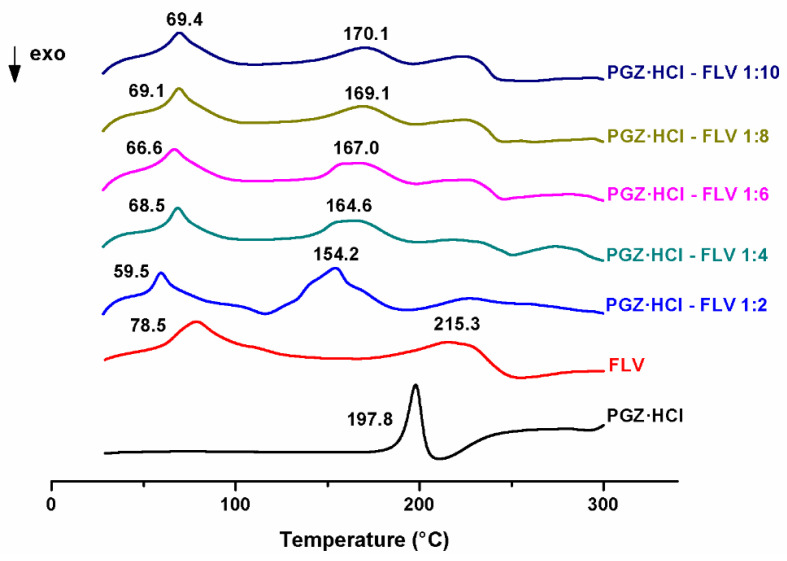
DSC thermograms of the solid forms of PGZ·HCl-FLV at different stoichiometric ratios (1:2; 1:4; 1:6; 1:8, and 1:10).

All proportions showed the presence of a halo in interval 2θ (~15–30°). Initially, these results indicate coamorphous formation due to amorphous contributions in all explored stoichiometric ratios; however, the evaluation of the DSC thermograms shows something different (Figure 7). Additionally, in XRPD, two bulges are observed at 2θ at 32 and 46° due to amorphous NaCl. The intensity of these decreases as the %w of FLV increases in the samples.

All individual DSC-TGA thermograms for these outcomes are found in Figure S7. Thermodynamic data of these solid forms are presented in Table 2. Based on the curve of the first derivative of the DSC, all the samples allowed us to see the events of T_g_ (Figure S7). 

All DSCs present a first endothermic event that, according to the TGA, is attributed to water molecules of hydration. Specifically, 1:2 stoichiometry shows a thermogram that contains T_g_: 42.4 °C, T_c_: (116.0 °C, exo), and T_m_: 154.2 °C. This thermogram profile is similar to that observed with the coamorphous 1:1 mixture (in the presence of T_g_, T_c_, and T_m_). Calculating the %crystallinity values of the components within the solid phase showed PGZ⋅HCl: 99.01% and FLV: 58.85% (Table 2). Given these results, since it is not observed, the relaxation enthalpy contribution must be superimposed onto the event corresponding to the thermal event of the water molecule of hydration. PGZ⋅HCl is almost completely recrystallized, and FLV presents a considerable advance. Therefore, the presence of water in hydration molecules is attributed to this recrystallized FLV. The Gordon–Taylor equation determined the T_g_ value for 1:2 stoichiometry at 67.86 °C. Therefore, the calculated value is above the experimental value. As previously mentioned, other factors not contemplated in the Gordon–Taylor equation must have an effect to prevent a good correlation of values. Among these is the effect of the enthalpic relaxation endotherm and water molecules accommodated within the solid phase. Subsequently, observing the second endothermic peak (154.2 °C), and as will be seen in Section 3.4, this 1:2 composition is a mixture of components since it presents an incongruous melting point (solidus) and excess amorphous FLV (liquidus). Therefore, it is considered a eutectic mixture.

In the case of the other molar ratios, two endothermic events (interval 154.6–170.1 °C) are observed again due to the incongruent melting point (solidus) and the excess of amorphous FLV (liquidus). This will be explained in more detail in Section 3.4. In these molar ratios, T_c_ events are no longer observed. T_g_ values were calculated but deviated above the experimental values (Table 2). Again, this discrepancy is attributed to factors not considered in the Gordon–Taylor equation. As the %w of FLV increases, the calculated value of T_g_ deviates greatly from the experimental value. Additionally, it is observed that as the proportion of FLV increases, the ΔH_m_ values decrease. 

**Figure 8:**
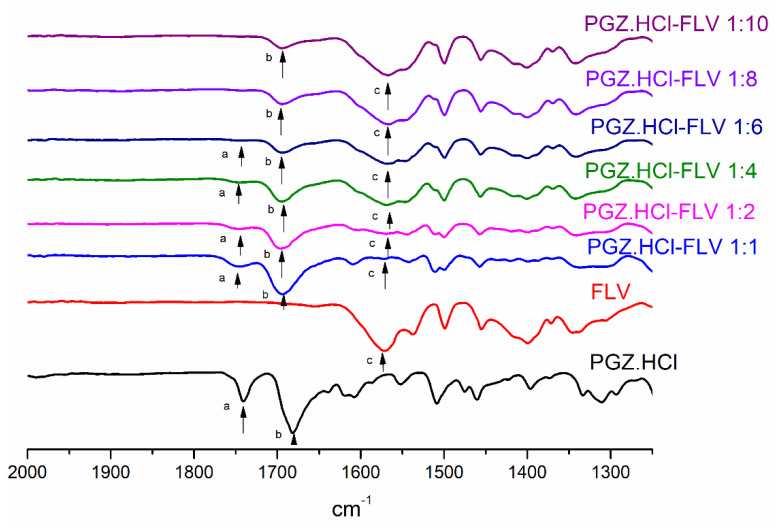
Expanded FT-IR spectra of the solid forms of PGZ·HCl−FLV at different stoichiometric ratios (1:1; 1:2; 1:4; 1:6; 1:8, and 1:10).

Analyzing all these stoichiometries utilizing FT-IR showed the following (Figure 8) (expanded in the interval of 2000–1300 cm^−1^). Full spectra are found in Figure S8. The vibrational modes −C=O_PGZ_ (1744 and 1690 cm^−1^) and −C=O_FLV_ (1576 cm^−1^) of the pure drugs were evaluated (Table 3). The spectrum of the coamorphous 1:1 mixture obtained by NG was added (Figure 8).

The vibration −C=O_PGZ_ (b) presents shift values comparing the pure drug with the solid forms (Δν_average_~11.6 cm^−1^). This indicates that there were changes in the molecular interactions involved. Additionally, as the %w of FLV in the samples increases, this vibration decreases in intensity. For −C=O_PGZ_ (a), changes in the participating molecular interactions cannot be attributed since the Δν values were low. Although gradually, as %w of FLV increases, the intensity of this band decreases. On the other hand, in the band corresponding to −C=O_FLV_ in the stoichiometries (1:1 and 1:2), the values of Δν indicate the formation of a new synthon; likewise, as the %w of FLV increases, the values of Δν decrease. Comparatively, in what was observed in the salt–cocrystal continuum, a shift (Δν) was detected at high frequencies. In the case of the coamorphous 1:1 mixture, the displacement was at low frequencies, which indicates that the deprotonation of the pyridinium fragment is not observed, and the carboxylate is not abstracting the proton. Therefore, the synthon that must prevail in the coamorphous 1:1 mixture should correspond to a salt −COO^−^⋅⋅⋅^+^H-N_pyr_ (Figure 3).

### 3.3. Evaluation of the Formation of the PGZ·HCl-FLV Solid Forms (2:1; 4:1; 6:1; 8:1 and 10:1)

**Figure 9:**
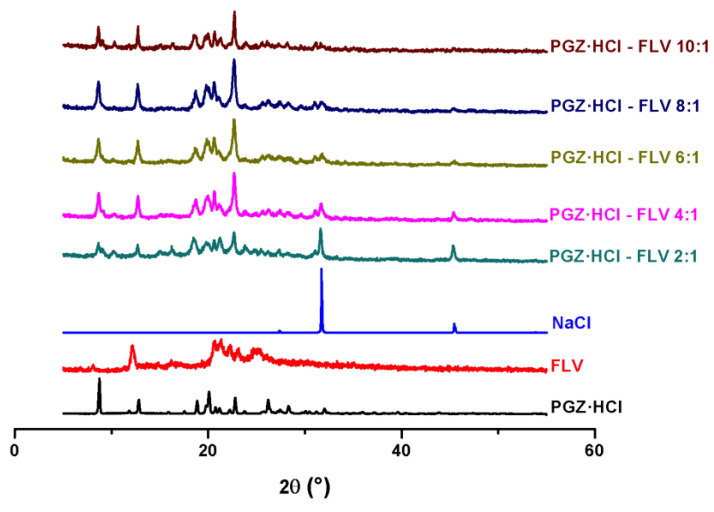
XRPD diffractograms of the solid forms of PGZ·HCl-FLV at different stoichiometric ratios (2:1; 4:1; 6:1; 8:1, and 10:1).

All these mechanochemically explored molar ratios were initially evaluated by XRPD (Figure 9) keeping FLV constant and varying PGZ⋅HCl. 

In the 2:1 stoichiometric ratio, it is possible to see the formation of NaCl. However, in the other ratios, as the %w of PGZ·HCl increases, the intensity of these reflections gradually decreases. In the first instance, the formation of the coamorphous mixture cannot be ascertained in all these stoichiometric ratios since the presence of a halo is not observed, and the characteristic reflections of PGZ⋅HCl are present. An attempt was made to amorphized pure PGZ⋅HCl by NG (150 min) (Figure S9). However, this was unsuccessful because this drug cannot be amorphized by ball milling. In the other proportions (4:1; 6:1; 8:1, and 10:1), it is seen that as the %w of PGZ⋅HCl increases, the intensity of its reflections also increases. 

**Figure 10:**
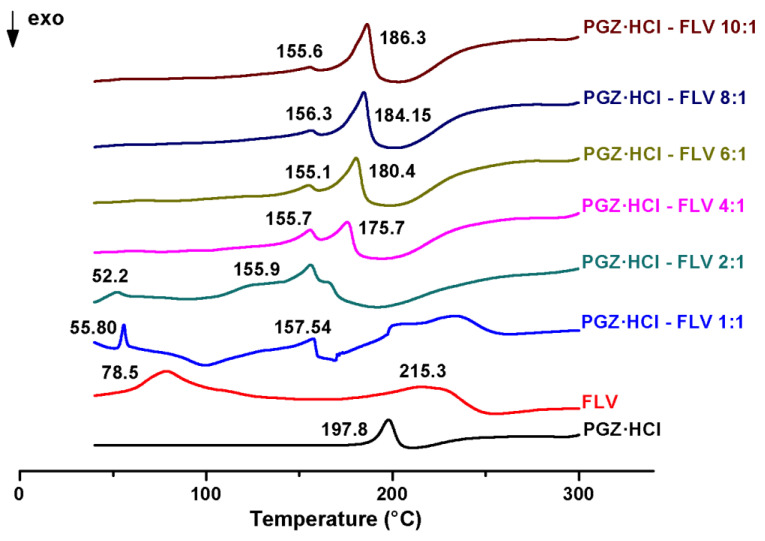
DSC thermograms of the solid forms of PGZ·HCl-FLV at different stoichiometric ratios (2:1; 4:1; 6:1; 8:1, and 10:1).

Regarding the DSC results, the following is observed in Figure 10 and Table 4. In addition, individual DSC-TGA thermograms are found in Figure S10.

At the 2:1 molar ratio, the glass transition event cannot be seen at first glance. However, this is achieved with the first derivative curve of the DSC (Figure S10). A T_g_ value (47.2 °C) is observed, followed by a second T_g_ (111.2 °C). The calculated T_g_ with the Gordon–Taylor equation, for the first value, is 38.86 °C, which does not correlate well with the experimental one. This may be due to the two thermal events (solidus and liquidus), implying that this composition is a eutectic mixture.

In the case of the other stoichiometries, it is not evident to observe the value of T_g_, but based on the curve of the first derivative of the DSC, it was possible to perceive it (Figure S10). In all cases (4:1 (54.5 and 105.1 °C); 6:1 (59.6 and 99.2 °C); 8:1 (52.6 and 105.0 °C); and 10:1 (52.0 and 109.2 °C)), the presence of two glass transition events can be seen. Additionally, as described in Section 3.4, all these molar ratios are eutectic mixtures, showing an incongruous melting point (solidus) and excess of PGZ·HCl. As the %w of PGZ·HCl increases in each sample, the value of ΔH_m_ corresponding to an excess of this drug gradually increases (4:1 (54.5 J/g); 6:1 (79.7 J/g); 8:1 (88.69 J/g); and 10:1 (95.0 J/g).

**Figure 11:**
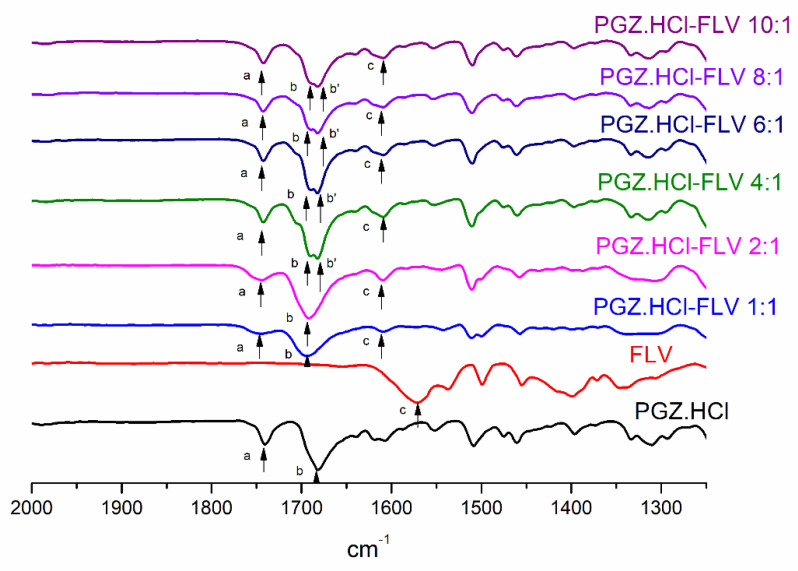
Extended FT-IR spectra of the solid forms of PGZ·HCl-FLV at different stoichiometric ratios (1:1; 2:1; 4:1; 6:1; 8:1, and 10:1).

FT-IR also analyzed all these molar ratios to observe the changes in the intermolecular interactions that are participating between the components. Figure 11 shows an expansion in the range of 2000–1300 cm^−1^. Table 5 shows the frequency values of the vibration modes. The full spectra of all these samples are found in Figure S11. In the case of the vibration −C=O_FLV_, a shift is observed comparing the values of the pure drug with the solid binary forms (interval Δν = 36–37 cm^−1^). 

On the other hand, in the case of the −C=O _PGZ_ vibrational mode, the Δν values are very low, except for the 2:1 and 6:1 stoichiometry, where Δν values of 10 are observed, indicating a change in the participating molecular interactions. However, as the stoichiometric ratio of PGZ⋅HCl increases, the 1682 cm^−1^ band is a doublet, possibly due to incongruous melting and excess of pioglitazone since they are eutectic mixtures. 

### 3.4. Eutectic Screening to Predict the Most Stable Coamorphous Molar Ratio

Previously, it has been seen that by performing eutectic screening at different drug-to-drug ratios using DSC, it is possible to determine the stoichiometric ratio to form the most stable coamorphous proportion [58]. The systems studied were indomethacin–naproxen, nifedipine–paracetamol, and paracetamol–celecoxib. In this paper, it has been observed that the respective drug–drug ratio that forms the eutectic point corresponds to the most stable coamorphous form.

In our case, building a binary phase diagram makes it possible to determine the most stable molar ratio of the coamorphous mixture [24,25]. As mentioned in Section 2.2.11, for molar proportions 2:1; 4:1; 6:1; 8:1, and 10:1, the appearance of two endothermic events (solidus and liquidus points) was clear (Figure S10). However, molar ratios 1:1; 1:2; 1:4; 1:6; 1:8; 1:10 were run at 2 °C/min to improve the differentiation of solidus and liquidus events. The thermograms of these proportions with a heating rate of 2 °C/min are presented in Figure S12; the binary phase diagram is also shown in this figure. However, it was impossible to effectively separate the two thermal events (solidus and liquidus) after analyzing the samples with this new heating rate. We attribute this to the fact that the contribution of amorphous FLV in the different outcomes does not allow the separation of the two events. Since, as observed in the molar ratios where PGZ·HCl is in excess and in a semi-crystalline state, these two endotherms can be separated. Not being able to effectively separate the solidus and liquidus events affected obtaining the phase diagram with the typically expected V shape.(Figure S12). Despite this, it can be confirmed that the stoichiometric proportion 1:1 is the most stable molar ratio to form the coamorphous mixture. This is because the incongruent melting point (solidus) and the excess of components (liquidus) are not observed in this ratio. Otherwise, in the other proportions where both events are detected, Tammann’s plot triangle is very helpful in confirming that a genuine eutectic composition was found in the binary phase diagrams [26]. With this graph, it was possible to corroborate that the 1:1 molar ratio is the most stable composition of the coamorphous mixture (Figure S12).

### 3.5. SEM

The grain morphology of the solid forms (1:1; 1:6; 1:10; 6:1; and 10:1) was inspected and compared with the pure drugs (Table 6). SEM images are shown in Figure S13.

The grain morphology of the pure drugs are flakes for FLV and prismatic forms for PGZ·HCl. In the case of the coamorphous salt in a 1:1 ratio, it does not present a defined grain morphology. The observed grains are poorly defined and have very compact prismatic shapes. On the other hand, if the %w of FLV is increased (1:6; and 1:10), two types of grains are observed. In the 1:6 molar ratio, they present prismatic grains of different sizes. However, in the case of the 1:10 ratio, rough shapes are shown to be mixed with flakes. On the other hand, when the %w of PGZ·HCl increases (6:1 and 10:1), the following is observed: the solid binary form 6:1 shows a mix of well-defined prismatic shapes with irregular prismatic. Additionally, for the 10:1 ratio, most of the grains are poorly prismatically defined. In either of these two forms, mixtures of two types of grain are not observed.

### 3.6. Determination of Dissolution Profiles

**Figure 12:**
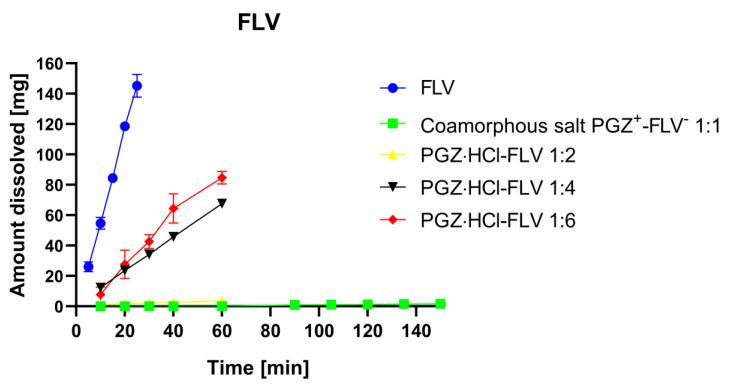
Dissolution profiles of pure FLV, coamorphous PGZ−FLV (1:1), and solid forms of PGZ⋅HCl−FLV (1:2; 1:4; and 1:6).

Dissolution studies were carried out on the pure FLV and the solid forms of PGZ⋅HCl-FLV (1:2; 1:4; and 1:6), together with the coamorphous mixture (1:1) (Figure 12). Figure S14 shows the plots with another scale on the y-axis to observe the dissolution profiles in more detail. Pure water was used as the dissolution medium as recommended by the Pharmacopoeia of the United Mexican States (PUMS) 13th edition [23]. Initially, an attempt was made to determine the amount of dissolved FLV in the dissolution profiles of the solid forms where the %w of PGZ⋅HCl was varied (2:1; 4:1; 6:1; 8:1, and 10:1), but it was impossible to quantify since the tablets were insoluble in water. Likewise, it was impossible to determine the corresponding quantity of dissolved FLV for the solid forms of PGZ⋅HCl-FLV (1:8; and 1:10), since the tablet dissolved immediately when in contact with the medium, not allowing quantifications to be carried out. Therefore, the K_int_ values are presented in Table 7.

Amorphous solid forms can provide faster dissolution rates and higher solution concentrations than their crystalline analogs [15,59,60,61]. In general, crystalline forms of drugs are thermodynamically more stable (higher density and melting point) and consequently have higher free energy and lower solubility than their amorphous counterparts [61]. In this case, when the coamorphous mixtures are formed, they will present decreased crystal lattice energy values compared to the initial components. This modification in the energy of the crystalline lattice alters the crystalline packing, contributing significantly to improving solubility since the molecules are randomly oriented because these systems lack an ordered crystalline lattice. This situation easily favors the release of isolated molecules. This produces a supersaturation once the coamorphous form is introduced into the aqueous medium since large amounts of the drug are released into the solution. This is known as the “spring effect”. The duration of this effect depends on the drug’s tendency to recrystallize in the solution. This supersaturation situation can be limited if the transformation process from the amorphous state of the drug to the crystalline state is very fast [62]. Otherwise, this supersaturation can benefit when the amorphous → crystal transformation occurs more slowly, known as the “parachute effect”. The prolongation of the parachute effect in coamorphous systems will depend on the nature of the second component. This can delay and prevent the first component’s nucleation and crystal growth, slowing the crystallization process [15]. Thus, this “spring–parachute” concept may explain the solubility advantage of the pharmaceutical coamorphous mixture. The coamorphous mixture dissociates into amorphous or nanocrystalline drug clusters (the “spring effect”), which are transformed through rapidly dissolving metastable polymorphs towards forming a stable crystalline phase following the stages of Ostwald’s law [59]. 

The value determined by the K_int_ of pure FLV (13.6270 ± 0.8127 mg/cm^2^·min) indicates that this drug dissolves quickly in water. The slope is very steep, and this can be considered the “spring effect” [59]. After 25 min, approximately 145 mg of FLV has been released. In the case of the other solid forms, the evaluation of the IDR studies must be analyzed separately. 

In the coamorphous 1:1 salt, the dissolution profile exhibits a very low K_int_ (0.0220 ± 0.0014 mg/cm^2^⋅min). Initially, this profile was not what was expected since, as mentioned above, the coamorphous forms present a reduction in the crystalline lattice values (ΔH_m_: coamorphous 1:1 salt: 38.73 J/g vs. pure FLV: 97.00 J/g, Table 1) which facilitates the release and sudden enrichment of the drug concentration in the medium. This indicates that what is probably happening is that the transformation of an amorphous → crystal FLV occurs very fast. Therefore, it has been proposed that the mechanism of prolongation of the supersaturation situation for polymeric solid dispersions can present three scenarios [63].

(A) The drug dissolves and is released rapidly into the solution, precipitously raising the concentration of molecules, and subsequently, the drug is precipitated by the amorphous → crystal transformation.
(B) Simultaneously, the drug and polymer are progressively released, while the drug remains amorphous on the surface of the undissolved particles.
(C) The drug and polymer are progressively released; however, the drug is in the form of crystals on the surface of the undissolved particles.

Within these three scenarios, it is highlighted that the polymer plays an important role since it can act as a “retarder” or “accelerator” of the amorphous → crystal transformation. Additionally, this largely depends on drug–polymer interactions (the formation and breaking of hydrogen bonds and hydrophobic interactions). This is important since the contribution of these interactions can retard or accelerate the nucleation and crystal growth process. Additionally, the degree of lipophilicity that the polymer presents can accelerate or retard this.

Although these scenarios are proposed for solid dispersion polymer systems, scenario (C) explains what is happening in our system. It must not be forgotten that coamorphous mixtures fall within the families of solid dispersions [64]. 

For coamorphous mixtures, it has been mentioned that when both components present strong intermolecular interactions for binary systems, the coformer can facilitate the dissolution of the poorly soluble drug. Thus, the dissolution rate of the poorly soluble component depends on the solubility of the coformer [65]. In this sense, PGZ⋅HCl presents solubility at a pH 7.39 of the glycine buffer for 0.020 mmol/L and 0.033 mmol/L of the phosphate buffer (pH 7.40), revealing that the solubility is dependent on the type of buffer and thus the pH value [66]. Thus, PGZ·HCl is class II according to BCS and is insoluble in water [67]. On the other hand, it has been reported that the hydrochloride salt form of the free base of PGZ increases its solubility in water [68]. Therefore, although the solubility value of the free base has not been reported, it should be considered much lower than the hydrochloride salt. Based on this, the salt–cocrystal continuum presents strong intermolecular interactions compared to the coamorphous 1:1 salt (ΔH_m_: 223.6 vs. 38.73 J/g, Table 1). In this way, it must be considered that in the coamorphous form, what is released is H-PGZ^+^. Its low solubility accelerates the nucleation of the salt–cocrystal continuum, accommodating this crystalline phase in the undissolved particles. This prevents FLV from being released. Something interesting to note is that FLV release is not observed during the first 60 min (Figure S14); however, after this time, this drug is released very slowly (150 min, ~2 mg). Therefore, it can be argued that a certain amount of the crystallized salt–cocrystal continuum on the surface of the undissolved particles redissolves, releasing FLV.

In the case of the other composition ratio (1:2; a eutectic mixture), FLV’s release behavior was poor, as observed with the coamorphous 1:1 salt, presenting a K_int_ value of 0.1057 ± 0.0113 mg/cm^2^⋅min. It should be clarified that we do not have a clear idea because even when this eutectic form contains an excess of FLV, it does not allow an increase in the release of this drug compared to the coamorphous form. Therefore, it is proposed that H-PGZ^+^ is released rapidly, as was observed in the dissolution profile of the coamorphous salt form. Additionally, due to its low solubility in the medium, it accelerates the nucleation process to form the salt–cocrystal continuum. For this reason, such a poor supersaturation effect is observed.

Nevertheless, a different profile is observed in solid forms of PGZ·HCl-FLV (1:4; and 1:6). These binary compositions are eutectic mixtures where they present excess FLV in the amorphous state. This excess amorphous FLV is released into the aqueous medium. As the %w of FLV in each solid phase increases, the value of K_int_ also increases. It must be emphasized here that the release behavior of FLV in these solid forms differs from that seen with coamorphous 1:1 salt or a 1:2 molar ratio. In the solid forms of PGZ⋅HCl-FLV (1:4 and 1:6), the %w of FLV is in much greater excess than the other two molar ratios. In this case, in these compositions, they are released as Fluvastatin sodium salt, and the Na^+^ counterion does not accelerate the recrystallization of the drug; otherwise, in the other solid forms where the H-PGZ^+^ is found in a greater proportion, and since it has limited solubility in water, it does speed up this process. 

Given this, pure FLV had a dissolution profile considered a “spring effect”. However, the coamorphous 1:1 salt, apparently the great advance of enthalpic relaxation, indicates that the components are in an advanced state of recrystallization. Therefore, when the coamorphous 1:1 salt enters the dissolution medium, this solid form crystallizes quickly, presenting a very poor FLV release process. On the other hand, in the case of the other solid forms, their FLV release behavior was dependent on the %w content.

## 4. Conclusions

Ball milling is a versatile synthetic tool since, depending on the NG or LAG conditions, a great diversity of solid forms was obtained in the binary PGZ⋅HCl and FLV systems, specifically in the stoichiometric ratio 1:1. Through ball milling, it was possible to access different solid forms: NG and hexane (coamorphous 1:1 salt); AcOEt (physical mixture); EtOH (salt–cocrystal continuum), and water (which presents two T_g_, indicating immiscibility of the components). Attempts to obtain the coamorphous 1:1 salt by extending the ball-milling times starting from the salt–cocrystal continuum were unsuccessful.

Exploring other molar ratios applying NG, it was demonstrated that eutectic mixtures were obtained because an incongruous melting point (solidus) and an excess of one of the components (liquidus) were observed, except for in the 1:1 solid form, which turned out to be the most stable coamorphous composition.

On the other hand, pure FLV had a dissolution profile considered a “spring effect.” Unfortunately, the coamorphous 1:1 salt and the composition 1:2 salt, apparently the state of recrystallization that the components present, coupled with the insolubility of PGZ·HCl, accelerate the transformation process, which makes FLV’s release behavior very poor. On the other hand, in the case of the other solid forms, their FLV release behavior was dependent on the %w content.

It is proposed that PGZ·HCl did not become a suitable coformer in the formation of coamorphous salt in this system (PGZ·HCl and FLV). Due to these results, the coamorphous salt form 1:1 was formed. Still, this composition presents enthalpic relaxation between the components. 

## Figures and Tables

**Table 1:** Thermodynamic data of the different outcomes prepared by NG or LAG solvent screening.

Outcome NG or LAG Solvent Screening	PGZ⋅HCl (mg)	FLV (mg)	PGZ⋅HCl (%w)	FLV (%w)	T_first peak_ (°C)	T_onset second peak_ (°C)	T_m second peak_ (°C)	ΔH_m second peak_ J/g	T_g exp_/T_g clcd_ °C	%Cristallinity PGZ⋅HCl	%Cristallinity FLV
PGZ⋅HCl	-	-	-	-	-	190.0	197.8	125.5	64.4 [19]	-	-
FLV	-	-		-	78.6	186.2	215.6	97.0	69.5 [10]	-	-
NG	97.46	107.49	46.86	53.14	T_c_: 95.8 exo	T_m_: 140.6	T_m_: 154.5	38.73	56.2/67.07	65.85	75.13
Hexane	97.46	107.49	46.86	53.14	-	T_m_: 135.5	T_m_: 155.3	46.16	61.7/67.07	78.49	89.55
AcOEt	97.46	107.49	46.86	53.14	T_m_: 115.2	-	T_m_: 156.9	98.8	-	-	-
EtOH	97.46	107.49	46.86	53.14	-	188.4	196.5	223.6	108.8/44.28		
Water	97.46	107.49	46.86	53.14	-	136.8	156.8	47.95	71.1 and 93.5/44.28	-	-

**Table 2:** Thermodynamic data of the different solid forms of PGZ·HCl-FLV at different stoichiometric ratios (1:2; 1:4; 1:6; 1:8, and 1:10).

Stoichiometric Ratios	PGZ⋅HCl (mg)	FLV (mg)	PGZ⋅HCl (%w)	FLV (%w)	T_fus first peak_(°C)	T_c_ (°C)	T_onset melting_ (°C)	T_m_ (°C)	ΔH_m_ J/g	T_g exp_/ T_g clcd_ °C	%Cristallinity PGZ⋅HCl	%Cristallinity FLV
1:2	94.5	205.7	31.48	68.52	59.3	116.0	132.2	154.2	39.12	42.4/67.86	99.01	58.85
1:4	58.3	252.7	18.75	81.25	68.5	-	119.4	164.6	64.71	46.0/68.52	-	-
1:6	47.4	304.5	13.47	86.53	66.6	-	135.3	167.0	54.68	48.7/68.79	-	-
1:8	33.9	267.3	11.25	88.75	69.1	-	122.5	169.1	52.59	49.9/68.91	-	-
1:10	31.5	332.0	8.67	91.33	69.4	-	119.8	170.1	48.2	50.9/69.04	-	-

**Table 3:** Vibrational band assignment of the solid forms of PGZ·HCl-FLV at different stoichiometric ratios (1:1; 1:2; 1:4; 1:6; 1:8, and 1:10).

Vibrational Band Assignment	PGZ⋅HCl	FLV	PGZ⋅HCl-FLV (1:1)	PGZ⋅HCl-FLV (1:2)	PGZ⋅HCl-FLV (1:4)	PGZ⋅HCl-FLV (1:6)	PGZ⋅HCl-FLV (1:8)	PGZ⋅HCl-FLV (1:10)
−C=O_PGZ_ (a, b) (Δν cm^−1^)	a: 1741 b: 1682		a: 1743 (2) b: 1693 (9)	a: 1744 (3) b: 1697 (15)	a: 1743 (2) b: 1695 (13)	a: 1740 (1) b: 1693 (11)	a: 1740 (1) b: 1693 (11)	a: 1740 (1) b: 1693 (11)
−C=O_FLV_ (c) (Δν cm^−1^)		1572	1548 (24)	1546 (26)	1568 (8)	1565 (7)	1567 (5)	1565 (7)

|Δν cm^−1^| = vibration mode of pure drug—vibration mode of solid binary form.

**Table 4:** Thermodynamic data of the different solid forms of PGZ·HCl-FLV at different stoichiometric ratios (2:1; 4:1; 6:1; 8:1, and 10:1).

Stoichiometric Ratios	PGZ⋅HCl (mg)	FLV (mg)	PGZ⋅HCl (%w)	FLV (%w)	T_fus first peak_ (°C)	T_onset second peak_ (°C)	T_m second peak_ (°C)	ΔH_m second peak_ J/g	T_g exp_/T_g clcd_ °C
2:1	202.7	113.9	64.02	35.98	-	N.D.	155.9	18.5	47.2/66.20
4:1	251.6	69.6	78.33	21.67	155.7	N.D.	155.7	14.9	54.5/65.48
6:1	302.7	57.7	83.99	16.01	155.1	N.D.	155.1	9.34	59.6/65.19
8:1	303.5	43.5	87.46	12.54	156.3	N.D.	156.3	6.53	52.6/65.02
10:1	302.6	34.9	89.66	10.34	155.6	N.D.	155.6	5.28	52.0/64.91

N.D. = not defined.

**Table 5:** Vibrational band assignment of the solid forms of PGZ·HCl-FLV at different stoichiometric ratios (2:1; 4:1; 6:1; 8:1, and 10:1).

Vibrational Band Assignment	PGZ⋅HCl	FLV	PGZ⋅HCl-FLV (2:1)	PGZ⋅HCl-FLV (4:1)	PGZ⋅HCl-FLV (6:1)	PGZ⋅HCl-FLV (8:1)	PGZ⋅HCl-FLV (10:1)
−C=O_PGZ_ (a,b,b’) (Δν cm^−1^)	a: 1741 b: 1682		a: 1742 (1) b: 1692 (10)	a: 1742 (1) b: 1682 (0) b’: 1680 (2)	a: 1743 (2) b: 1682 (10) b’: 1680 (2)	a: 1743 (2) b: 1682 (0) b’: 1678 (4)	a: 1743 (2) b: 1684 (2) b’: 1680 (2)
−C=O_FLV_ (c) (Δν cm^−1^)		1572	1609 (37)	1609 (37)	1608 (36)	1609 (37)	1608 (36)

|Δν cm^−1^| = vibration mode of pure drug—vibration mode of solid binary form.

**Table 6:** Grain morphology of the solid forms (1:1; 1:6; 1:10; 6:1; and 10:1).

.	Pure FLV	Pure PGZ⋅HCl	Coamorphous Salt (1:1)	PGZ⋅HCl-FLV (1:6)	PGZ⋅HCl-FLV (1:10)	PGZ⋅HCl-FLV (6:1)	PGZ⋅HCl-FLV (10:1)
Morphology	Flakes	Prism shaped	Compacted poor defined prismatic forms	Prismatic forms with different sizes	Rough shapes mixed with flakes	A mix of well-defined prismatic shapes with irregular prismatic	Prism-shaped poorly defined

**Table 7:** K_int_ values for pure FLV, coamorphous PGZ-FLV (1:1; and 1:2), and solid forms of PGZ⋅HCl-FLV (1:4; and 1:6).

	Pure FLV	Coamorphous Salt PGZ-FLV (1:1)	PGZ⋅HCl-FLV (1:2)	PGZ⋅HCl-FLV (1:4)	PGZ⋅HCl-FLV (1:6)
K_int_ mg/cm^2^⋅min^−1^	13.6270 ± 0.8127	0.0220 ± 0.0014	0.1057 ± 0.0113	2.4953 ± 0.0309	3.5049 ± 0.3547

All values are associated with their corresponding standard deviation.

## Data Availability

No new data were created or analyzed in this study. Data sharing is not applicable to this article.

## Supplementary Materials

The following are available online at https://www.mdpi.com/article/10.3390/ph16060781/s1 (Figure S1). Individual DSC-TGA thermograms of the solid phases PGZ⋅HCl-FLV (1:1) prepared by NG or LAG solvent screening. Additionally, individual DSC-TGA thermograms of pure PGZ⋅HCl and FLV (Figure S2). Diffractograms of the salt PGZ^+^ -FLV^−^ (1:1, EtOH) extending the reaction times (Figure S3). HSQC ^1^H-^15^N NMR spectrum of PGZ∙HCl in d_6_-DMSO (Figure S4). HMBC ^1^H-^15^N NMR spectrum of PGZ∙HCl in d_6_-DMSO (Figure S5). XPS spectra of PGZHCl, FLV and the binary solid form (EtOH) (Figure S6). Full and extended PGZHCl, FLV, and PGZ-FLV (EtOH) FTIR spectra. Likewise, the table indicates the frequency values (Figure S7). Individual DSC-TGA thermograms of the solid forms of PGZ·HCl-FLV at different stoichiometric ratios (1:2; 1:4; 1:6; 1:8, and 1:10) (Figure S8). Full FT-IR spectra of the solid forms of PGZ·HCl-FLV at different stoichiometric ratios (1:1; 1:2; 1:4; 1:6; 1:8 and 1:10) (Figure S9). Evaluation of the amorphization ability of the PGZ·HCl (Figure S10). Individual DSC-TGA thermograms of the solid forms of PGZ·HCl-FLV at different stoichiometric ratios (2:1; 4:1; 6:1; 8:1 and 10:1) (Figure S11). Full FT-IR spectra of the solid forms of PGZ·HCl-FLV at different stoichiometric ratios (2:1; 4:1; 6:1; 8:1 and 10:1). Figure S12. PGZ·HCl-FLV binary phase diagram. Thermograms corresponding to the molar ratios 1:1; 1:2; 1:4; 1:6; 1: and 1:10 at 2 °C/min. Figure S13. Grain morphology images of the solid forms (1:1; 1:6; 1:10; 6:1; and 10:1) (Figure S14). Dissolution profiles with another scale on the y-axis show the plots in more detail.

## References

[1] Koch C. (2012). Statin Therapy. Curr. Pharm. Des..

[2] Borgmann S.H.M., Bernardi L.S., Rauber G.S., Oliveira P.R., Campos C.E.M., Monti G., Cuffini S.L., Cardoso S.G. (2013). Solid-state characterization and dissolution properties of Fluvastatin sodium salt hydrates. Pharm. Dev. Technol..

[3] Plosker G.L., Wagstaff A.J. (1996). Fluvastatin. Drugs.

[4] Balakumar P., Mahadevan N. (2012). Interplay between statins and PPARs in improving cardiovascular outcomes: A double-edged sword?. Br. J. Pharmacol..

[5] Tonstad S., Retterstøl K., Ose L., Öhman K.P., Lindberg M.B., Svensson M. (2007). The dual peroxisome proliferator-activated receptor α/γ agonist tesaglitazar further improves the lipid profile in dyslipidemic subjects treated with atorvastatin. Metabolism.

[6] Barilla D., Prasad P., Hubert M., Gumbhir-Shah K. (2004). Steady-state pharmacokinetics of fluvastatin in healthy subjects following a new extended release fluvastatin tablet, Lescol® XL. Biopharm. Drug Dispos..

[7] Singh S., Sharma N., Kaur G. (2020). Central composite designed solid dispersion for dissolution enhancement of fluvastatin sodium by kneading technique. Ther. Deliv..

[8] Sharma N., Singh S., Kaur G., Arora S. (2019). Preformulation studies of fluvastatin sodium with polyvinyl pyrollidone K-30 and polyethylene glycol 6000. Plant Arch..

[9] El-Helw A.R.M., Fahmy U.A. (2015). Improvement of fluvastatin bioavailability by loading on nanostructured lipid carriers. Int. J. Nanomed..

[10] Papageorgiou G., Papadimitriou S., Karavas E., Georgarakis E., Docoslis A., Bikiaris D. (2009). Improvement in Chemical and Physical Stability of Fluvastatin Drug Through Hydrogen Bonding Interactions with Different Polymer Matrices. Curr. Drug Deliv..

[11] Panda H.S., Srivastava R., Bahadur D. (2009). In-vitro release kinetics and stability of anticardiovascular drugs-intercalated layered double hydroxide nanohybrids. J. Phys. Chem. B.

[12] Solares-Briones M., Coyote-Dotor G., Páez-Franco J.C., Zermeño-Ortega M.R., de la O Contreras C.M., Canseco-González D., Avila-Sorrosa A., Morales-Morales D., Germán-Acacio J.M. (2021). Mechanochemistry: A Green Approach in the Preparation of Pharmaceutical Cocrystals. Pharmaceutics.

[13] Muñoz Tecocoatzi M.F., Páez-Franco J.C., Coyote-Dotor G., Dorazco-González A., Miranda-Ruvalcaba R., Morales-Morales D., Germán-Acacio J.M. (2022). Mecanoquímica: Una herramienta importante en la reactividad en el Estado Sólido Mechanochemistry: An important tool in solid-state reactivity. Tecnociencia Chihuahua.

[14] Karagianni A., Kachrimanis K., Nikolakakis I. (2018). Co-Amorphous Solid Dispersions for Solubility and Absorption Improvement of Drugs: Composition, Preparation, Characterization and Formulations for Oral Delivery. Pharmaceutics.

[15] Shi Q., Moinuddin S.M., Cai T. (2019). Advances in coamorphous drug delivery systems. Acta Pharm. Sin. B.

[16] Chavan R.B., Thipparaboina R., Kumar D., Shastri N.R. (2016). Co amorphous systems: A product development perspective. Int. J. Pharm..

[17] Zakeri-Milani P., Barzegar-Jalali M., Azimi M., Valizadeh H. (2009). Biopharmaceutical classification of drugs using intrinsic dissolution rate (IDR) and rat intestinal permeability. Eur. J. Pharm. Biopharm..

[18] Newman A., Zografi G. (2020). Commentary: Considerations in the Measurement of Glass Transition Temperatures of Pharmaceutical Amorphous Solids. AAPS PharmSciTech.

[19] Kapourani A., Vardaka E., Katopodis K., Kachrimanis K., Barmpalexis P. (2020). Crystallization tendency of APIs possessing different thermal and glass related properties in amorphous solid dispersions. Int. J. Pharm..

[20] https://www.chemsrc.com/en/cas/93957-54-1_237852.html.

[21] International Agency for Research on Cancer, WHO (2016). Pioglitazone and Rosiglitazone.

[22] Rawlinson C.F., Williams A.C., Timmins P., Grimsey I. (2007). Polymer-mediated disruption of drug crystallinity. Int. J. Pharm..

[23] Secretaría de Salud, Comisión Permanente de la Farmacopea de los Estados Unidos Mexicanos (2022). Farmacopea de los Estados Unidos Mexicanos (FEUM).

[24] Rycerz L. (2013). Practical remarks concerning phase diagrams determination on the basis of differential scanning calorimetry measurements. J. Therm. Anal. Calorim..

[25] Coyote-Dotor G., Páez-Franco J.C., Canseco-González D., Núñez-Pineda A., Dorazco-González A., Fuentes-Noriega I., Vilchis-Néstor A.R., Rodríguez-Hernández J., Morales-Morales D., Germán-Acacio J.M. (2021). Synthesis, Characterization, and Intrinsic Dissolution Studies of Drug—Drug Eutectic Solid Forms of Metformin Hydrochloride and Thiazide Diuretics. Pharmaceutics.

[26] Chadha K., Karan M., Chadha R., Bhalla Y., Vasisht K. (2017). Is Failure of Cocrystallization Actually a Failure? Eutectic Formation in Cocrystal Screening of Hesperetin. J. Pharm. Sci..

[27] version 9.6.0.15, 2015-02-19, Ulvac-phi. Multipack.

[28] Crist B.V. 17 January 2004 SDP v 4.1 (32 bit) Copyright ©2004, XPS International, LLC., Compiled 17 January 2004. http://www.xpsdata.com.

[29] Poornima B., Prasad K.V.S.R.G., Bharathi K. (2017). Solid-State Screening and Evaluation of Pioglitazone Hydrochloride. Curr. Pharm. Anal..

[30] Newman A., Engers D., Bates S., Ivanisevic I., Kelly R.C., Zografi G. (2008). Characterization of amorphous API:Polymer mixtures using X-ray powder diffraction. J. Pharm. Sci..

[31] Shamblin S.L., Zografi G. (1998). Enthalpy relaxation in binary amorphous mixtures containing sucrose. Pharm. Res..

[32] Su M., Xia Y., Shen Y., Heng W., Wei Y., Zhang L., Gao Y., Zhang J., Qian S. (2019). A novel drug-drug coamorphous system without molecular interactions: Improve the physicochemical properties of tadalafil and repaglinide. RSC Adv..

[33] Jensen K.T., Blaabjerg L.I., Lenz E., Bohr A., Grohganz H., Kleinebudde P., Rades T., Löbmann K. (2016). Preparation and characterization of spray-dried co-amorphous drug–amino acid salts. J. Pharm. Pharmacol..

[34] Shayanfar A., Jouyban A. (2013). Drug-drug coamorphous systems: Characterization and physicochemical properties of coamorphous atorvastatin with carvedilol and glibenclamide. J. Pharm. Innov..

[35] Shamblin S.L., Huang E.Y., Zografi G. (1996). The effects of co-lyophilized polymeric additives on the glass transition temperature and crystallization of amorphous sucrose. J. Therm. Anal..

[36] Wu W., Wang Y., Löbmann K., Grohganz H., Rades T. (2019). Transformations between Co-Amorphous and Co-Crystal Systems and Their Influence on the Formation and Physical Stability of Co-Amorphous Systems. Mol. Pharm..

[37] Friščić T. (2010). New opportunities for materials synthesis using mechanochemistry. J. Mater. Chem..

[38] Jayasankar A., Somwangthanaroj A., Shao Z.J., Rodríguez-Hornedo N. (2006). Cocrystal formation during cogrinding and storage is mediated by amorphous phase. Pharm. Res..

[39] Seefeldt K., Miller J., Alvarez-Nunez F., Rodriguez-Hornedo N. (2007). Crystallization Pathways and Kinetics of Carbamazepine-Nicotinamide Cocrystals from the Amorphous State by In Situ Thermomicroscopy, Spectroscopy, and Calorimetry Studies. J. Pharm. Sci..

[40] Tothadi S., Shaikh T.R., Gupta S., Dandela R., Vinod C.P., Nangia A.K. (2021). Can We Identify the Salt–Cocrystal Continuum State Using XPS?. Cryst. Growth Des..

[41] Al-Majed A., Bakheit A.H.H., Abdel Aziz H.A., Alharbi H., Al-Jenoobi F.I. (2016). Chapter Five—Pioglitazone. Profiles of Drug Substances, Excipients and Related Methodology.

[42] Levy G.C., Lichter R.L. (1979). Nitrogen-15 Nuclear Magnetic Resonance Spectroscopy.

[43] Stevens J.S., Byard S.J., Seaton C.C., Sadiq G., Davey R.J., Schroeder S.L.M. (2014). Proton transfer and hydrogen bonding in the organic solid state: A combined XRD/XPS/ssNMR study of 17 organic acid-base complexes. Phys. Chem. Chem. Phys..

[44] Skorupska E., Kaźmierski S., Potrzebowski M.J. (2017). Solid State NMR Characterization of Ibuprofen: Nicotinamide Cocrystals and New Idea for Controlling Release of Drugs Embedded into Mesoporous Silica Particles. Mol. Pharm..

[45] Cerreia Vioglio P., Catalano L., Vasylyeva V., Nervi C., Chierotti M.R., Resnati G., Gobetto R., Metrangolo P. (2016). Natural Abundance15N and13C Solid-State NMR Chemical Shifts: High Sensitivity Probes of the Halogen Bond Geometry. Chem. A. Eur. J..

[46] Li P., Chu Y., Wang L., Yu K., Zhang H., Deng Z. (2014). Structure determination of the theophylline—Nicotinamide cocrystal: A combined powder calculation study. CrystEngComm.

[47] Solum M.S., Altmann K.L., Strohmeier M., Berges D.a., Zhang Y., Facelli J.C., Pugmire R.J., Grant D.M. (1997). 15N Chemical Shift Principal Values in Nitrogen Heterocycles Chemical Shift Principal Values in Nitrogen Heterocycles. J. Am. Chem. Soc..

[48] Marek R., Lycka A., Kolehmainen E., Sievänen E., Tousek J. (2007). 15N NMR Spectroscopy in Structural Analysis: An Update (2001–2005). Curr. Org. Chem..

[49] Li Z.J., Abramov Y., Bordner J., Leonard J., Medek A., Trask A. (2006). V Solid-State Acid−Base Interactions in Complexes of Heterocyclic Bases with Dicarboxylic Acids:  Crystallography, Hydrogen Bond Analysis, and 15N NMR Spectroscopy. J. Am. Chem. Soc..

[50] Zhao L., Hanrahan M.P., Chakravarty P., DiPasquale A.G., Sirois L.E., Nagapudi K., Lubach J.W., Rossini A.J. (2018). Characterization of Pharmaceutical Cocrystals and Salts by Dynamic Nuclear Polarization-Enhanced Solid-State NMR Spectroscopy. Cryst. Growth Des..

[51] Bolla G., Nangia A. (2018). Novel pharmaceutical salts of albendazole. CrystEngComm.

[52] Stevens J.S., Byard S.J., Schroeder S.L.M. (2010). Salt or Co-Crystal? Determination of Protonation State by X-Ray Photoelectron Spectroscopy (XPS). J. Pharm. Sci..

[53] Childs S.L., Stahly G.P., Park A. (2007). The Salt–Cocrystal Continuum: The Influence of Crystal Structure on Ionization Stat. Mol. Pharm..

[54] https://go.drugbank.com/salts/DBSALT000555.

[55] https://pubchem.ncbi.nlm.nih.gov/compound/Fluvastatin-sodium#section=Stability-Shelf-Life.

[56] Stevens J.S., Newton L.K., Jaye C., Muryn C.A., Fischer D.A., Schroeder S.L.M. (2015). Proton Transfer, Hydrogen Bonding, and Disorder: Nitrogen Near-Edge X-ray Absorption Fine Structure and X-ray Photoelectron Spectroscopy of Bipyridine–Acid Salts and Co-crystals. Cryst. Growth Des..

[57] Pandit V., Gorantla R., Devi K., Pai R.S., Sarasija S. (2011). Preparation and Characterization of Pioglitazone Cyclodextrin Inclusion Complexes. J. Young Pharm..

[58] Kissi E.O., Khorami K., Rades T. (2019). Determination of stable co-amorphous drug–drug ratios from the eutectic behavior of crystalline physical mixtures. Pharmaceutics.

[59] Babu N.J., Nangia A. (2011). Solubility advantage of amorphous drugs and pharmaceutical cocrystals. Cryst. Growth Des..

[60] Alonzo D.E., Zhang G.G.Z., Zhou D., Gao Y., Taylor L.S. (2010). Understanding the behavior of amorphous pharmaceutical systems during dissolution. Pharm. Res..

[61] Brough C., Williams R.O. (2013). Amorphous solid dispersions and nano-crystal technologies for poorly water-soluble drug delivery. Int. J. Pharm..

[62] Guzmán H.R., Tawa M., Zhang Z., Ratanabanangkoon P., Shaw P., Gardner C.R., Chen H., Moreau J., Almarsson Ö., Remenar J.F. (2007). Combined Use of Crystalline Salt Forms and Precipitation Inhibitors to Improve Oral Absorption of Celecoxib from Solid Oral Formulations. J. Pharm. Sci..

[63] Huang Y., Dai W.-G. (2014). Fundamental aspects of solid dispersion technology for poorly soluble drugs. Acta Pharm. Sin. B.

[64] Vullendula S.K.A., Nair A.R., Yarlagadda D.L., Navya Sree K.S., Bhat K., Dengale S.J. (2022). Polymeric solid dispersion Vs co-amorphous technology: A critical comparison. J. Drug Deliv. Sci. Technol..

[65] Dengale S.J., Grohganz H., Rades T., Löbmann K. (2016). Recent advances in co-amorphous drug formulations. Adv. Drug Deliv. Rev..

[66] Jouyban A., Soltanpour S. (2010). Solubility of pioglitazone hydrochloride in binary and ternary mixtures of water, propylene glycol, and polyethylene glycols 200, 400, and 600 at 298.2 K. AAPS PharmSciTech.

[67] Satheeshkumar N., Shantikumar S., Srinivas R. (2014). Pioglitazone: A review of analytical methods. J. Pharm. Anal..

[68] Jouyban A., Soltanpour S. (2010). Solubility of pioglitazone hydrochloride in binary mixtures of polyethylene glycol 400 with ethanol, propylene glycol, N-methyl-2-pyrrolidone, and water at 25 °C. Chem. Pharm. Bull..

