# Supplementary material for: Diversity of Solid Forms Promoted by Ball Milling: Characterization and Intrinsic Dissolution Studies of Pioglitazone Hydrochloride and Fluvastatin Sodium Drug–Drug Systems
Source: Pharmaceuticals (Basel). 2023 May 24;16(6):781. doi: 10.3390/ph16060781 (PMC10303448; doi:10.3390/ph16060781)
Supplement: Supplementary file 1 [file pharmaceuticals-16-00781-s001.zip › pharmaceuticals-2178074-supplementary.pdf]

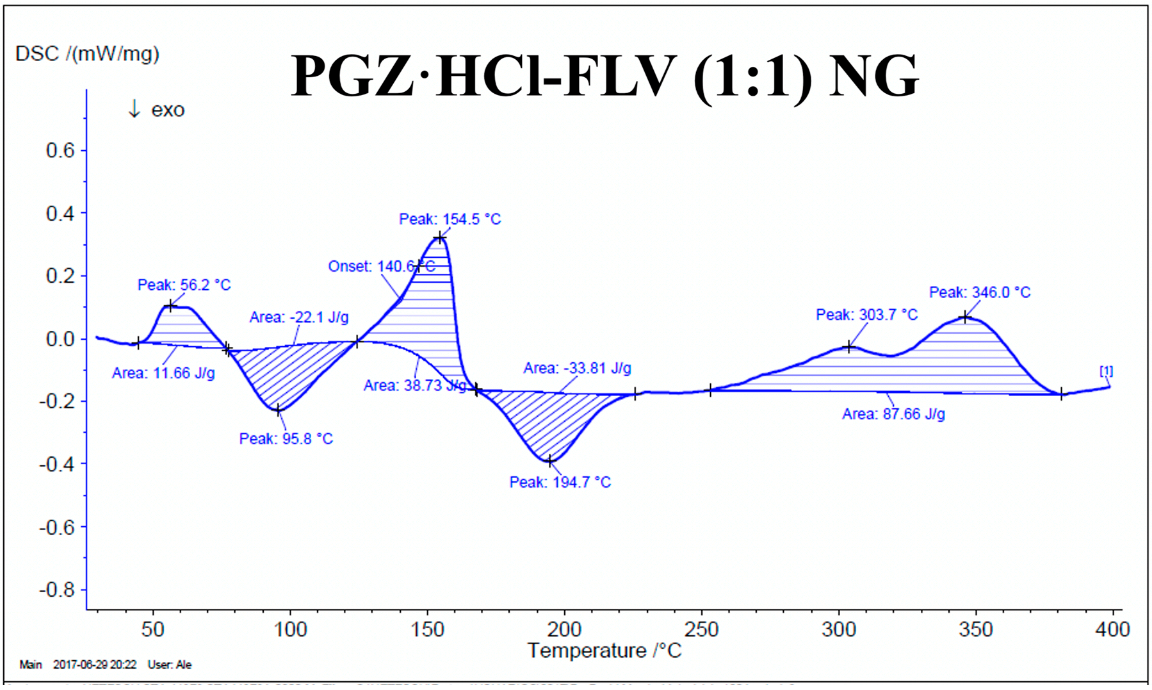
PGZ·HCl-FLV (1:1) NG
DSC /(mW/mg)
↓ exo
0.6
0.4
0.2
0.0
-0.2
-0.4
-0.6
-0.8
Peak: 56.2 °C
Area: 11.66 J/g
Area: -22.1 J/g
Peak: 95.8 °C
Onset: 140.6 °C
Peak: 154.5 °C
Area: 38.73 J/g
Area: -33.81 J/g
Peak: 194.7 °C
Peak: 303.7 °C
Peak: 346.0 °C
Area: 87.66 J/g
[1]
50
100
150
200
250
300
350
400
Temperature /°C
Main 2017-06-29 20:22 User: Ale

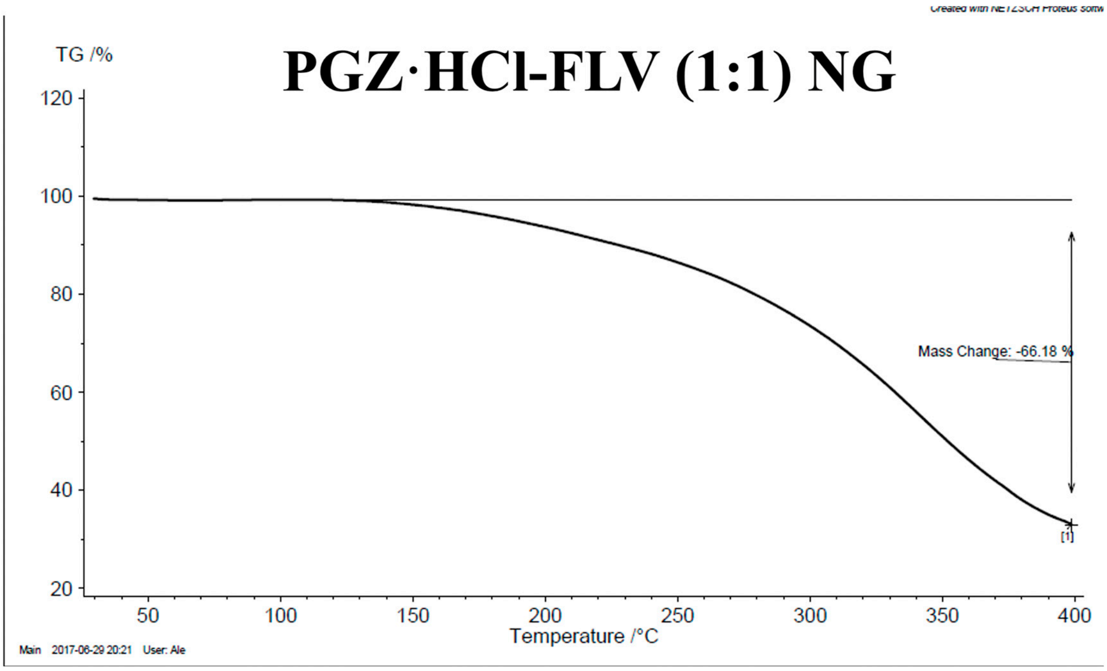
PGZ·HCl-FLV (1:1) NG
TG /%
120
100
80
60
40
20
Mass Change: -66.18 %
[1]
50
100
150
200
250
300
350
400
Temperature /°C
Main 2017-06-29 20:21 User: Ale

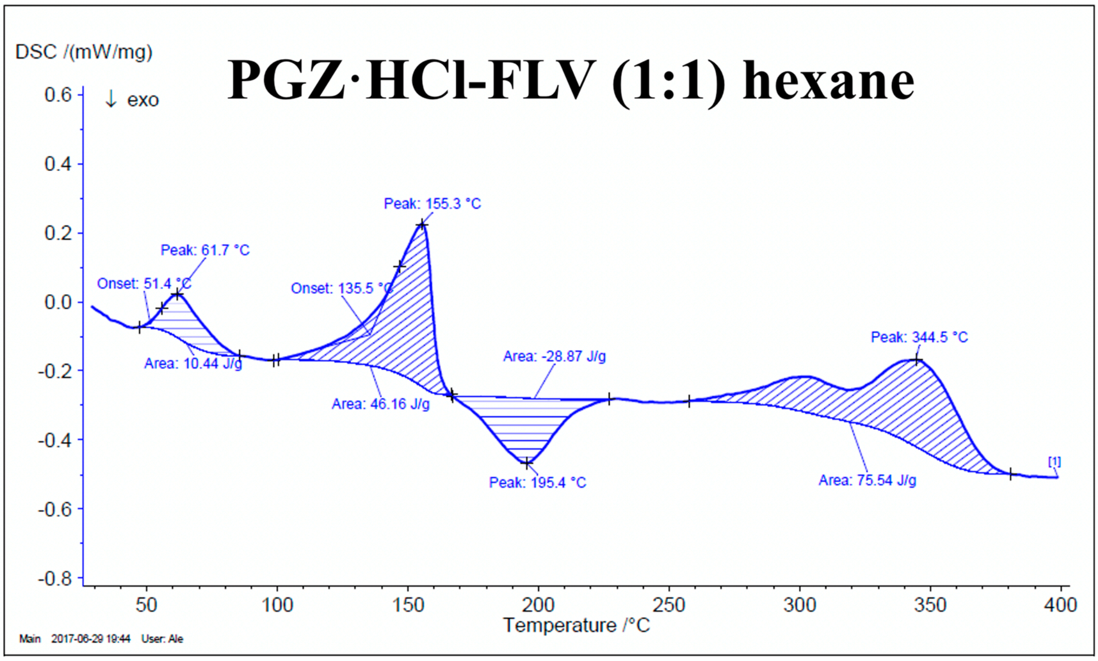

PGZ·HCl-FLV (1:1) hexane
DSC /(mW/mg)
↓ exo
Peak: 61.7 °C
Onset: 51.4 °C
Area: 10.44 J/g
Peak: 155.3 °C
Onset: 135.5 °C
Area: 46.16 J/g
Area: -28.87 J/g
Peak: 195.4 °C
Peak: 344.5 °C
Area: 75.54 J/g
[1]
Temperature /°C
Main 2017-06-29 19:44 User: Ale

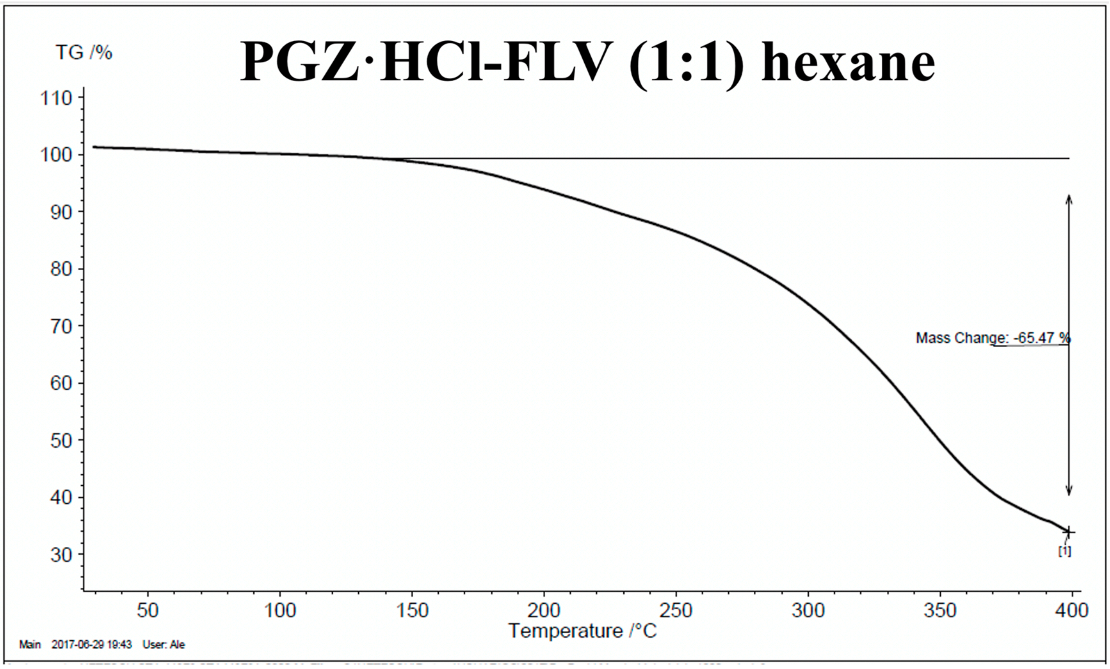

PGZ·HCl-FLV (1:1) hexane
TG /%
Mass Change: -65.47 %
[1]
Temperature /°C
Main 2017-06-29 19:43 User: Ale

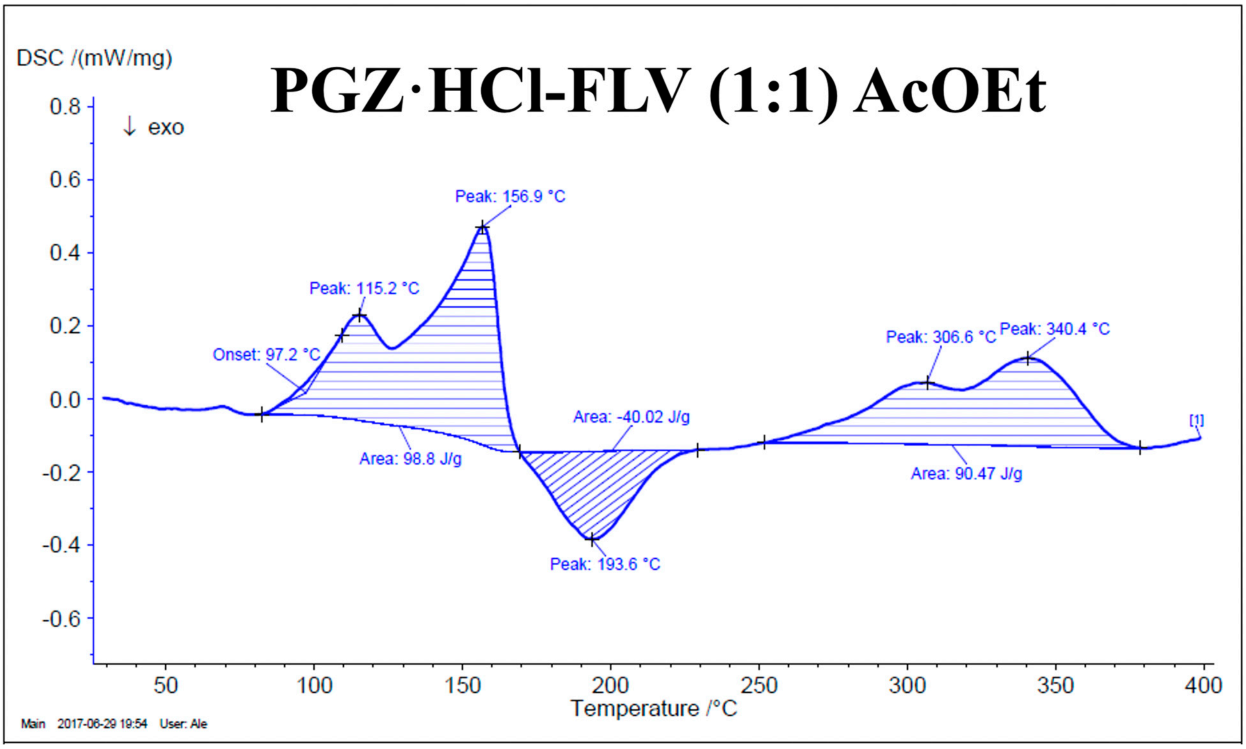
PGZ·HCl-FLV (1:1) AcOEt
DSC /(mW/mg)
↓ exo
0.8
0.6
0.4
0.2
0.0
-0.2
-0.4
-0.6
Peak: 156.9 °C
Peak: 115.2 °C
Onset: 97.2 °C
Area: 98.8 J/g
Area: -40.02 J/g
Peak: 193.6 °C
Peak: 306.6 °C
Peak: 340.4 °C
Area: 90.47 J/g
[1]
50
100
150
200
250
300
350
400
Temperature /°C
Main 2017-06-29 19:54 User: Ale

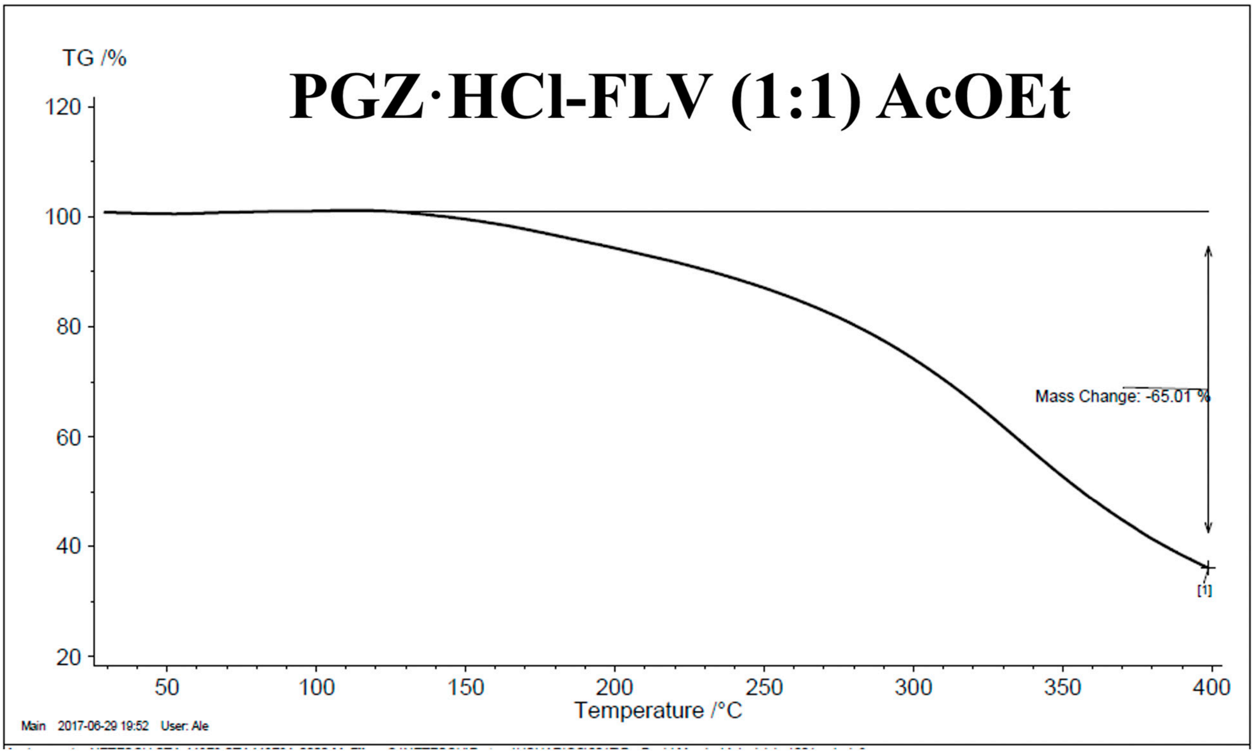
PGZ·HCl-FLV (1:1) AcOEt
TG /%
120
100
80
60
40
20
Mass Change: -65.01 %
[1]
50
100
150
200
250
300
350
400
Temperature /°C
Main 2017-06-29 19:52 User: Ale

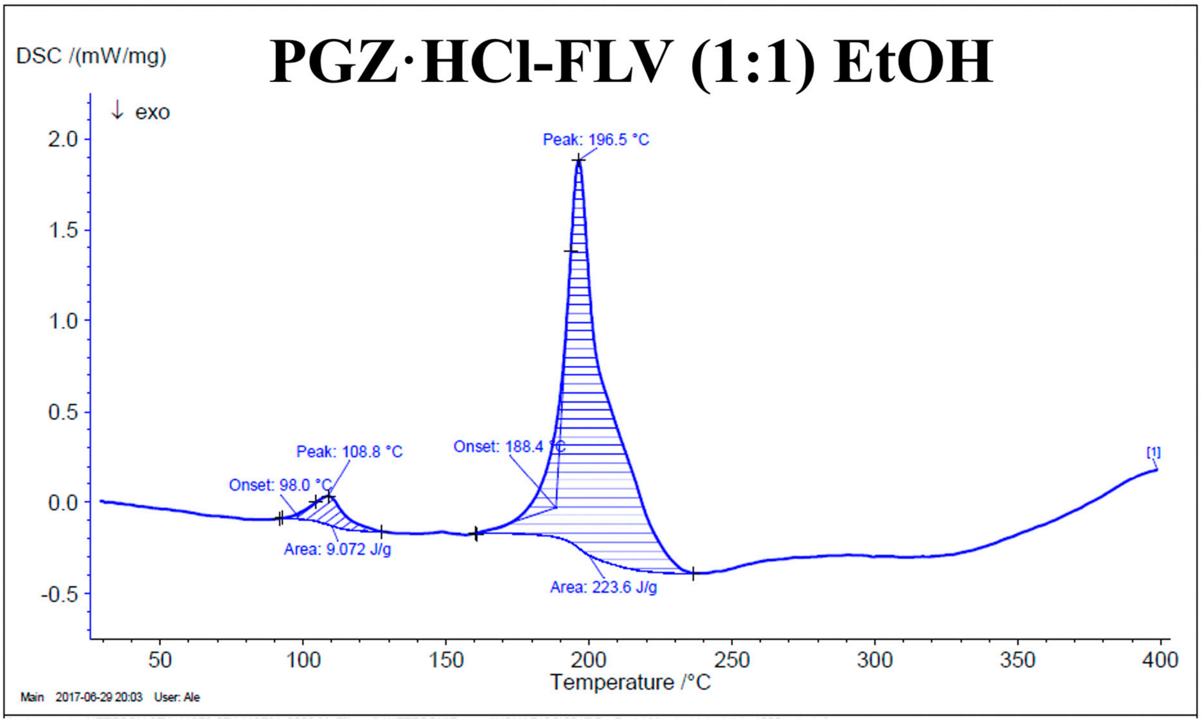
PGZ·HCl-FLV (1:1) EtOH
DSC /(mW/mg)
↓ exo
Peak: 196.5 °C
Peak: 108.8 °C
Onset: 188.4 °C
Onset: 98.0 °C
Area: 9.072 J/g
Area: 223.6 J/g
[1]
Temperature /°C
Main 2017-06-29 20:03 User: Ale

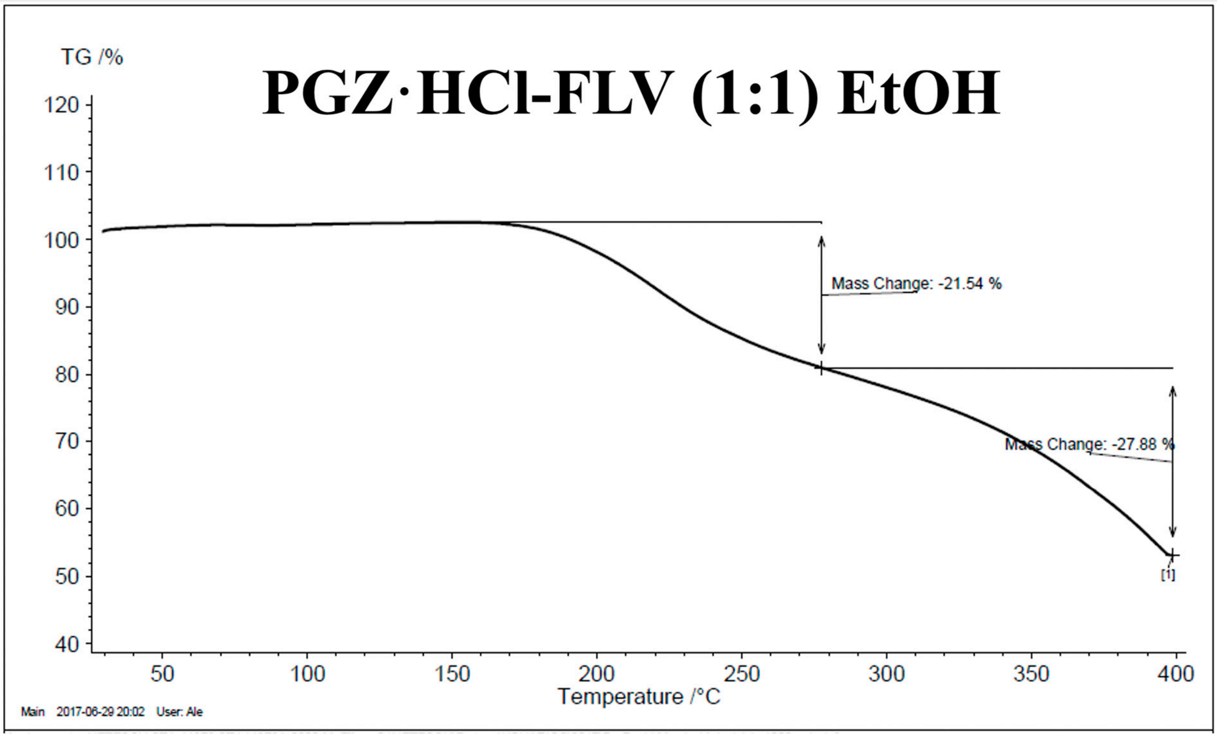
PGZ·HCl-FLV (1:1) EtOH
TG /%
Mass Change: -21.54 %
Mass Change: -27.88 %
[1]
Temperature /°C
Main 2017-06-29 20:02 User: Ale

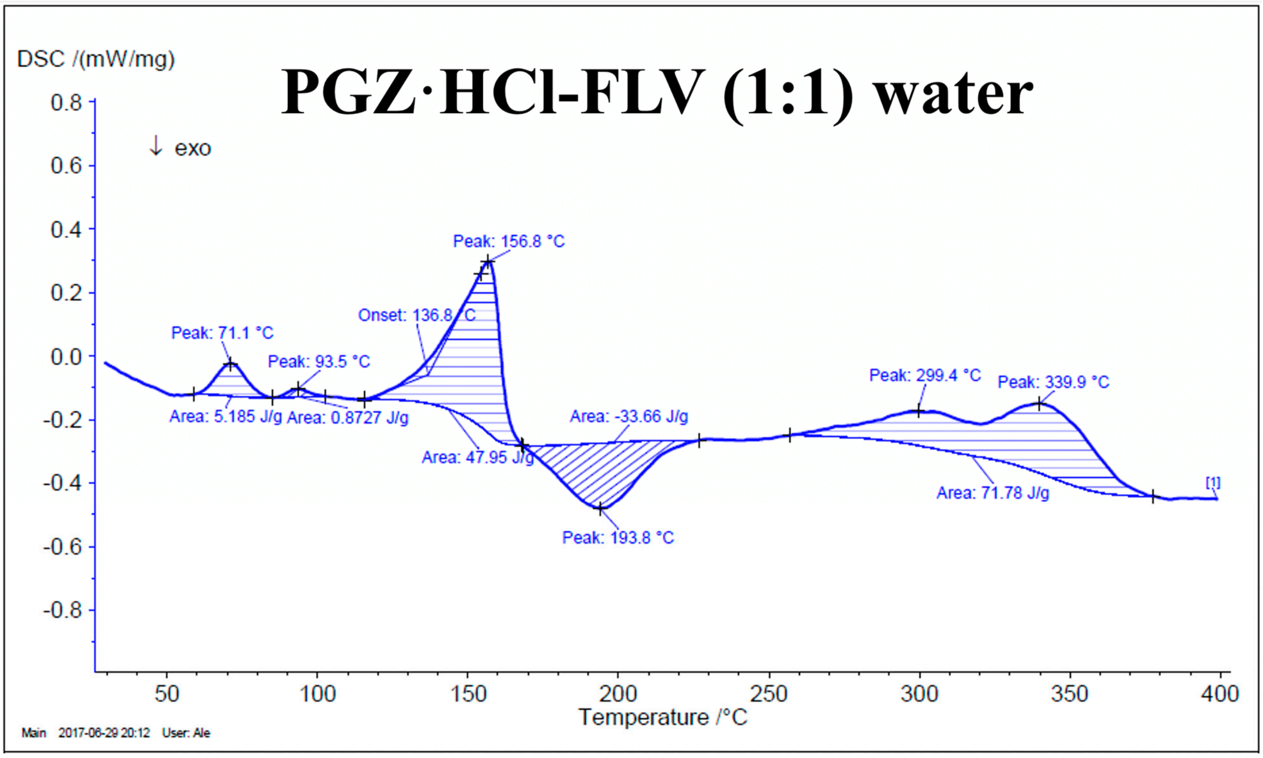
PGZ·HCl-FLV (1:1) water
DSC /(mW/mg)
↓ exo
Peak: 156.8 °C
Onset: 136.8 °C
Peak: 71.1 °C
Peak: 93.5 °C
Area: 5.185 J/g
Area: 0.8727 J/g
Area: 47.95 J/g
Area: -33.66 J/g
Peak: 193.8 °C
Peak: 299.4 °C
Peak: 339.9 °C
Area: 71.78 J/g
[1]
Temperature /°C
Main 2017-06-29 20:12 User: Ale

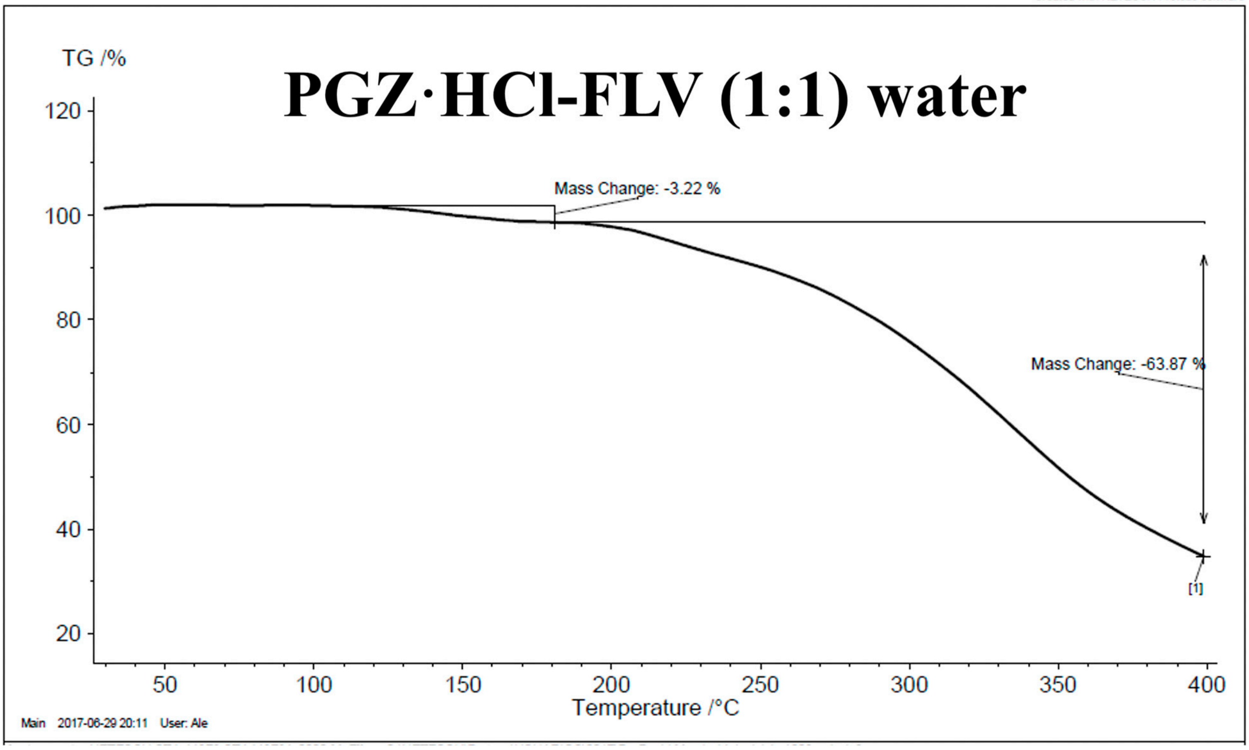
PGZ·HCl-FLV (1:1) water
TG /%
Mass Change: -3.22 %
Mass Change: -63.87 %
[1]
Temperature /°C
Main 2017-06-29 20:11 User: Ale

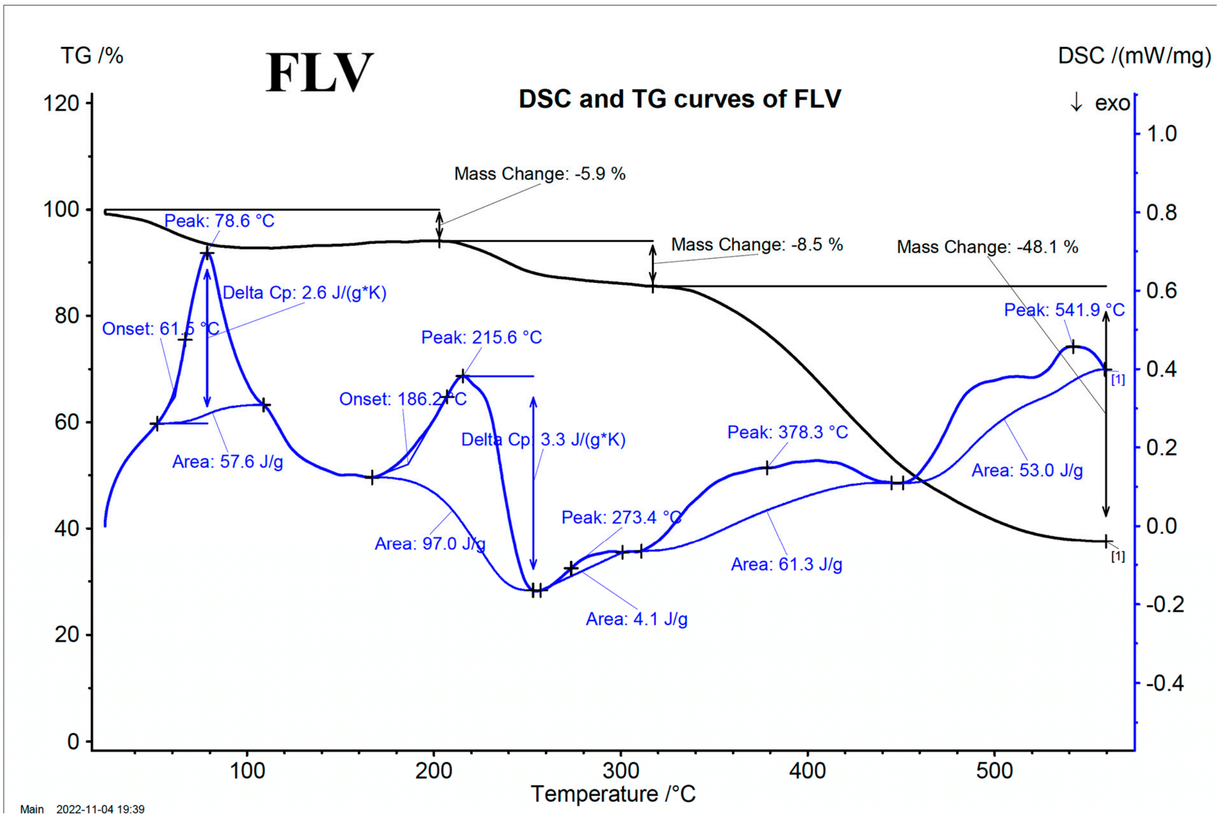
FLV
DSC and TG curves of FLV
TG /%
DSC /(mW/mg)
↓ exo
Mass Change: -5.9 %
Mass Change: -8.5 %
Mass Change: -48.1 %
Peak: 78.6 °C
Delta Cp: 2.6 J/(g*K)
Onset: 61.5 °C
Area: 57.6 J/g
Peak: 215.6 °C
Onset: 186.2 °C
Delta Cp: 3.3 J/(g*K)
Area: 97.0 J/g
Peak: 273.4 °C
Area: 4.1 J/g
Peak: 378.3 °C
Area: 61.3 J/g
Peak: 541.9 °C
Area: 53.0 J/g
[1]
Temperature /°C
Main 2022-11-04 19:39

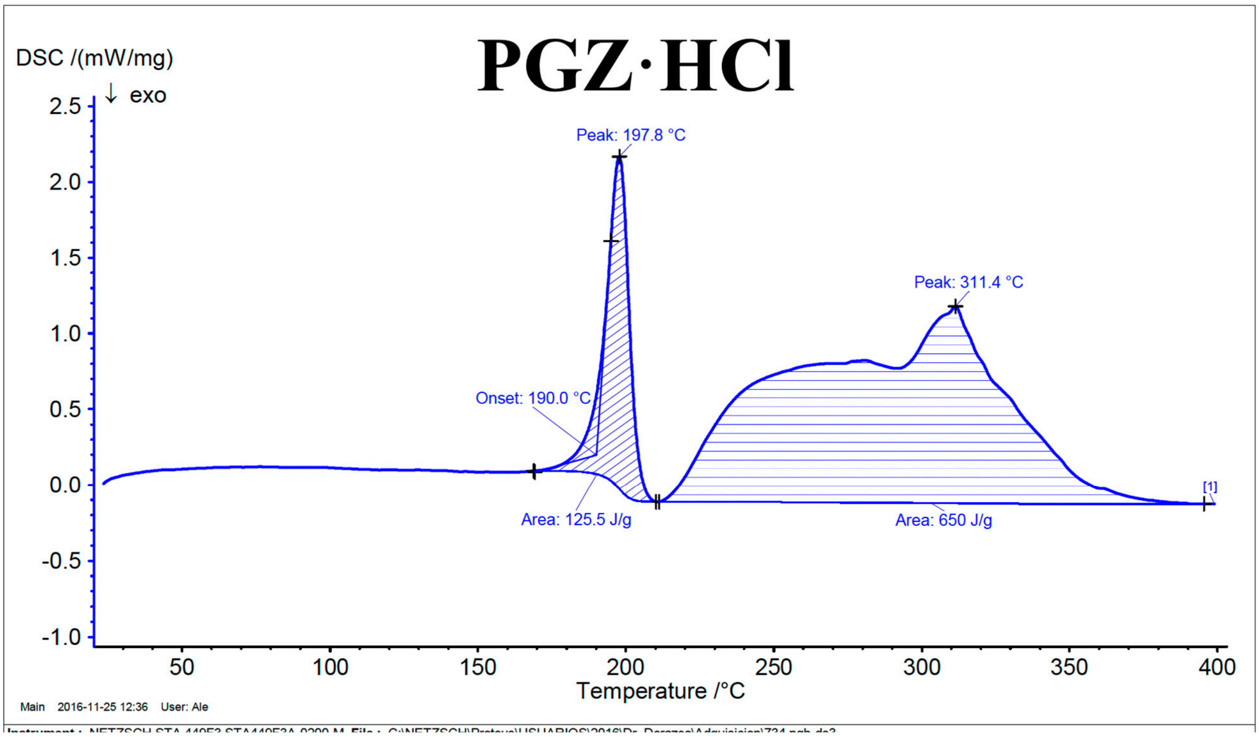
PGZ·HCl
DSC /(mW/mg)
↓ exo
Peak: 197.8 °C
Onset: 190.0 °C
Area: 125.5 J/g
Peak: 311.4 °C
Area: 650 J/g
[1]
Temperature /°C
Main 2016-11-25 12:36 User: Ale

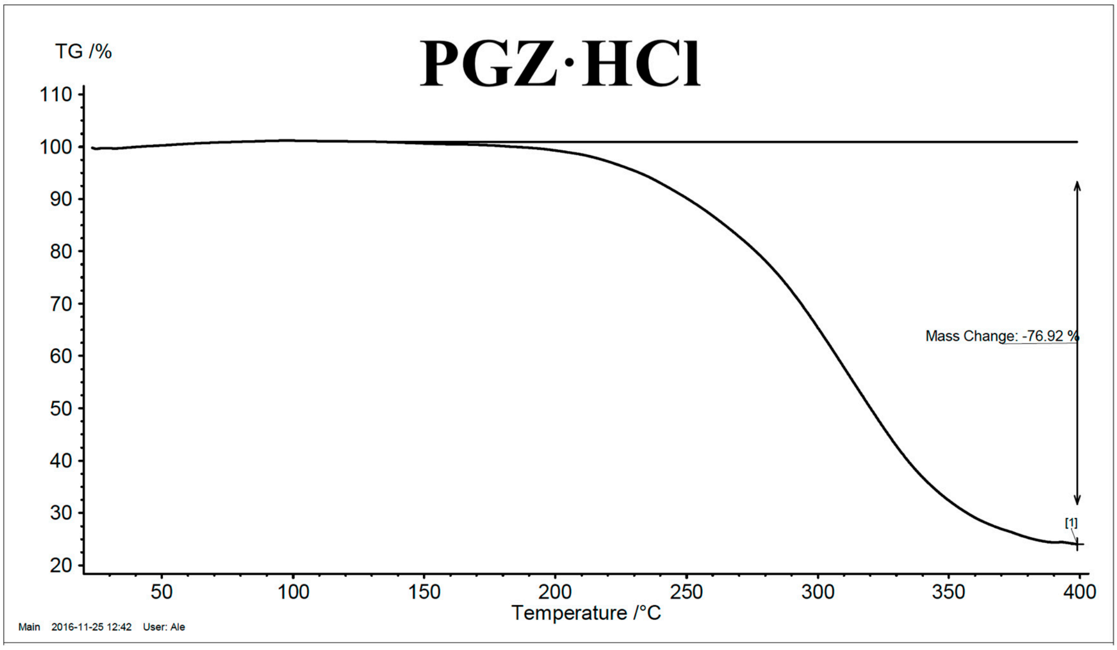

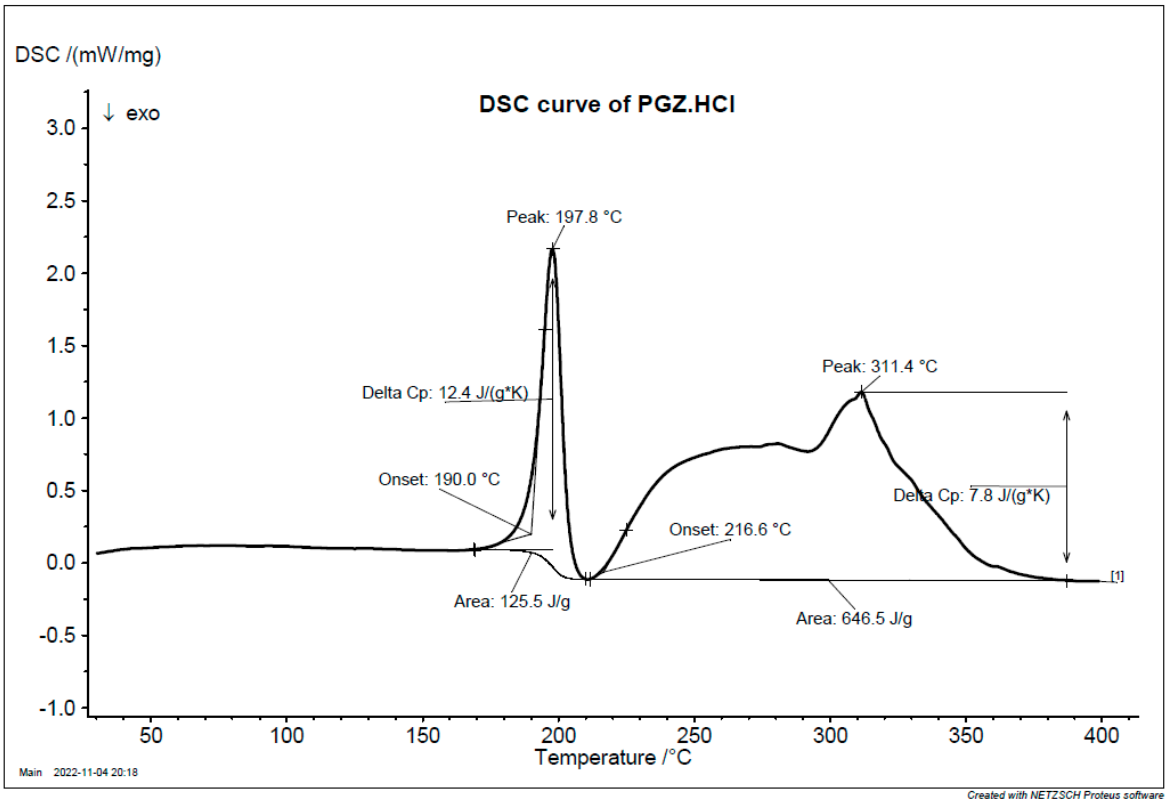

Figure SM1. Individual DSC-TGA thermograms of the solid phases PGZ·HCl-FLV (1:1) prepared by NG or LAG solvent-screening. Also, individual DSC-TGA thermograms of pure PGZ·HCl and FLV.

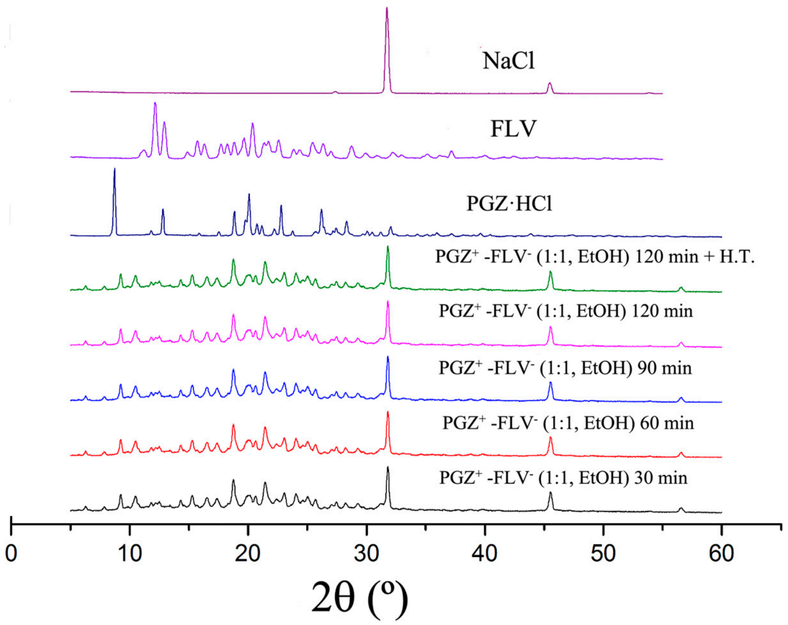

Figure SM2. Diffractograms of the salt PGZ^{+} -FLV^{-} (1:1, EtOH) extending the reaction times

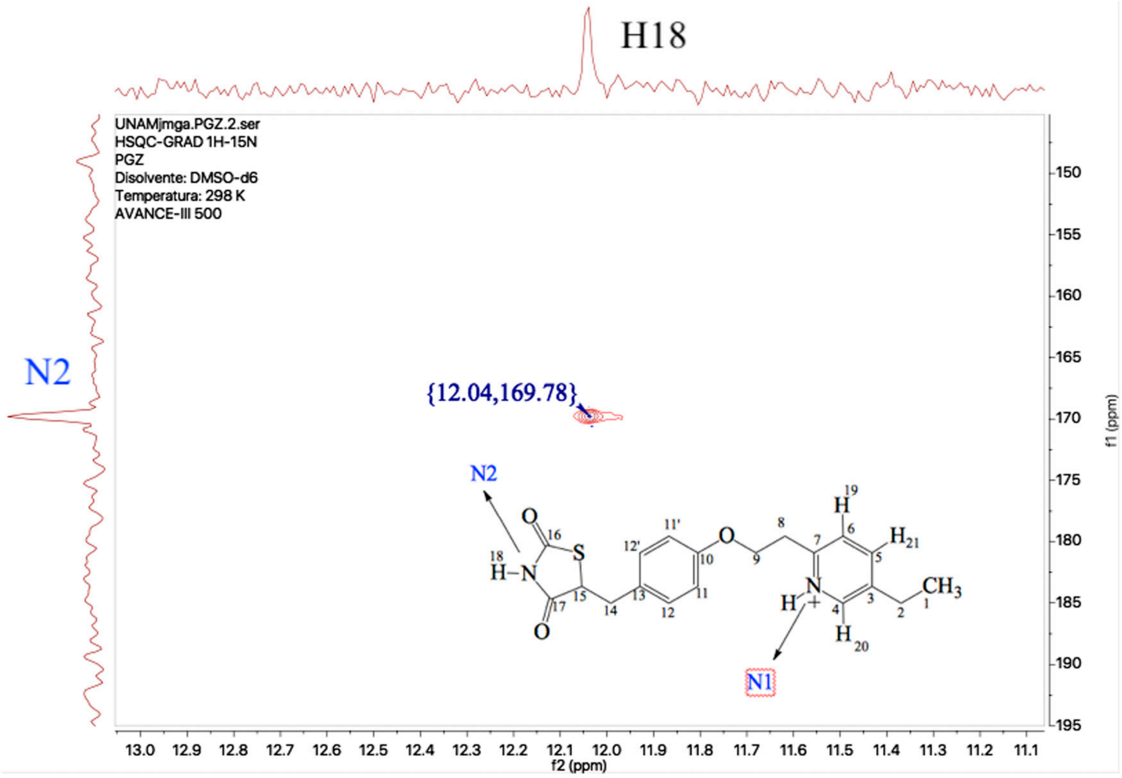

Figure SM3. HSQC ^{1}H-^{15}N NMR spectrum of PGZ·HCl in d_6-DMSO

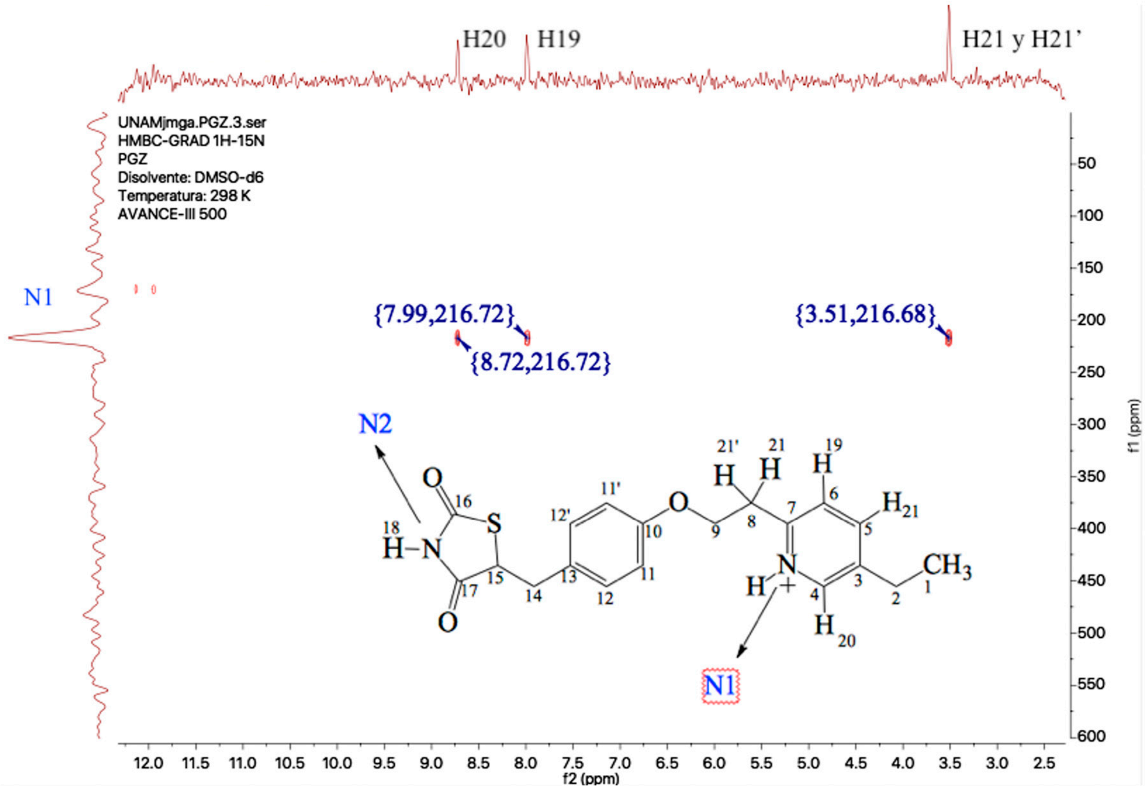

Figure SM4. HMBC ^{1}H-^{15}N NMR spectrum of PGZ·HCl in d_6-DMSO

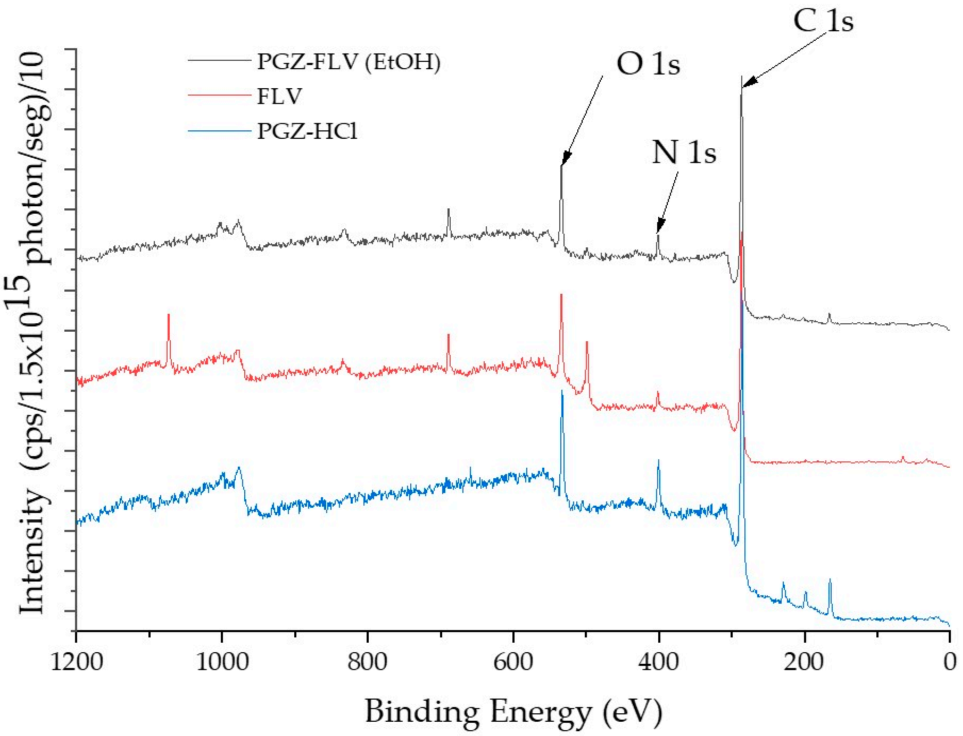

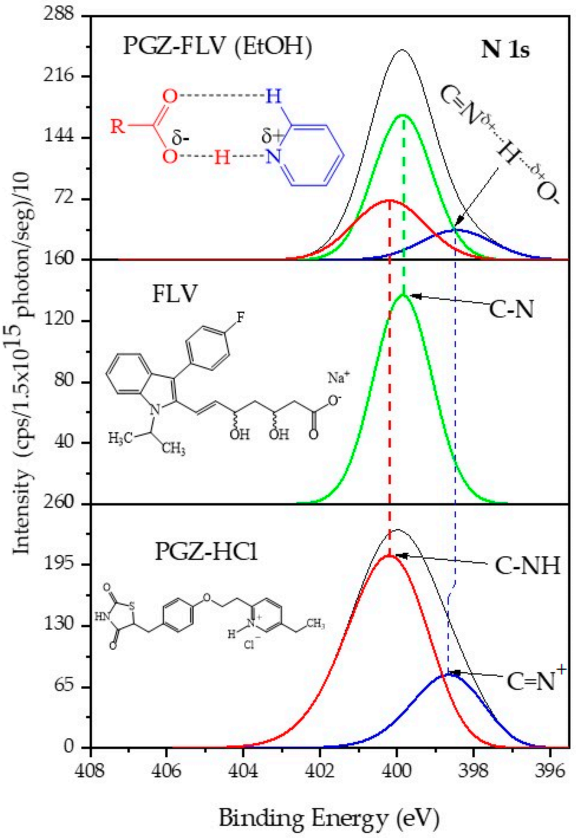

Figure SM5. XPS spectra of PGZHCl, FLV and the binary solid form (EtOH)

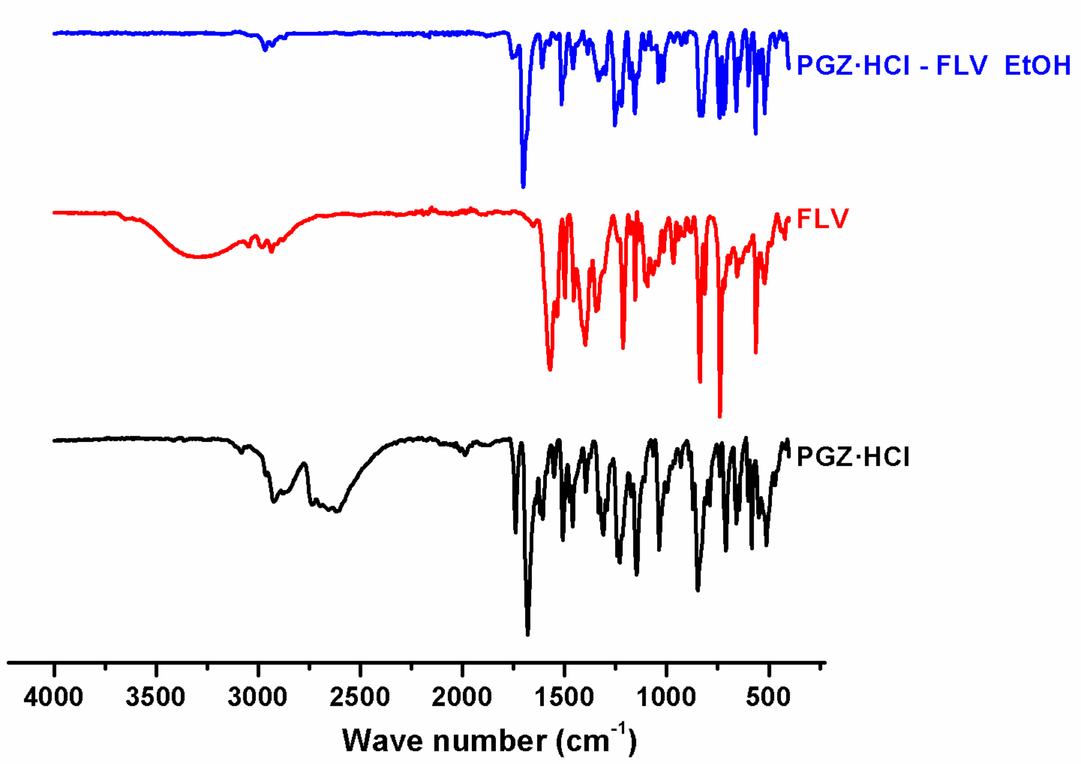

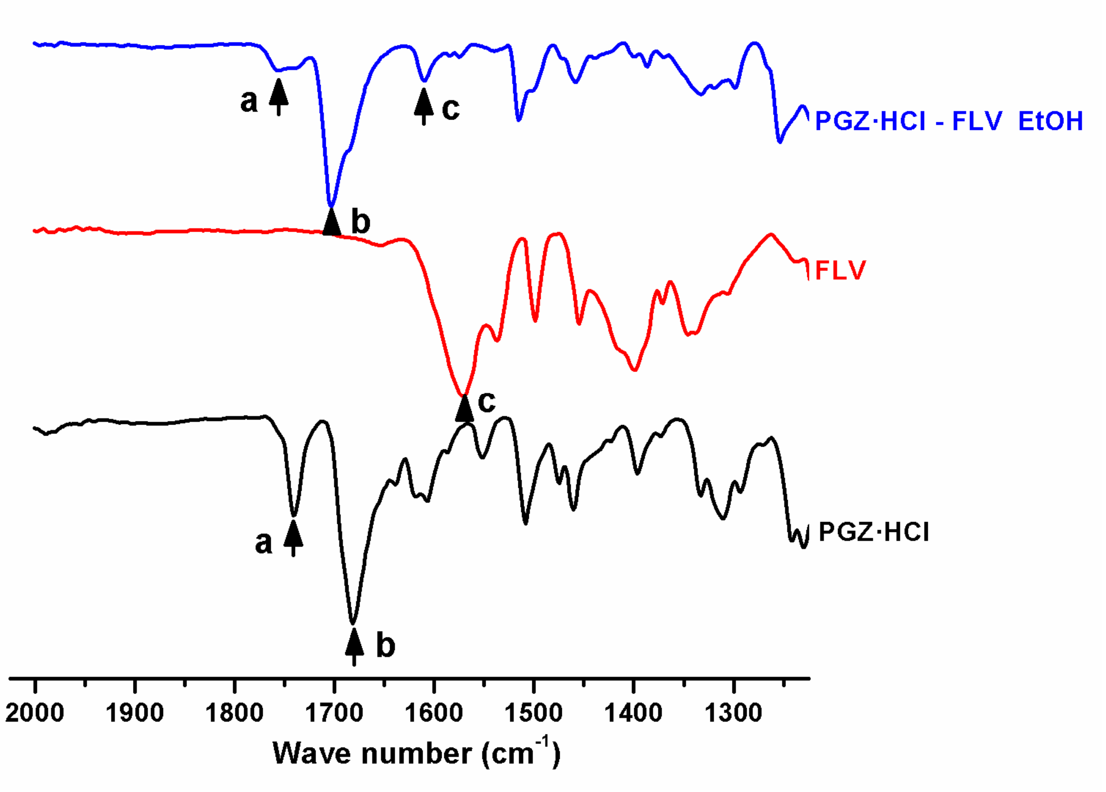

Vibrational band assignment	PGZ·HCl	FLV	PGZ·HCl-FLV (EtOH)
-C=O$_{PGZ}$ (a,b) (Δν cm^{-1})	a: 1738<br>b: 1681		a: 1756 (18)<br>b: 1702 (21)
-C=O$_{FLV}$ (c) (Δν cm^{-1})		c: 1571	c: 1610 (39)

∣Δν cm^{-1}∣ = vibration mode of pure drug - vibration mode of solid binary form

Figure SM6. Full and extended PGZHCl, FLV, and PGZ-FLV (EtOH) FTIR spectra. Likewise, the table indicates the frequency values.

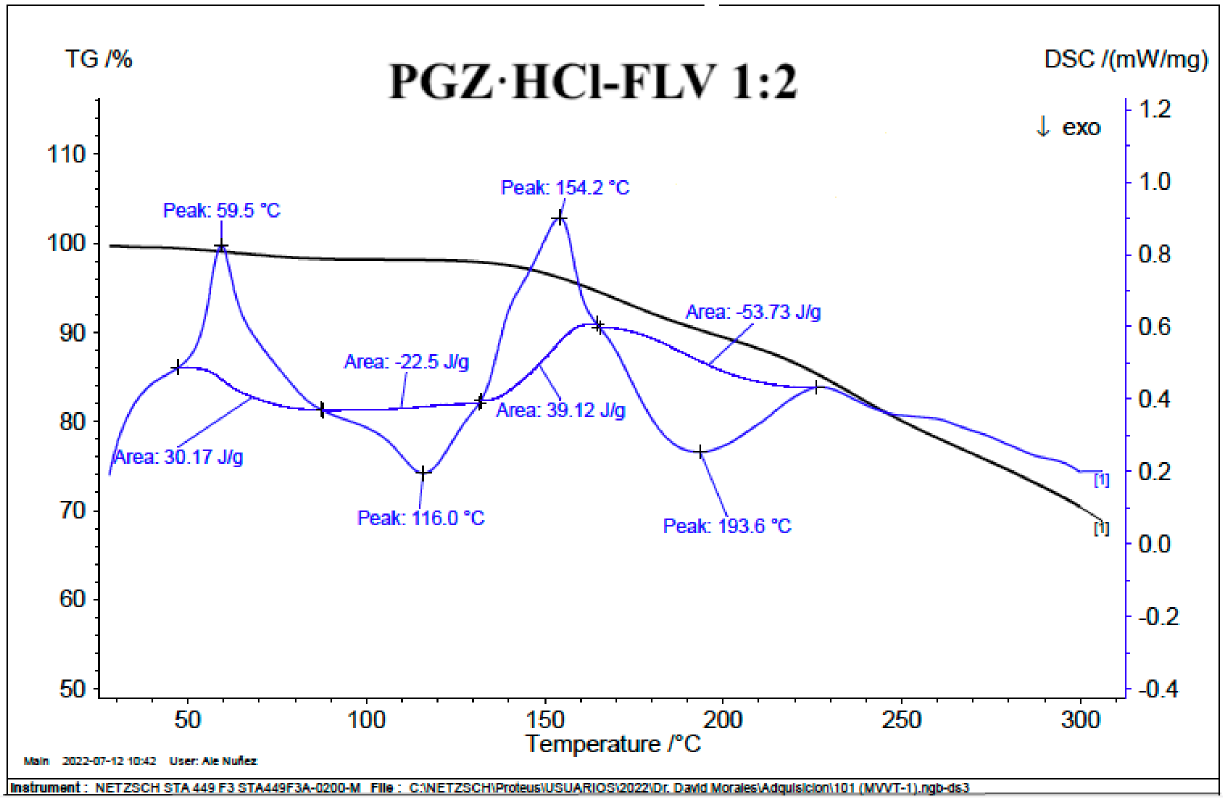
PGZ·HCl-FLV 1:2
TG /%
DSC /(mW/mg)
↓ exo
Peak: 59.5 °C
Peak: 154.2 °C
Area: -53.73 J/g
Area: -22.5 J/g
Area: 39.12 J/g
Area: 30.17 J/g
Peak: 116.0 °C
Peak: 193.6 °C
Temperature /°C
Main 2022-07-12 10:42 User: Ale Nuñez
Instrument : NETZSCH STA 449 F3 STA449F3A-0200-M File : C:\NETZSCH\Proteus\USUARIOS\2022\Dr. David Morales\Adquisicion\101 (MVVT-1).ngb-ds3

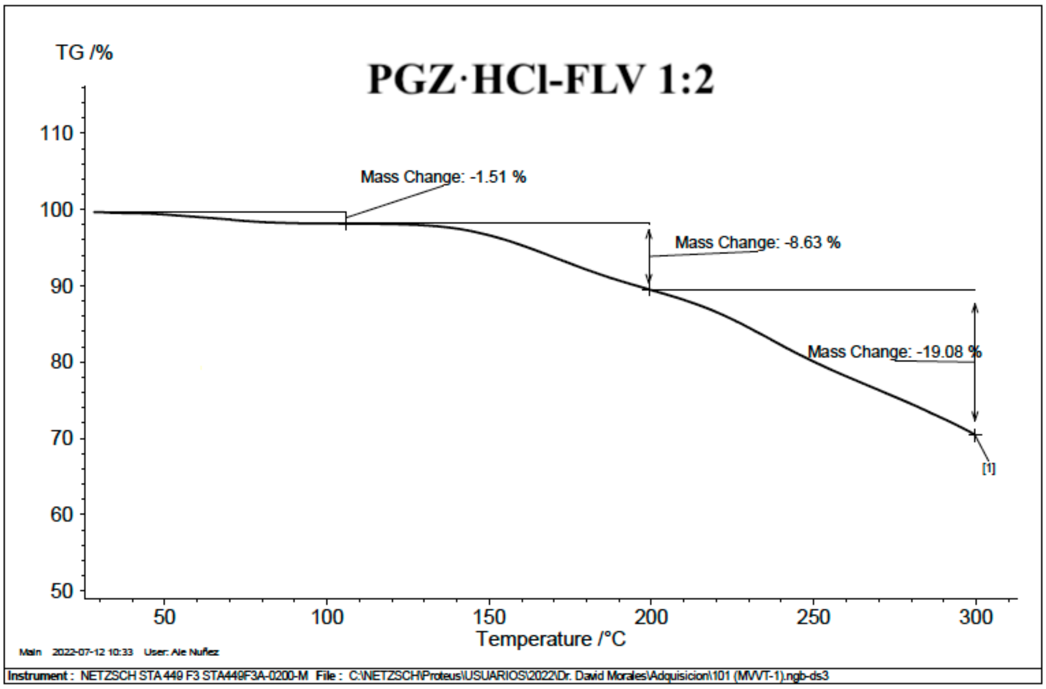
PGZ·HCl-FLV 1:2
TG /%
Mass Change: -1.51 %
Mass Change: -8.63 %
Mass Change: -19.08 %
Temperature /°C
Main 2022-07-12 10:33 User: Ale Nuñez
Instrument : NETZSCH STA 449 F3 STA449F3A-0200-M File : C:\NETZSCH\Proteus\USUARIOS\2022\Dr. David Morales\Adquisicion\101 (MVVT-1).ngb-ds3

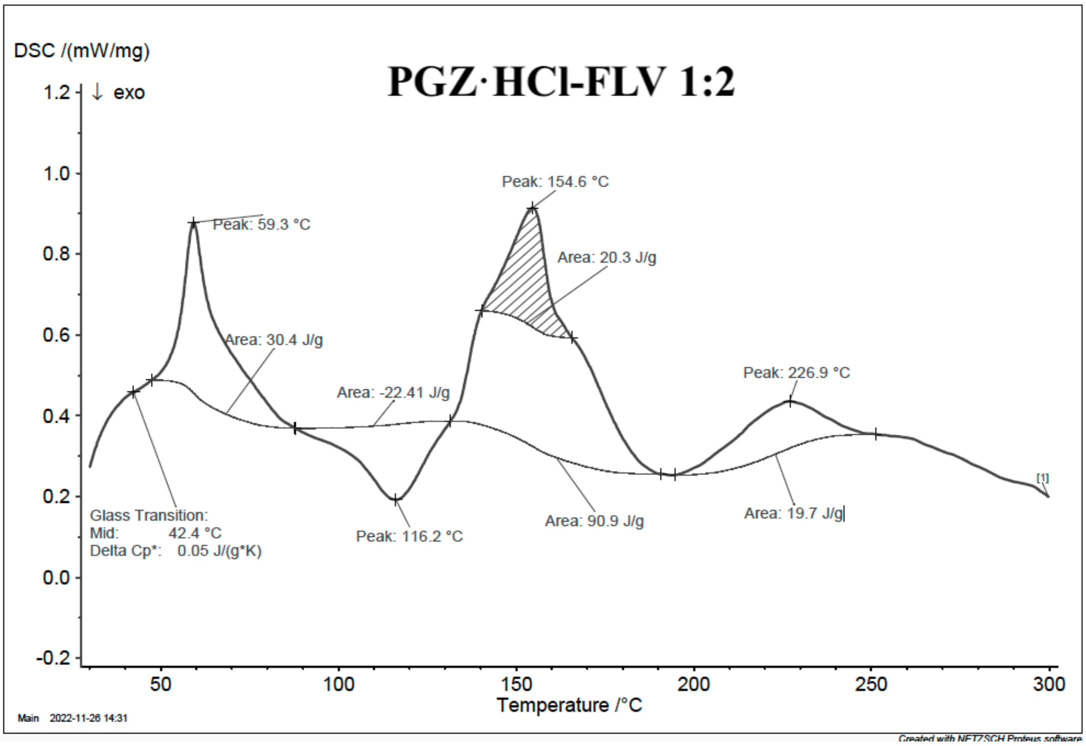
DSC /(mW/mg)
↓ exo
PGZ·HCl-FLV 1:2
Peak: 59.3 °C
Peak: 154.6 °C
Area: 20.3 J/g
Area: 30.4 J/g
Area: -22.41 J/g
Peak: 226.9 °C
Glass Transition:
Mid: 42.4 °C
Delta Cp*: 0.05 J/(g*K)
Peak: 116.2 °C
Area: 90.9 J/g
Area: 19.7 J/g
Temperature /°C
Main 2022-11-26 14:31
Created with NETZSCH Proteus software

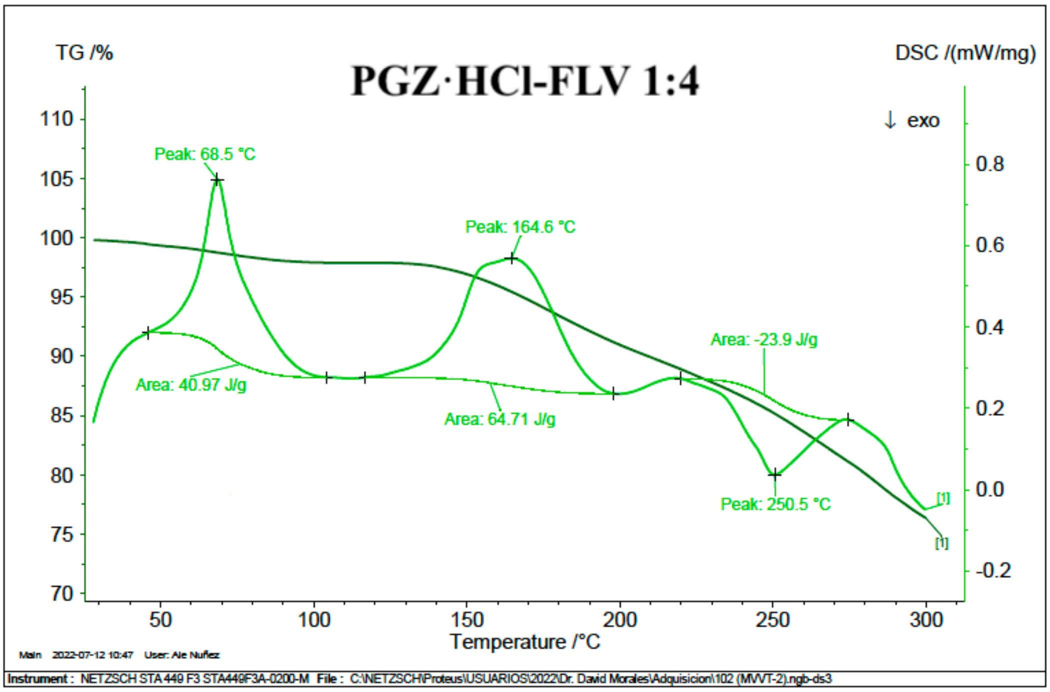
TG /%
DSC /(mW/mg)
PGZ·HCl-FLV 1:4
↓ exo
Peak: 68.5 °C
Peak: 164.6 °C
Area: -23.9 J/g
Area: 40.97 J/g
Area: 64.71 J/g
Peak: 250.5 °C
Temperature /°C
Main 2022-07-12 10:47 User: Ale Nuñez
Instrument : NETZSCH STA 449 F3 STA449F3A-0200-M File : C:\NETZSCH\Proteus\USUARIOS\2022\Dr. David Morales\Adquisicion\102 (MVVT-2).ngb-ds3

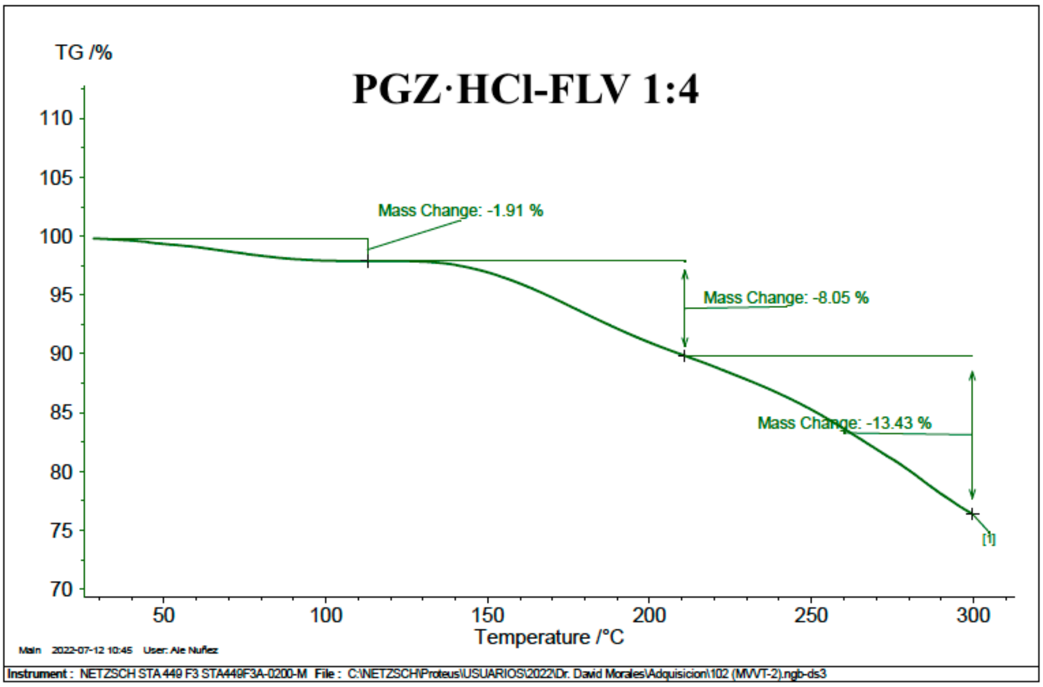
TG /%
PGZ·HCl-FLV 1:4
Mass Change: -1.91 %
Mass Change: -8.05 %
Mass Change: -13.43 %
Temperature /°C
Main 2022-07-12 10:45 User: Ale Nuñez
Instrument : NETZSCH STA 449 F3 STA449F3A-0200-M File : C:\NETZSCH\Proteus\USUARIOS\2022\Dr. David Morales\Adquisicion\102 (MVVT-2).ngb-ds3

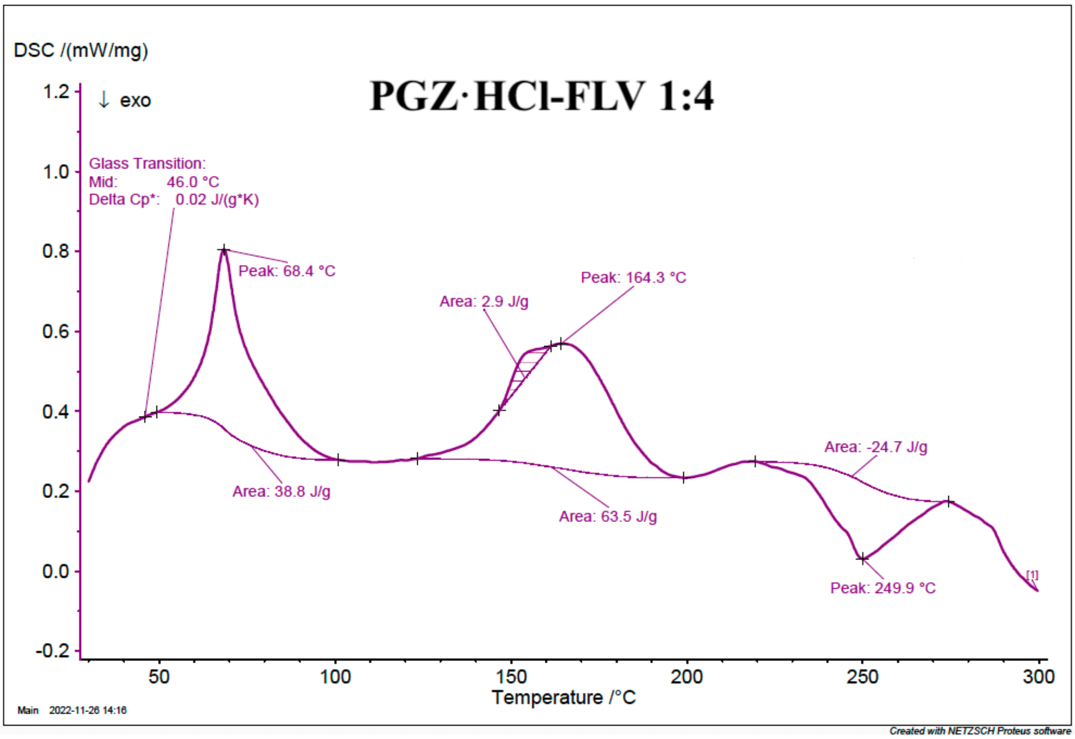
DSC /(mW/mg)
↓ exo
PGZ·HCl-FLV 1:4
Glass Transition:
Mid: 46.0 °C
Delta Cp*: 0.02 J/(g*K)
Peak: 68.4 °C
Area: 2.9 J/g
Peak: 164.3 °C
Area: 38.8 J/g
Area: 63.5 J/g
Area: -24.7 J/g
Peak: 249.9 °C
Temperature /°C
Main 2022-11-26 14:16
Created with NETZSCH Proteus software

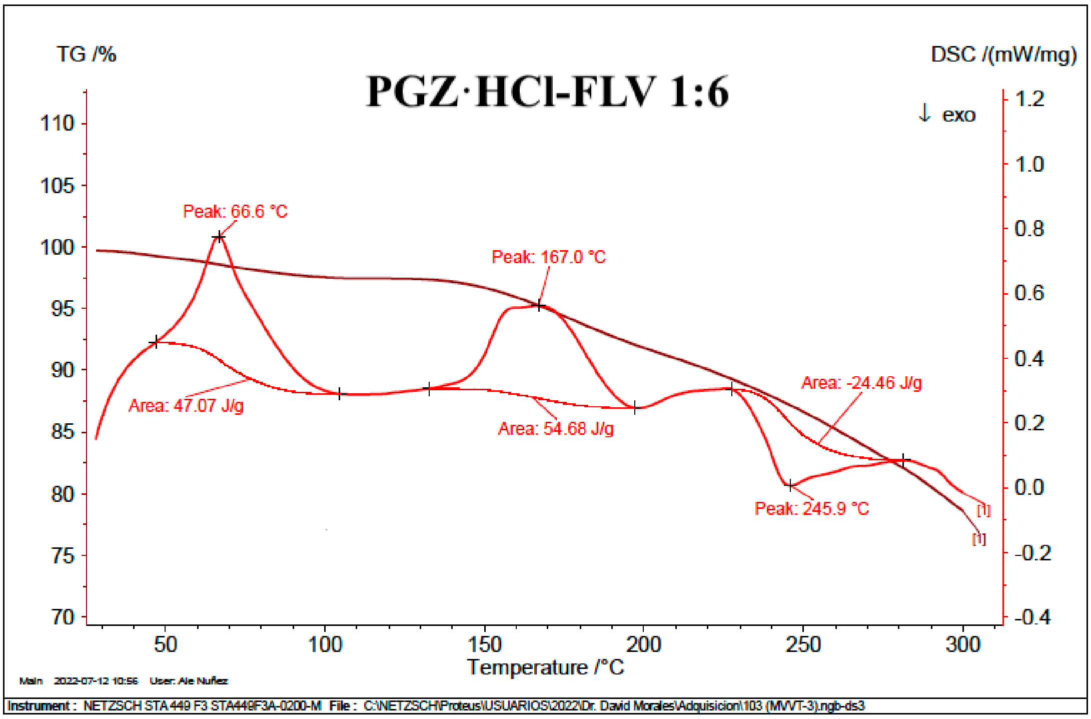
TG /%
DSC /(mW/mg)
PGZ·HCl-FLV 1:6
↓ exo
Peak: 66.6 °C
Peak: 167.0 °C
Area: 47.07 J/g
Area: 54.68 J/g
Area: -24.46 J/g
Peak: 245.9 °C
Temperature /°C
Main 2022-07-12 10:56 User: Ale Nuñez
Instrument : NETZSCH STA 449 F3 STA449F3A-0200-M File : C:\NETZSCH\Proteus\USUARIOS\2022\Dr. David Morales\Adquisicion\103 (MVVT-3).ngb-ds3

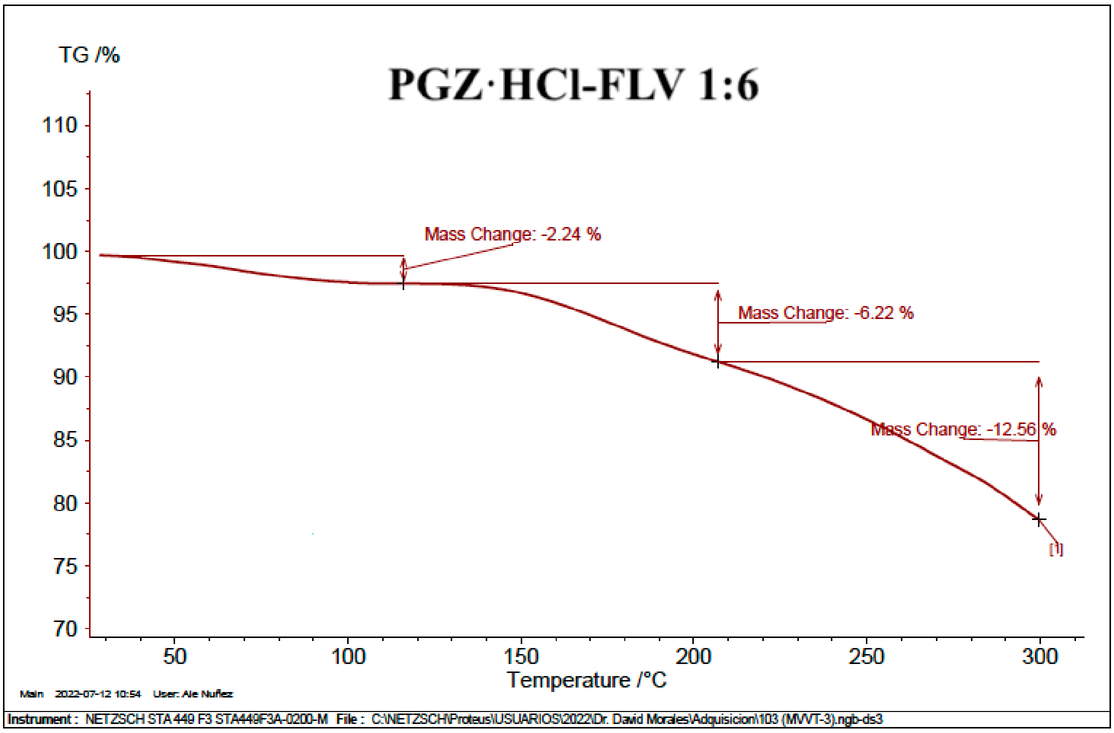
TG /%
PGZ·HCl-FLV 1:6
Mass Change: -2.24 %
Mass Change: -6.22 %
Mass Change: -12.56 %
Temperature /°C
Main 2022-07-12 10:54 User: Ale Nuñez
Instrument : NETZSCH STA 449 F3 STA449F3A-0200-M File : C:\NETZSCH\Proteus\USUARIOS\2022\Dr. David Morales\Adquisicion\103 (MVVT-3).ngb-ds3

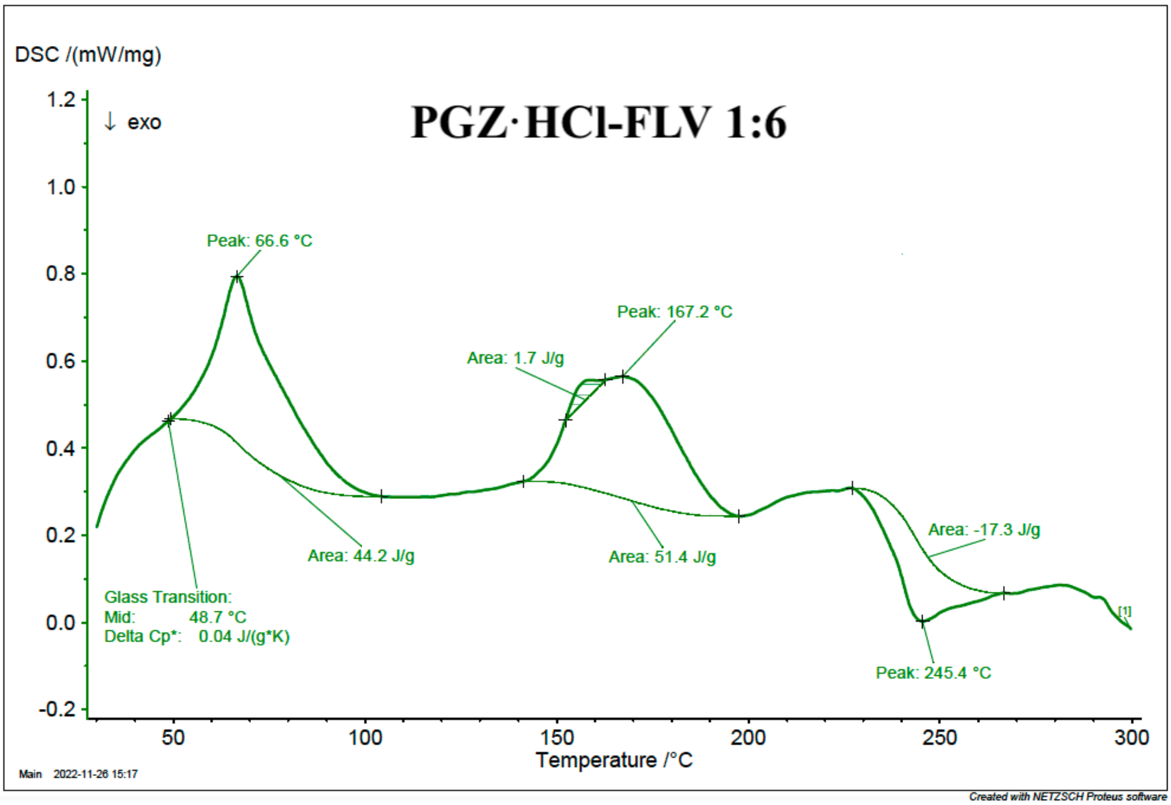
DSC /(mW/mg)
↓ exo
PGZ·HCl-FLV 1:6
Peak: 66.6 °C
Peak: 167.2 °C
Area: 1.7 J/g
Area: 44.2 J/g
Area: 51.4 J/g
Area: -17.3 J/g
Glass Transition:
Mid: 48.7 °C
Delta Cp*: 0.04 J/(g*K)
Peak: 245.4 °C
[1]
1.2
1.0
0.8
0.6
0.4
0.2
0.0
-0.2
50
100
150
200
250
300
Temperature /°C
Main 2022-11-26 15:17
Created with NETZSCH Proteus software

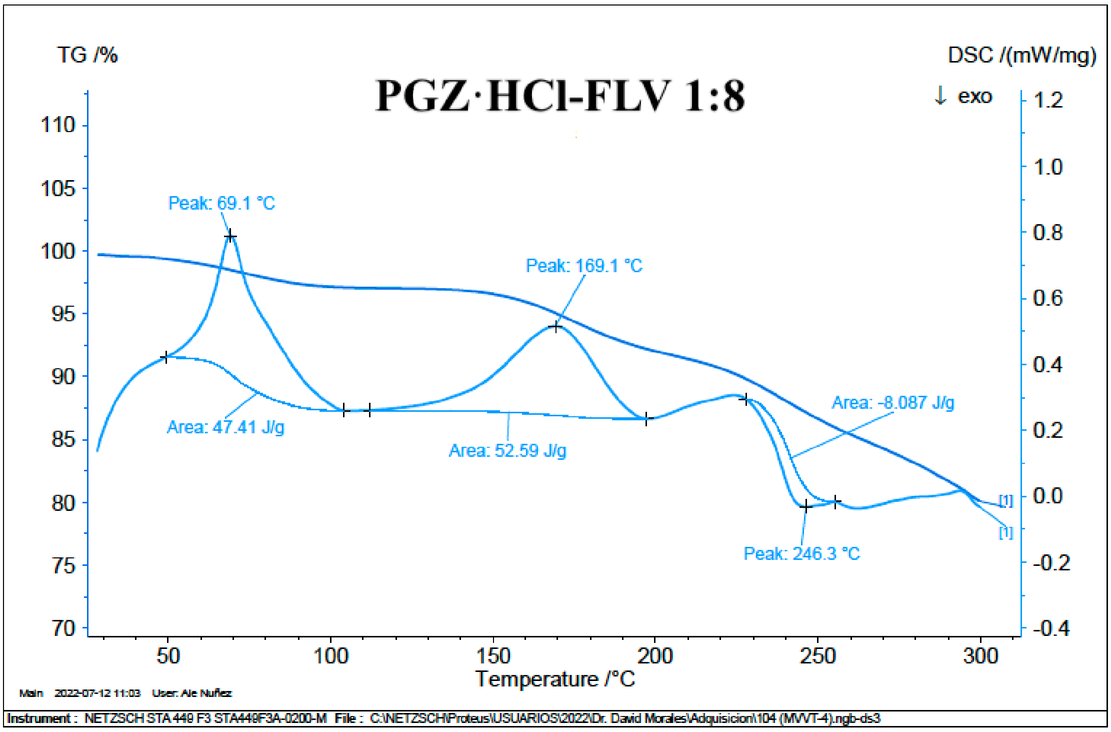
TG /%
DSC /(mW/mg)
PGZ·HCl-FLV 1:8
↓ exo
Peak: 69.1 °C
Peak: 169.1 °C
Area: 47.41 J/g
Area: 52.59 J/g
Area: -8.087 J/g
Peak: 246.3 °C
[1]
[1]
110
105
100
95
90
85
80
75
70
1.2
1.0
0.8
0.6
0.4
0.2
0.0
-0.2
-0.4
50
100
150
200
250
300
Temperature /°C
Main 2022-07-12 11:03 User: Ale Nuñez
Instrument : NETZSCH STA 449 F3 STA449F3A-0200-M File : C:\NETZSCH\Proteus\USUARIOS\2022\Dr. David Morales\Adquisicion\104 (MVT-4).ngb-ds3

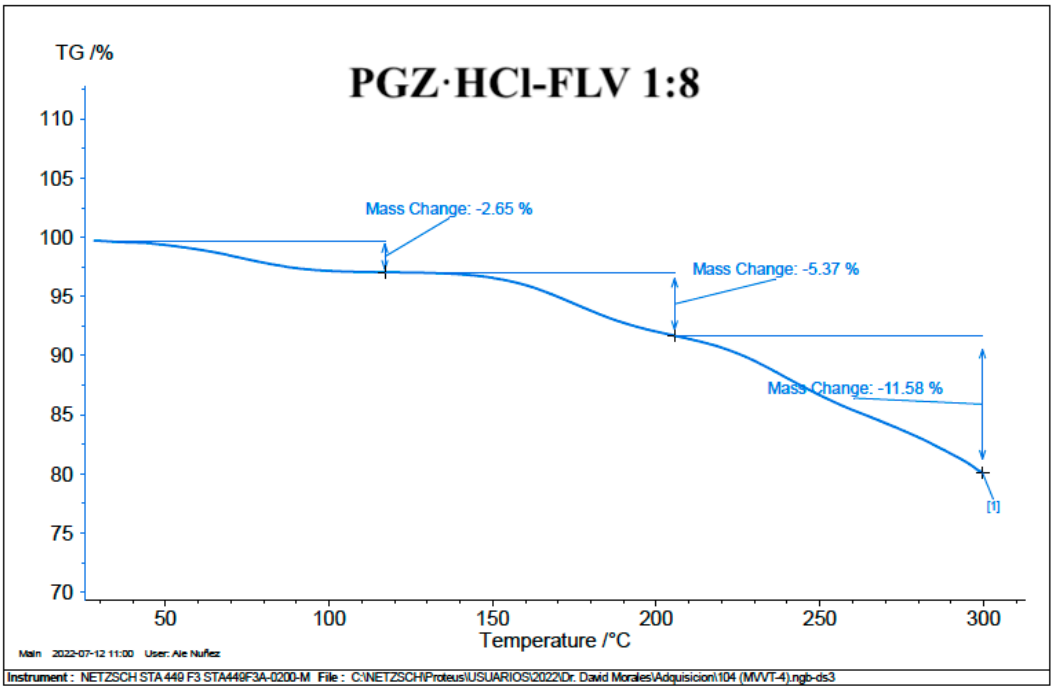
TG /%
PGZ·HCl-FLV 1:8
110
105
100
95
90
85
80
75
70
Mass Change: -2.65 %
Mass Change: -5.37 %
Mass Change: -11.58 %
[1]
50
100
150
200
250
300
Temperature /°C
Main 2022-07-12 11:00 User: Ale Nuñez
Instrument : NETZSCH STA 449 F3 STA449F3A-0200-M File : C:\NETZSCH\Proteus\USUARIOS\2022\Dr. David Morales\Adquisicion\104 (MVVT-4).ngb-ds3

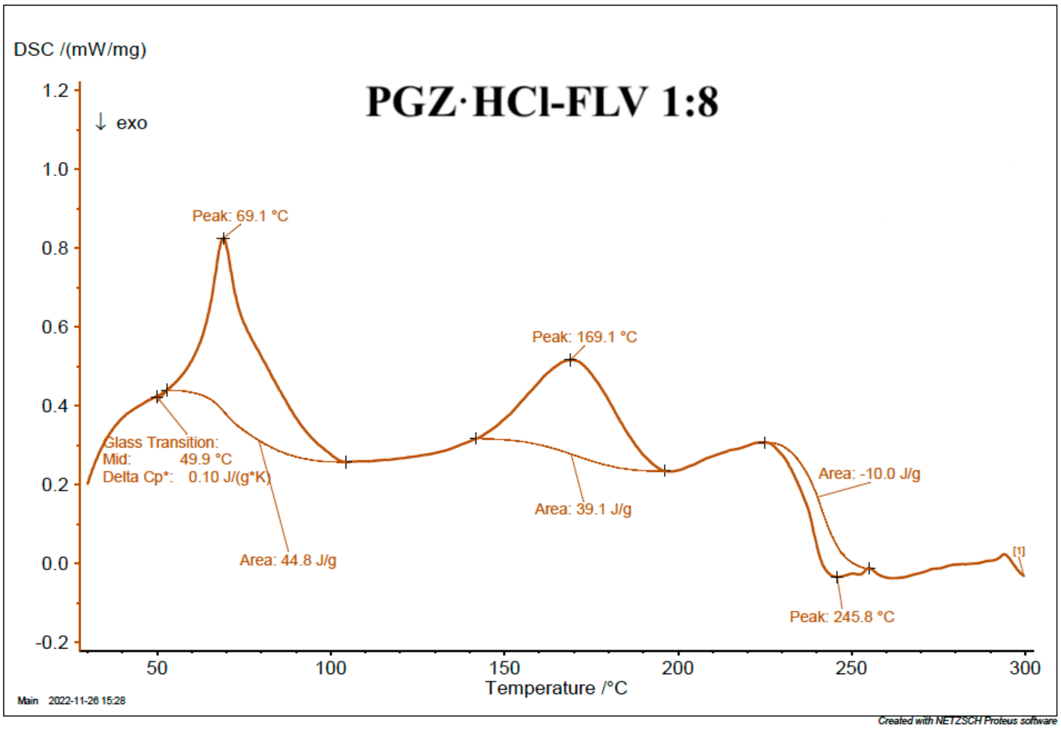
DSC /(mW/mg)
PGZ·HCl-FLV 1:8
↓ exo
1.2
1.0
0.8
0.6
0.4
0.2
0.0
-0.2
Peak: 69.1 °C
Peak: 169.1 °C
Glass Transition:
Mid: 49.9 °C
Delta Cp*: 0.10 J/(g*K)
Area: 44.8 J/g
Area: 39.1 J/g
Area: -10.0 J/g
Peak: 245.8 °C
[1]
50
100
150
200
250
300
Temperature /°C
Main 2022-11-26 15:28
Created with NETZSCH Proteus software

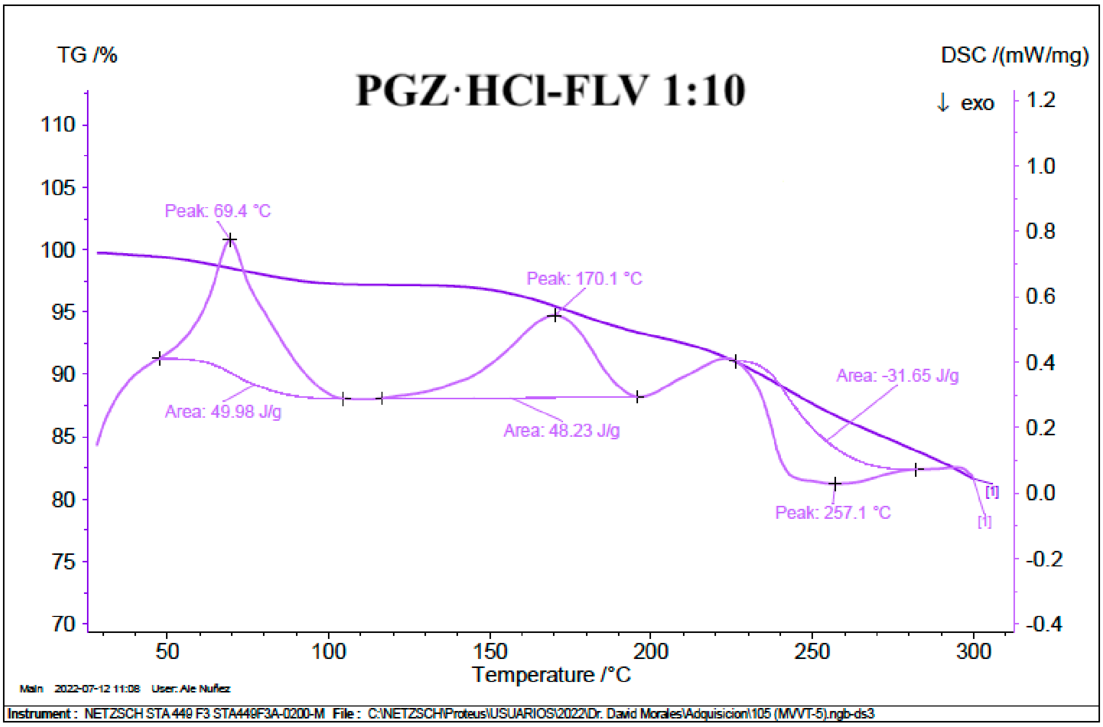
TG /%
PGZ·HCl-FLV 1:10
DSC /(mW/mg)
↓ exo
Peak: 69.4 °C
Peak: 170.1 °C
Area: 49.98 J/g
Area: 48.23 J/g
Area: -31.65 J/g
Peak: 257.1 °C
Temperature /°C
Main 2022-07-12 11:08 User: Ale Nuñez
Instrument : NETZSCH STA 449 F3 STA449F3A-0200-M File : C:\NETZSCH\Proteus\USUARIOS\2022\Dr. David Morales\Adquisicion\105 (MVVT-5).ngb-ds3

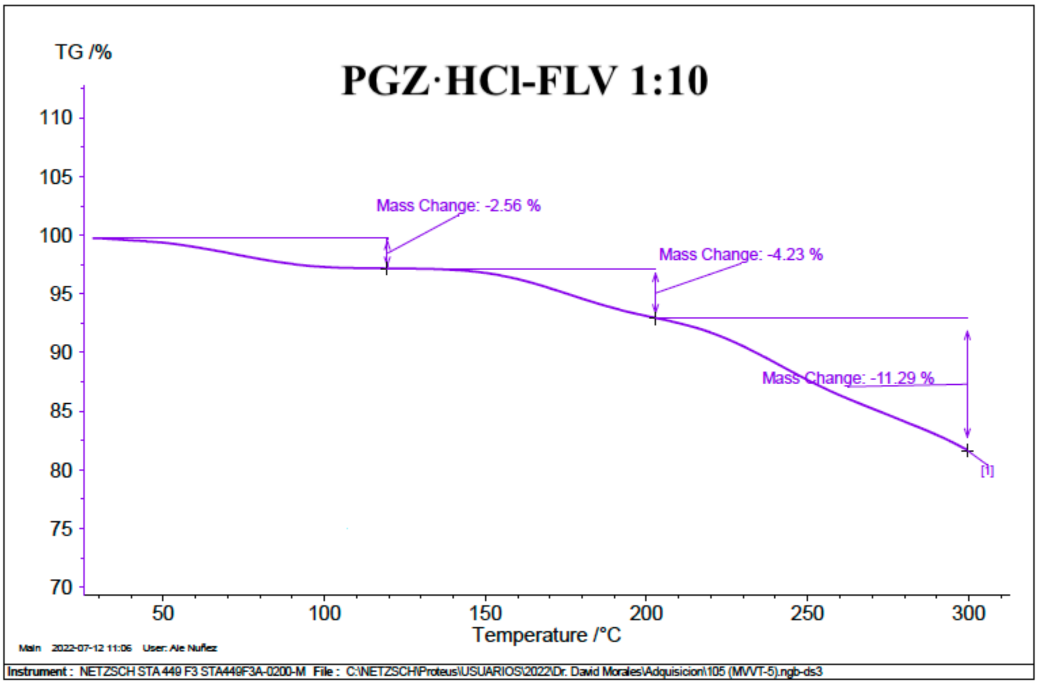
TG /%
PGZ·HCl-FLV 1:10
Mass Change: -2.56 %
Mass Change: -4.23 %
Mass Change: -11.29 %
Temperature /°C
Main 2022-07-12 11:06 User: Ale Nuñez
Instrument : NETZSCH STA 449 F3 STA449F3A-0200-M File : C:\NETZSCH\Proteus\USUARIOS\2022\Dr. David Morales\Adquisicion\105 (MVVT-5).ngb-ds3

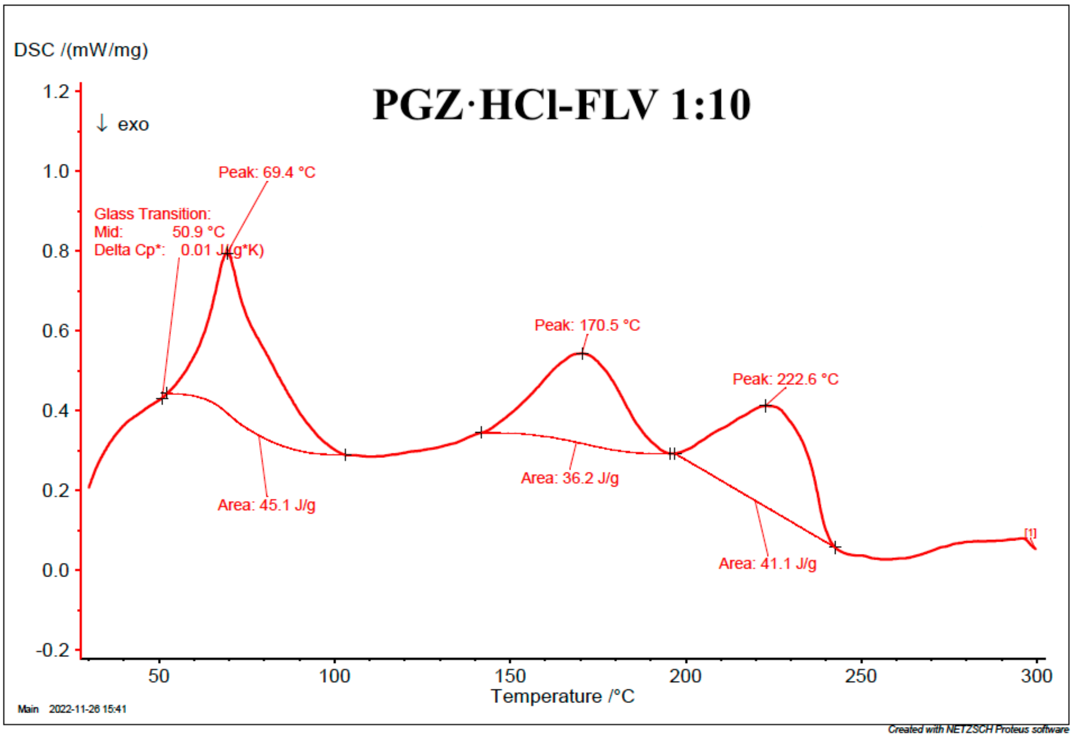

Figure SM7. Individual DSC-TGA thermograms of the solid forms of PGZ·HCl-FLV at different stoichiometric ratios (1:2; 1:4; 1:6; 1:8 and 1:10)

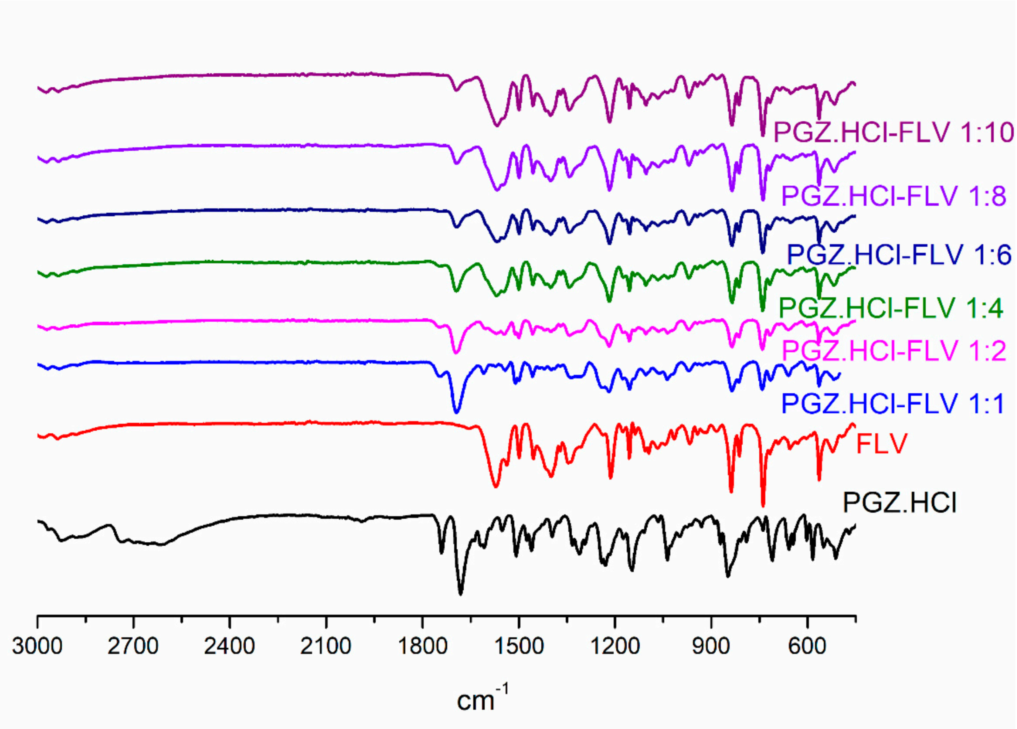

Figure SM8. Full FT-IR spectra of the solid forms of PGZ·HCl-FLV at different stoichiometric ratios (1:1; 1:2; 1:4; 1:6; 1:8 and 1:10)

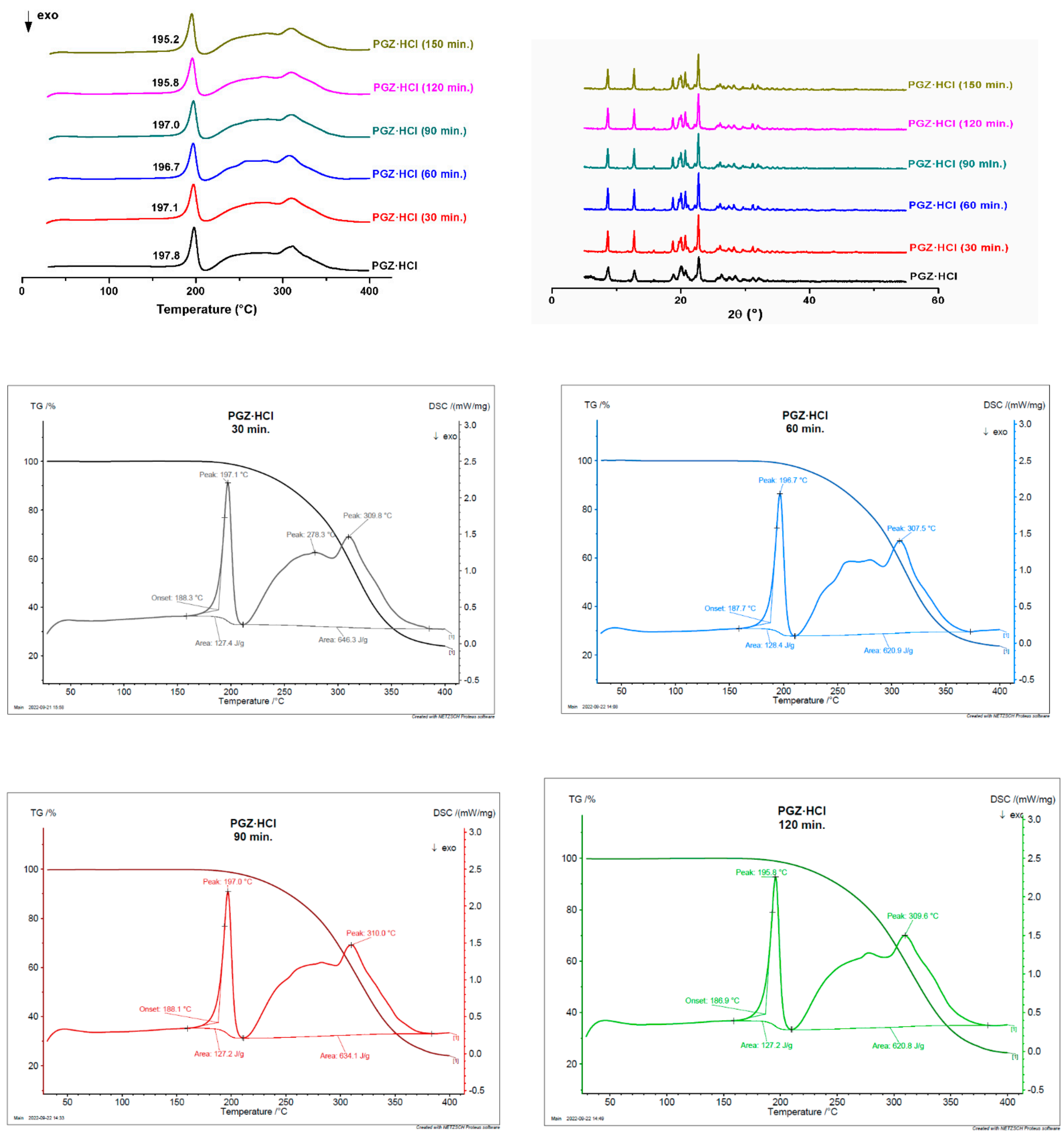
exo
195.2
195.8
197.0
196.7
197.1
197.8
PGZ·HCl (150 min.)
PGZ·HCl (120 min.)
PGZ·HCl (90 min.)
PGZ·HCl (60 min.)
PGZ·HCl (30 min.)
PGZ·HCl
Temperature (°C)
2θ (°)
TG /%
DSC /(mW/mg)
PGZ·HCl
30 min.
Peak: 197.1 °C
Peak: 309.8 °C
Peak: 278.3 °C
Onset: 188.3 °C
Area: 127.4 J/g
Area: 646.3 J/g
Temperature /°C
PGZ·HCl
60 min.
Peak: 196.7 °C
Peak: 307.5 °C
Onset: 187.7 °C
Area: 128.4 J/g
Area: 620.9 J/g
PGZ·HCl
90 min.
Peak: 197.0 °C
Peak: 310.0 °C
Onset: 188.1 °C
Area: 127.2 J/g
Area: 634.1 J/g
PGZ·HCl
120 min.
Peak: 195.8 °C
Peak: 309.6 °C
Onset: 186.9 °C
Area: 127.2 J/g
Area: 620.8 J/g

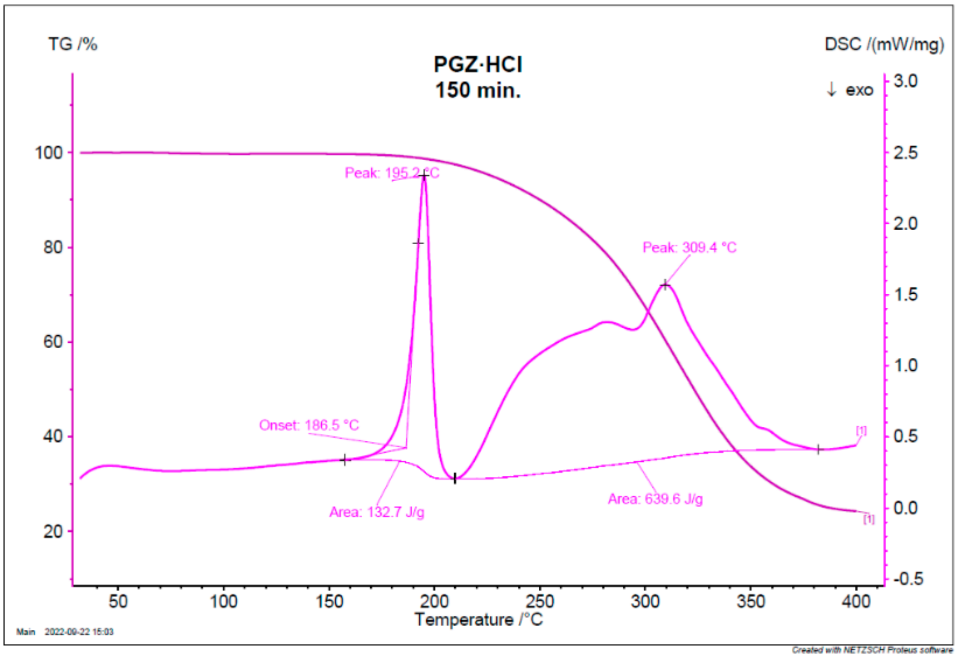

Figure SM9. Evaluation of the amorphization ability of the PGZ·HCl

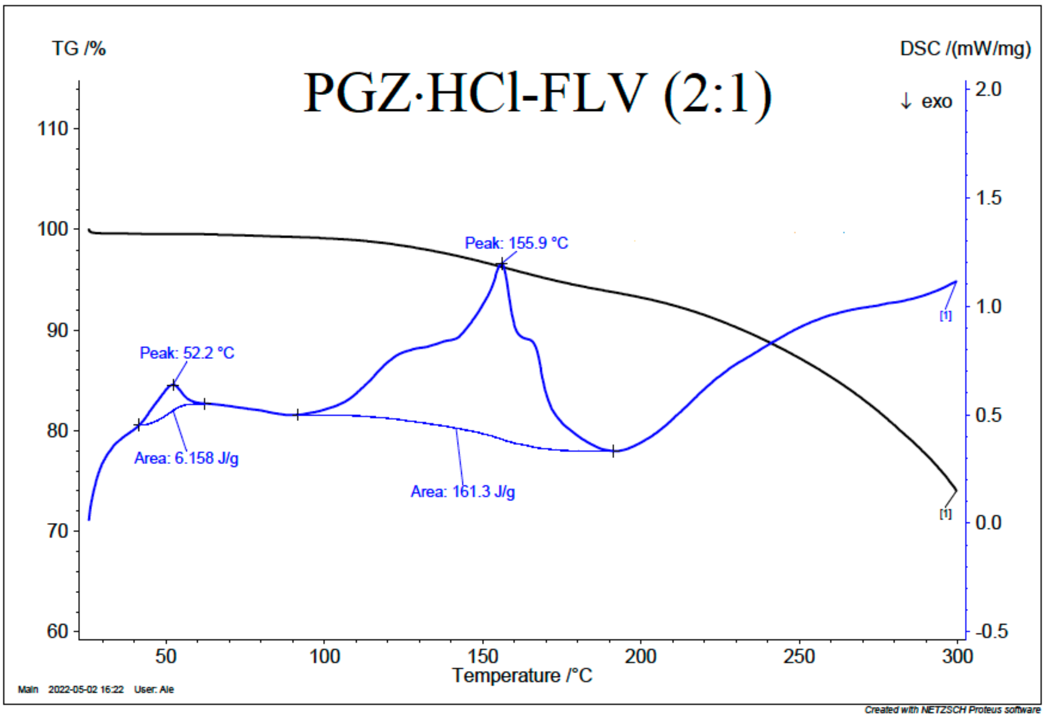

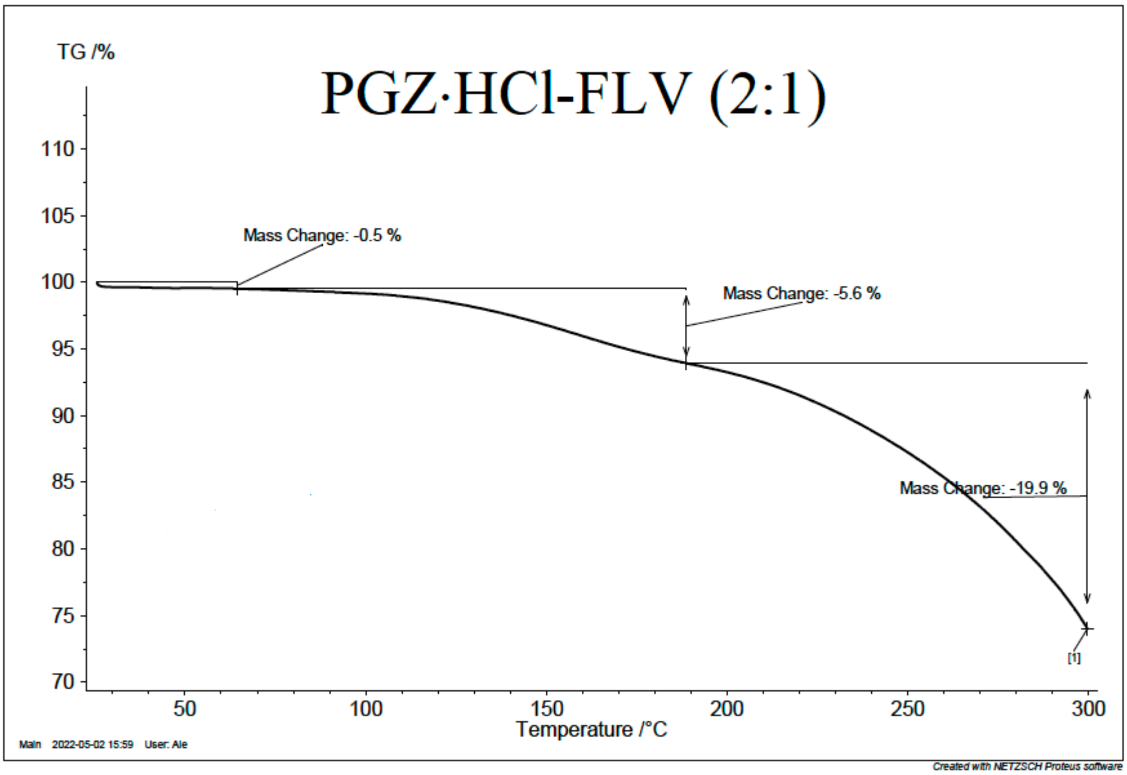
TG /%
PGZ·HCl-FLV (2:1)
110
105
100
95
90
85
80
75
70
Mass Change: -0.5 %
Mass Change: -5.6 %
Mass Change: -19.9 %
[1]
50
100
150
200
250
300
Temperature /°C
Main 2022-05-02 15:59 User: Ale
Created with NETZSCH Proteus software

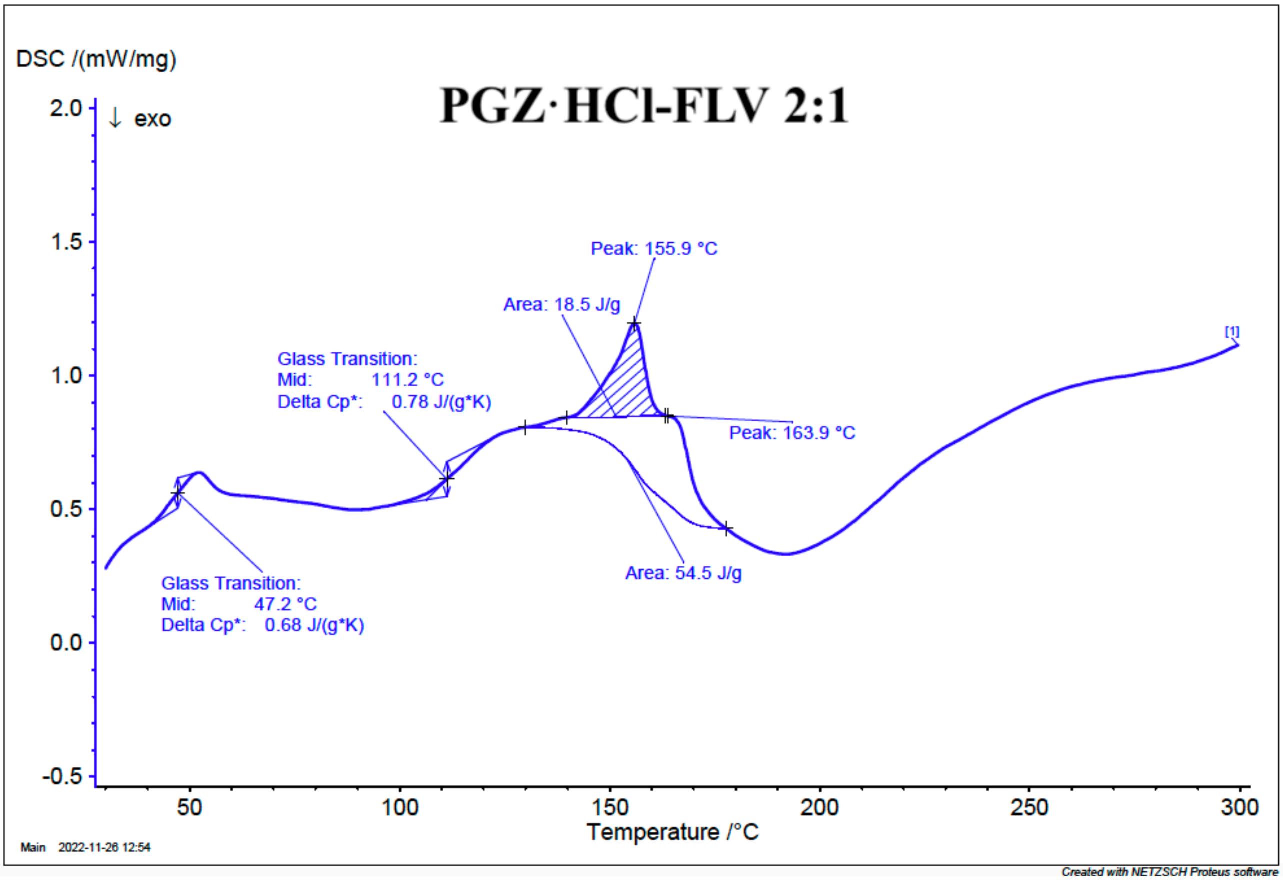
DSC /(mW/mg)
↓ exo
PGZ·HCl-FLV 2:1
2.0
1.5
1.0
0.5
0.0
-0.5
Peak: 155.9 °C
Area: 18.5 J/g
Glass Transition:
Mid: 111.2 °C
Delta Cp*: 0.78 J/(g*K)
Peak: 163.9 °C
Area: 54.5 J/g
Glass Transition:
Mid: 47.2 °C
Delta Cp*: 0.68 J/(g*K)
[1]
50
100
150
200
250
300
Temperature /°C
Main 2022-11-26 12:54
Created with NETZSCH Proteus software

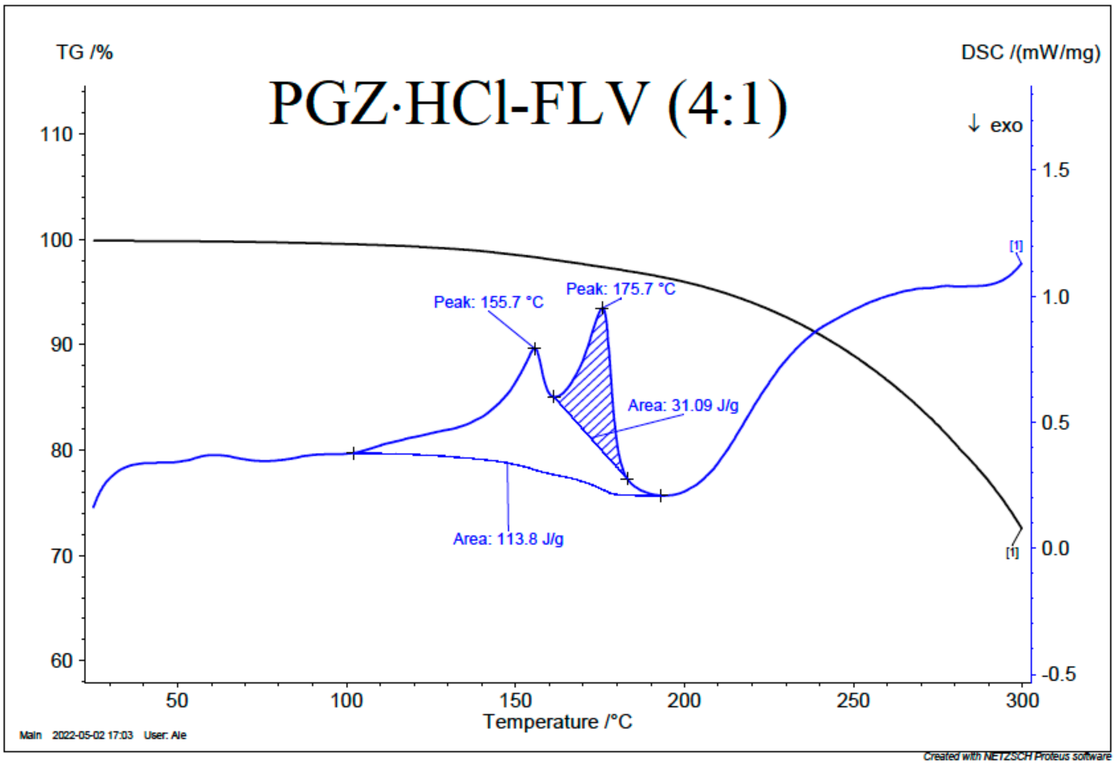
TG /%
DSC /(mW/mg)
PGZ·HCl-FLV (4:1)
↓ exo
Peak: 155.7 °C
Peak: 175.7 °C
Area: 31.09 J/g
Area: 113.8 J/g
[1]
[1]
Temperature /°C
Main 2022-05-02 17:03 User: Ale
Created with NETZSCH Proteus software

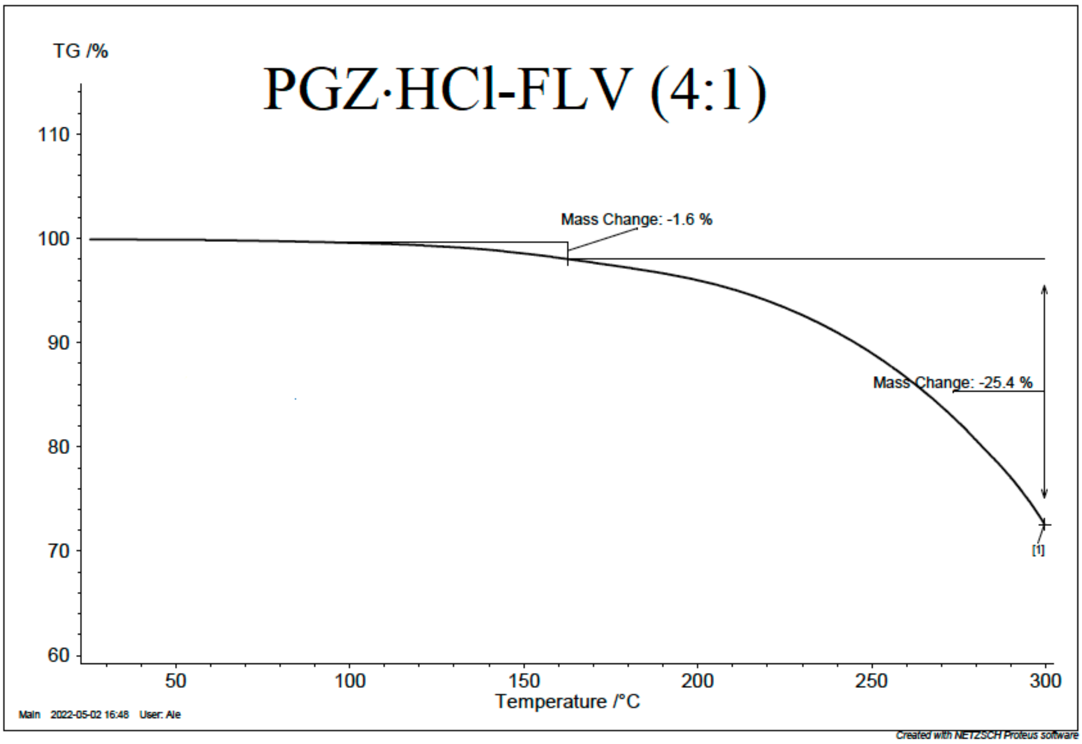
TG /%
PGZ·HCl-FLV (4:1)
Mass Change: -1.6 %
Mass Change: -25.4 %
[1]
Temperature /°C
Main 2022-05-02 16:48 User: Ale
Created with NETZSCH Proteus software

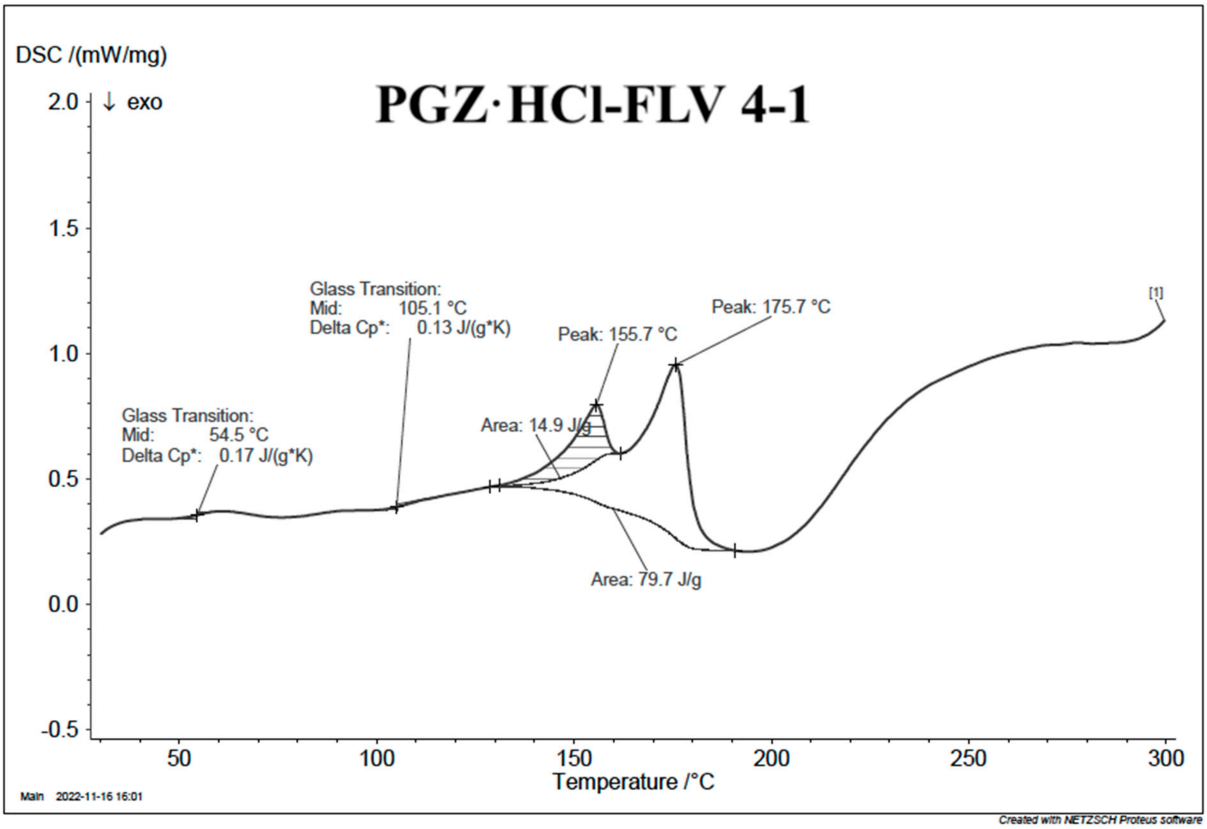
DSC /(mW/mg)
↓ exo
PGZ·HCl-FLV 4-1
Glass Transition:
Mid: 105.1 °C
Delta Cp*: 0.13 J/(g*K)
Peak: 155.7 °C
Peak: 175.7 °C
Glass Transition:
Mid: 54.5 °C
Delta Cp*: 0.17 J/(g*K)
Area: 14.9 J/g
Area: 79.7 J/g
[1]
Temperature /°C
Main 2022-11-16 16:01
Created with NETZSCH Proteus software

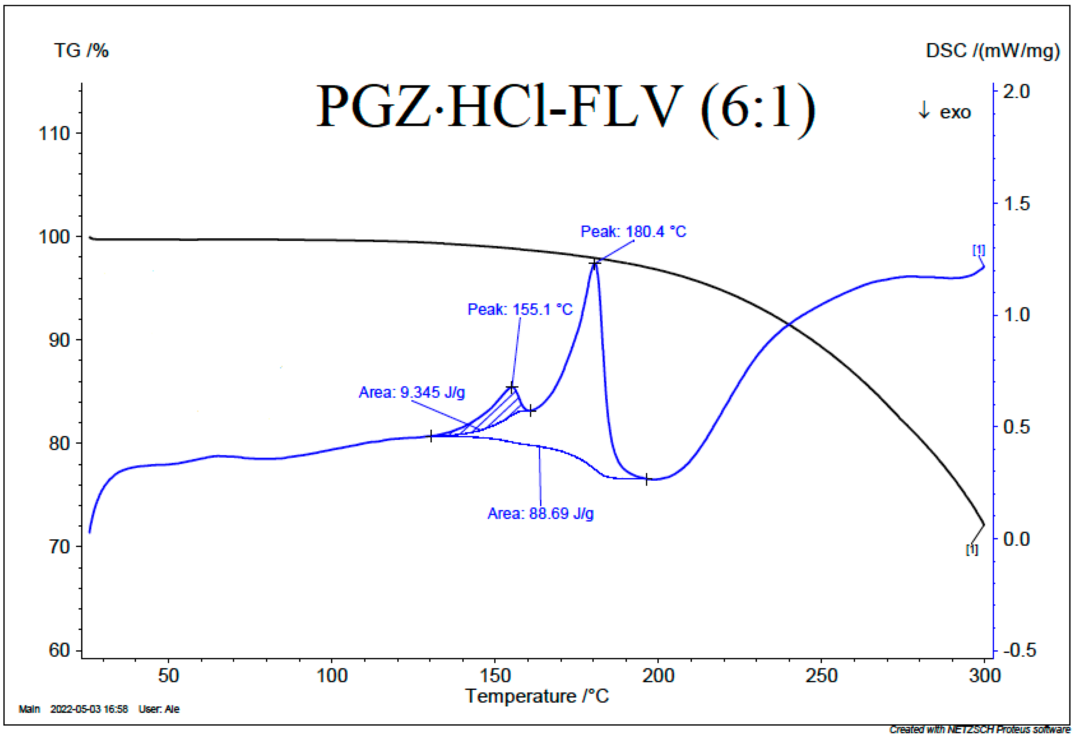
TG /%
DSC /(mW/mg)
PGZ·HCl-FLV (6:1)
↓ exo
Peak: 180.4 °C
Peak: 155.1 °C
Area: 9.345 J/g
Area: 88.69 J/g
[1]
[1]
Temperature /°C
Main 2022-05-03 16:58 User: Ale
Created with NETZSCH Proteus software

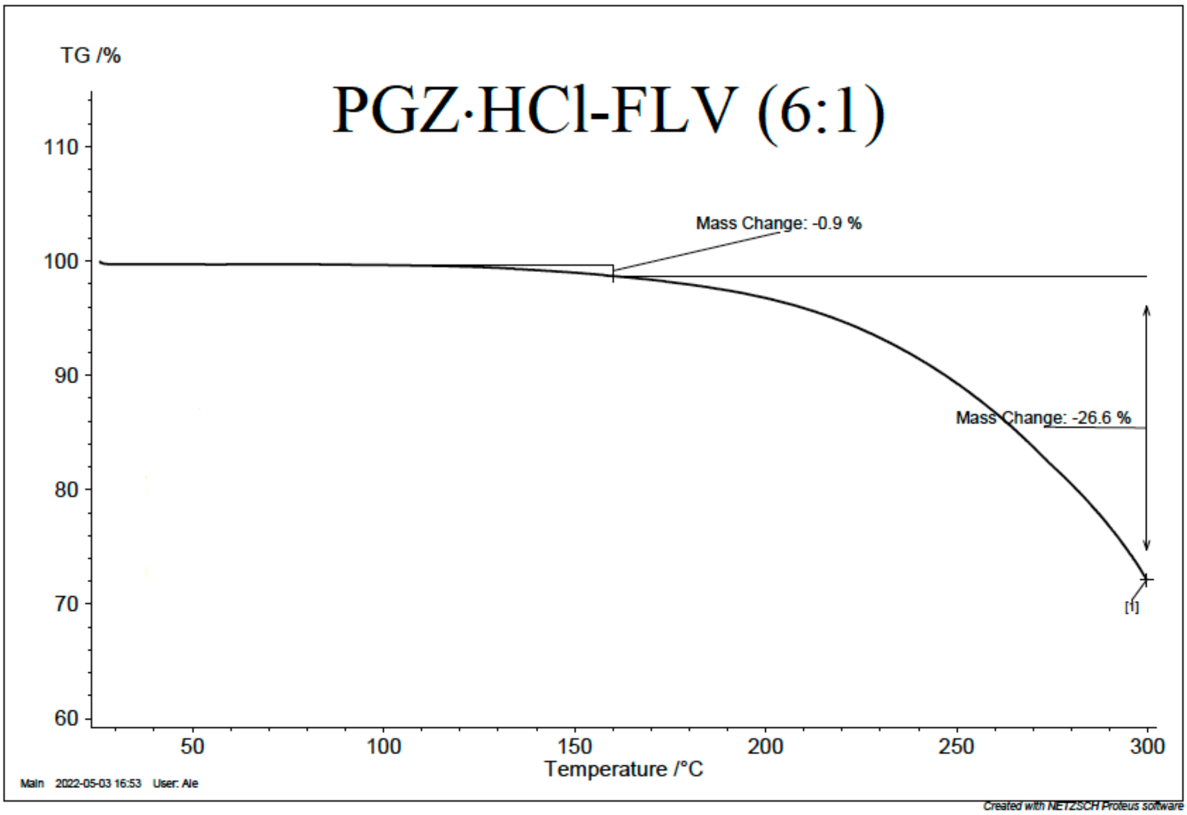

TG /%
PGZ·HCl-FLV (6:1)
Mass Change: -0.9 %
Mass Change: -26.6 %
[1]
110
100
90
80
70
60
50
100
150
200
250
300
Temperature /°C
Main 2022-05-03 16:53 User: Ale
Created with NETZSCH Proteus software

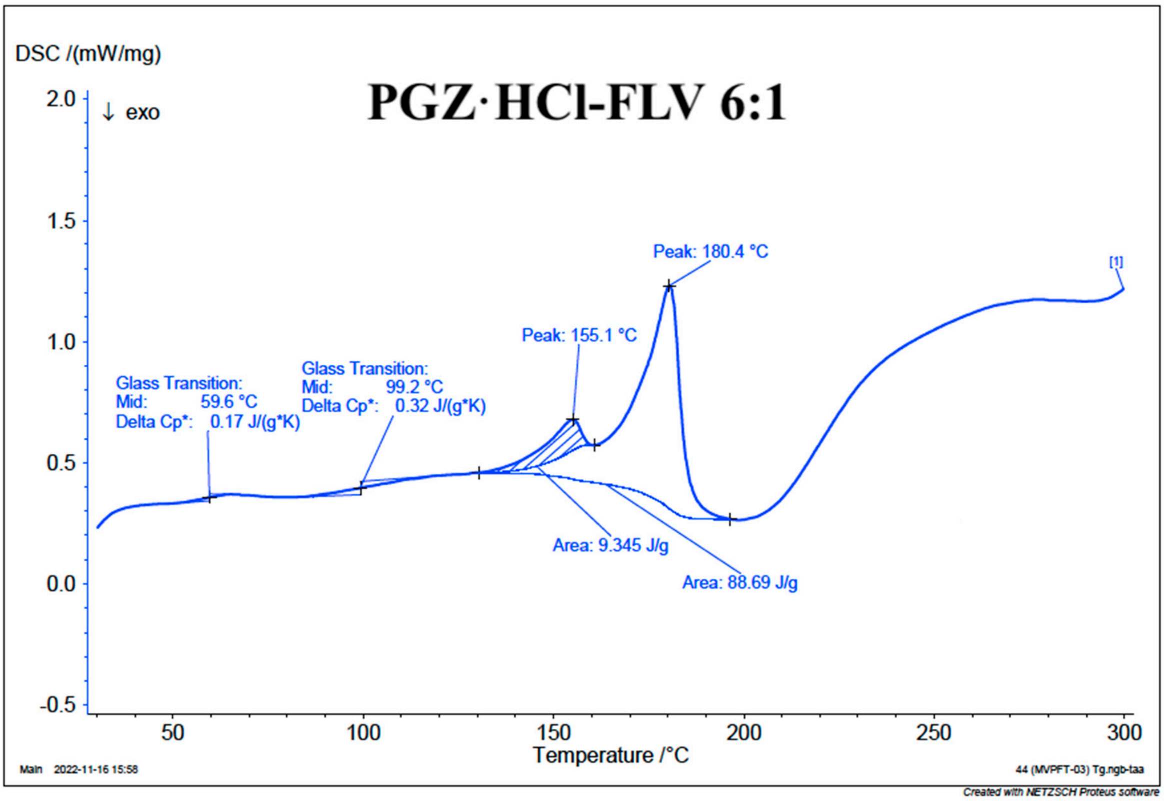

DSC /(mW/mg)
↓ exo
PGZ·HCl-FLV 6:1
Peak: 180.4 °C
Peak: 155.1 °C
[1]
Glass Transition:
Mid: 59.6 °C
Delta Cp*: 0.17 J/(g*K)
Glass Transition:
Mid: 99.2 °C
Delta Cp*: 0.32 J/(g*K)
Area: 9.345 J/g
Area: 88.69 J/g
2.0
1.5
1.0
0.5
0.0
-0.5
50
100
150
200
250
300
Temperature /°C
Main 2022-11-16 15:58
44 (MVPFT-03) Tg.ngb-taa
Created with NETZSCH Proteus software

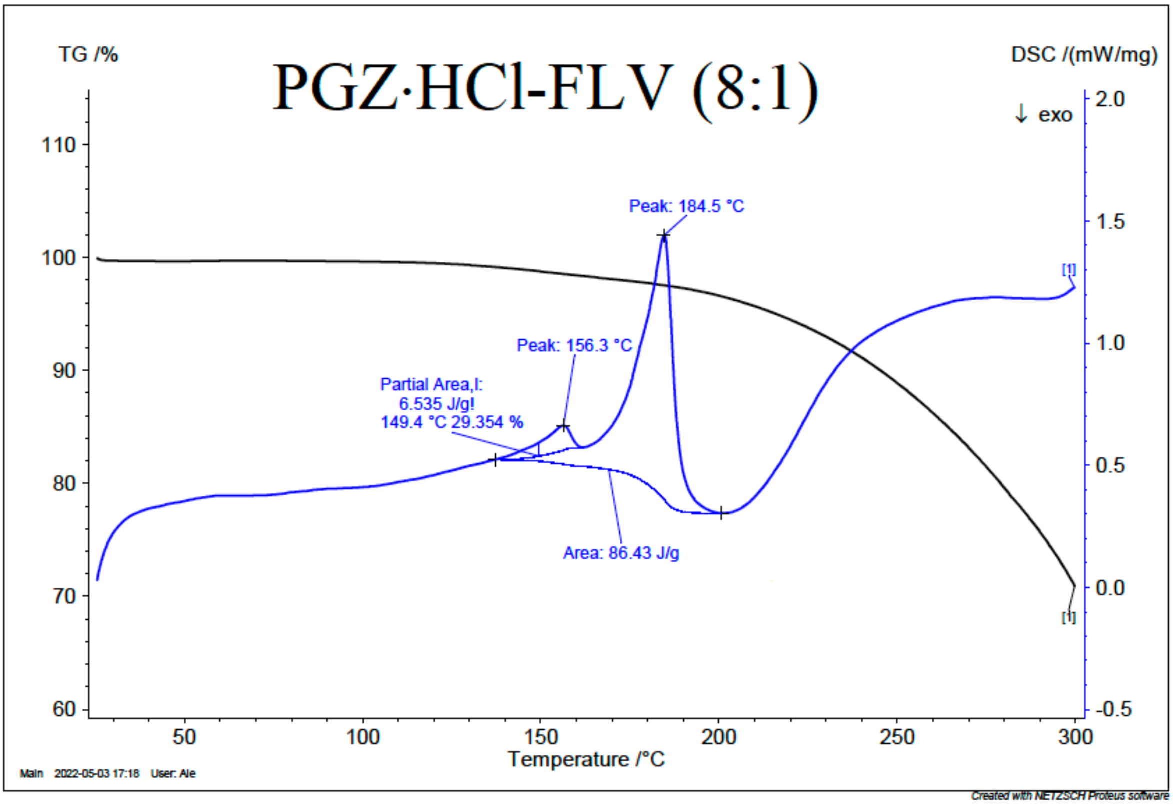
PGZ·HCl-FLV (8:1)
TG /%
DSC /(mW/mg)
↓ exo
Peak: 184.5 °C
Peak: 156.3 °C
Partial Area,I:
6.535 J/g!
149.4 °C 29.354 %
Area: 86.43 J/g
[1]
Temperature /°C
Main 2022-05-03 17:18 User: Ale
Created with NETZSCH Proteus software

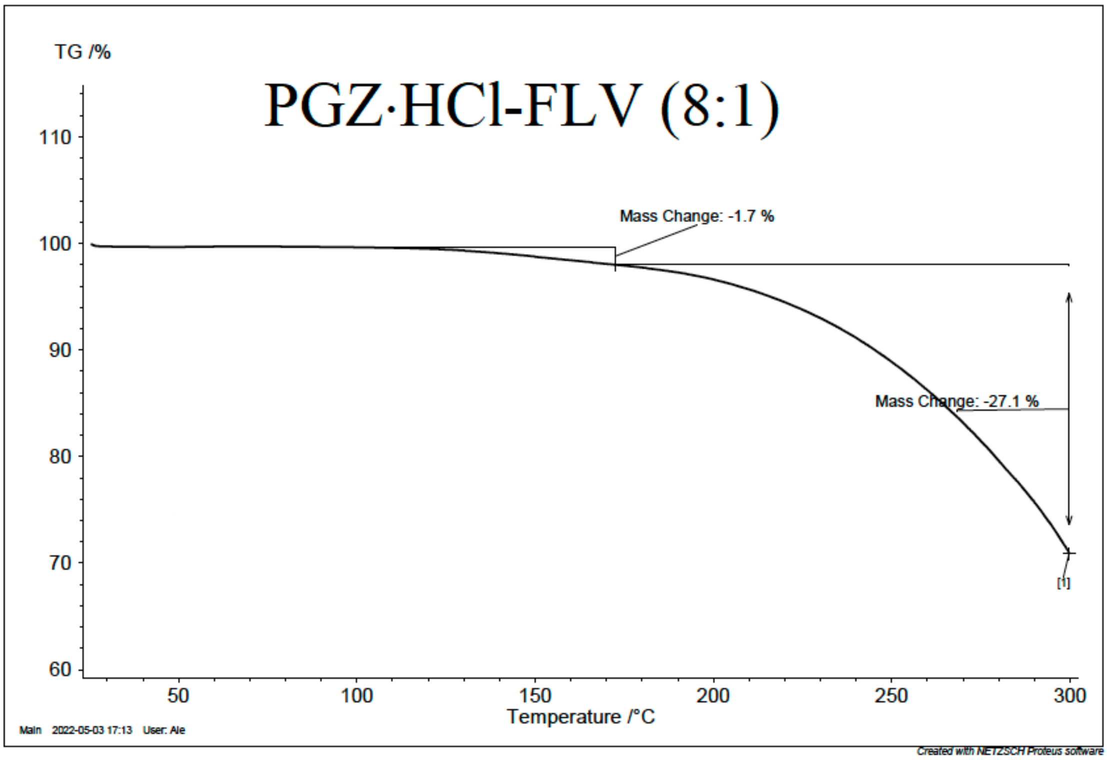
PGZ·HCl-FLV (8:1)
TG /%
Mass Change: -1.7 %
Mass Change: -27.1 %
[1]
Temperature /°C
Main 2022-05-03 17:13 User: Ale
Created with NETZSCH Proteus software

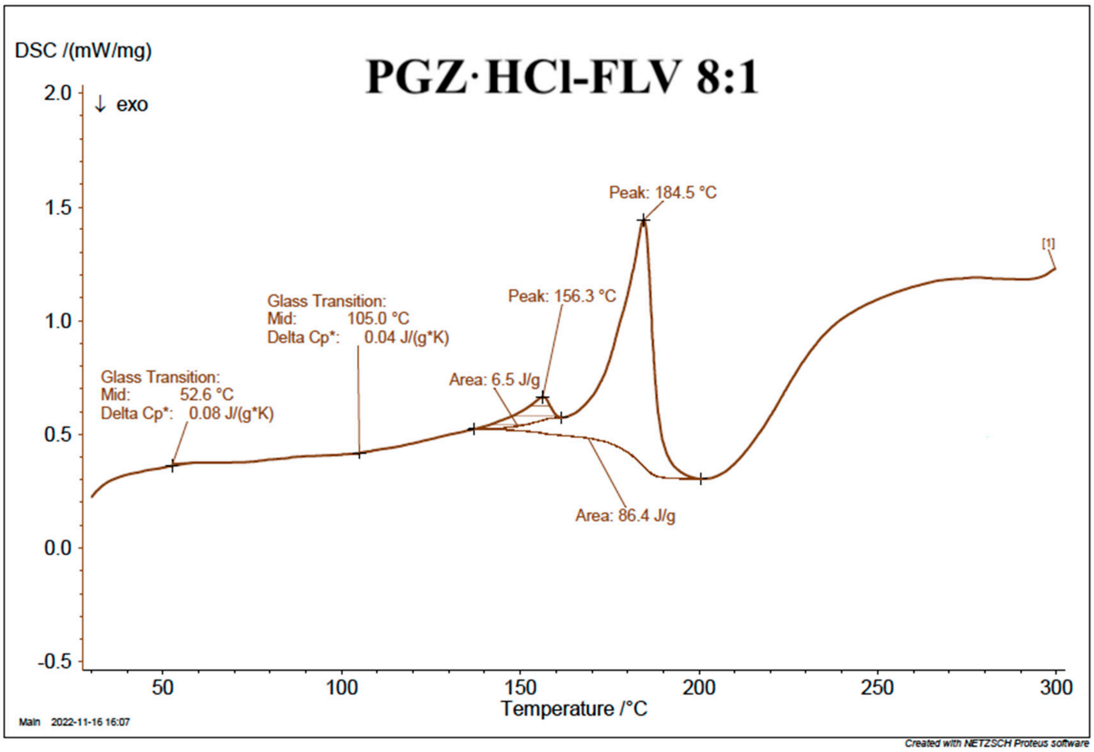

PGZ·HCl-FLV 8:1
DSC /(mW/mg)
↓ exo
Peak: 184.5 °C
Peak: 156.3 °C
Glass Transition:
Mid: 105.0 °C
Delta Cp*: 0.04 J/(g*K)
Glass Transition:
Mid: 52.6 °C
Delta Cp*: 0.08 J/(g*K)
Area: 6.5 J/g
Area: 86.4 J/g
[1]
2.0
1.5
1.0
0.5
0.0
-0.5
50
100
150
200
250
300
Temperature /°C
Main 2022-11-16 16:07
Created with NETZSCH Proteus software

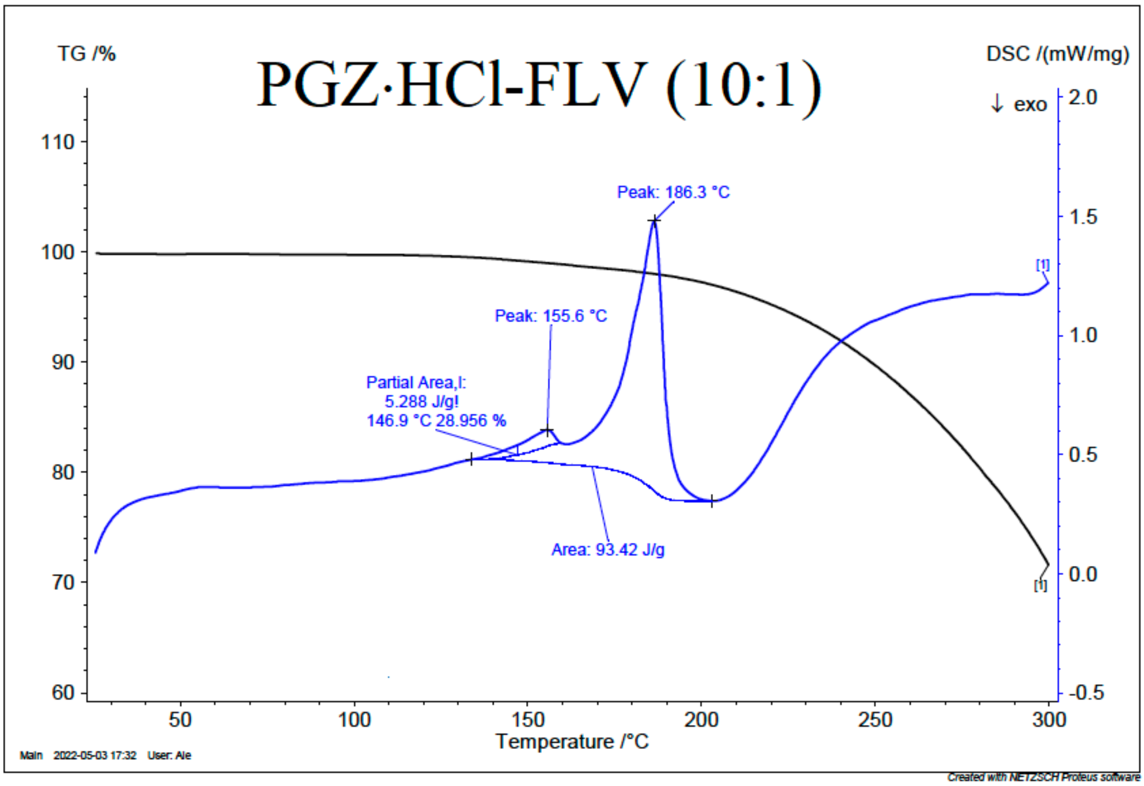

PGZ·HCl-FLV (10:1)
TG /%
DSC /(mW/mg)
↓ exo
Peak: 186.3 °C
Peak: 155.6 °C
Partial Area,I:
5.288 J/g!
146.9 °C 28.956 %
Area: 93.42 J/g
[1]
[1]
110
100
90
80
70
60
2.0
1.5
1.0
0.5
0.0
-0.5
50
100
150
200
250
300
Temperature /°C
Main 2022-05-03 17:32 User: Ale
Created with NETZSCH Proteus software

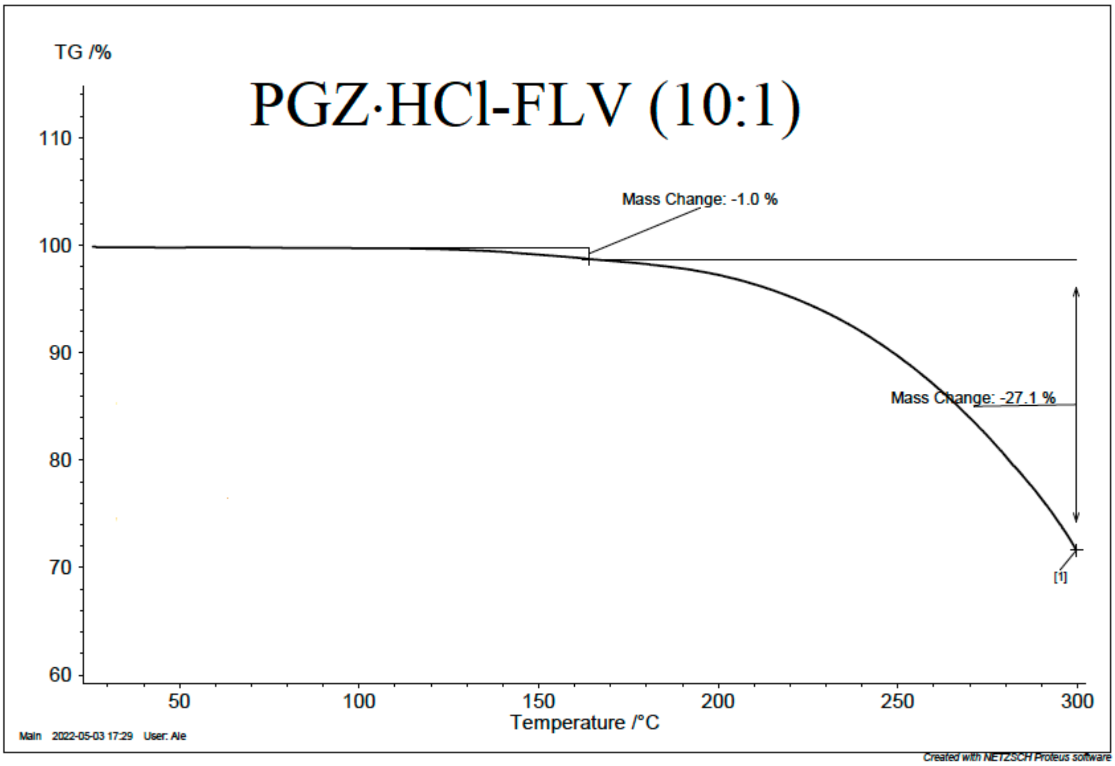

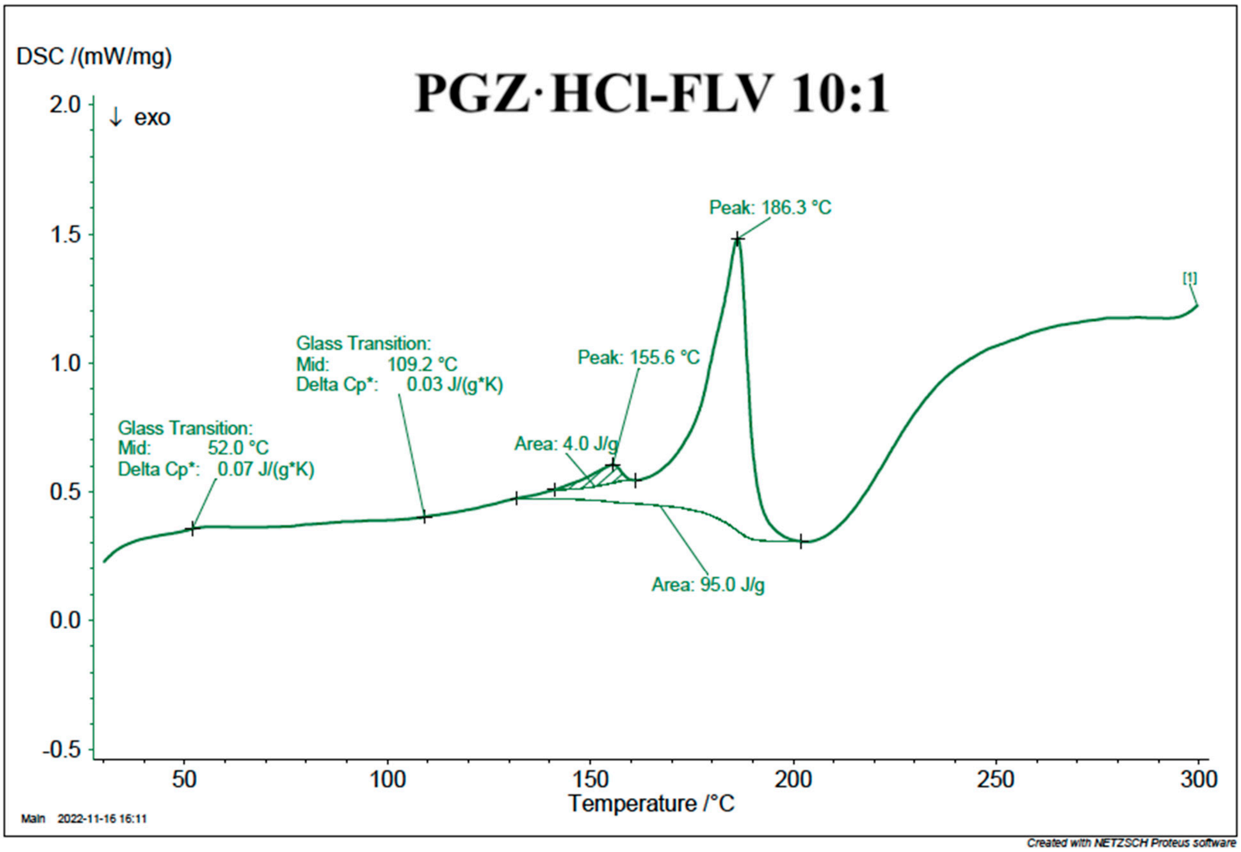

Figure SM10. Individual DSC-TGA thermograms of the solid forms of PGZ·HCl-FLV at different stoichiometric ratios (2:1; 4:1; 6:1; 8:1 and 10:1)

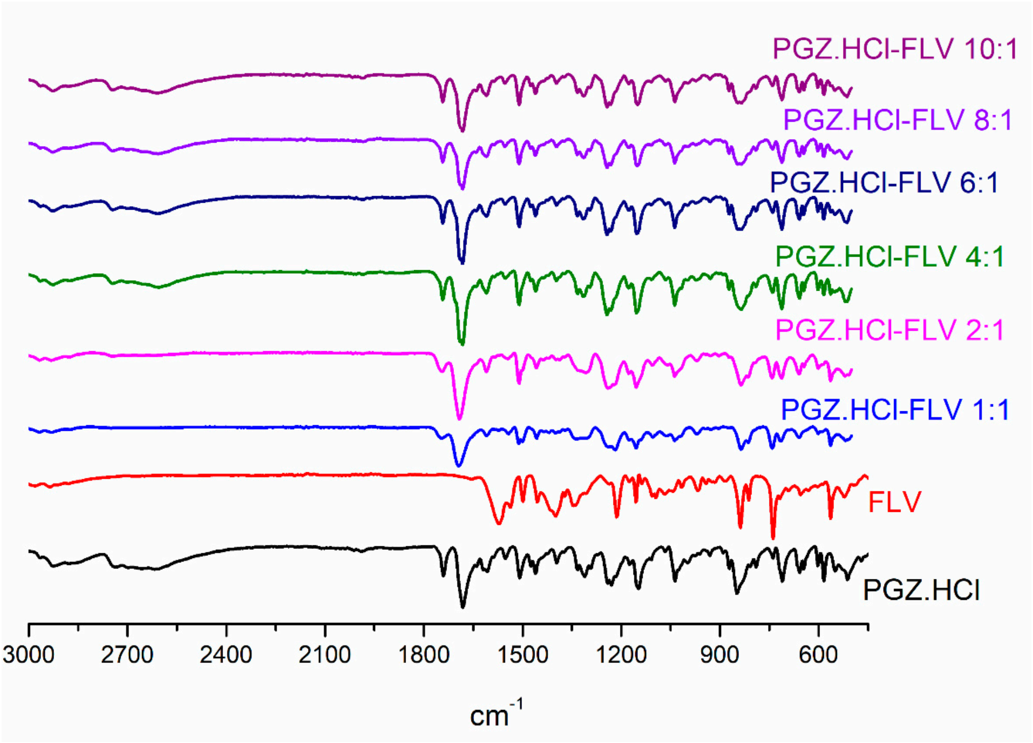

Figure SM11. Full FT-IR spectra of the solid forms of PGZ·HCl-FLV at different stoichiometric ratios (2:1; 4:1; 6:1; 8:1 and 10:1)

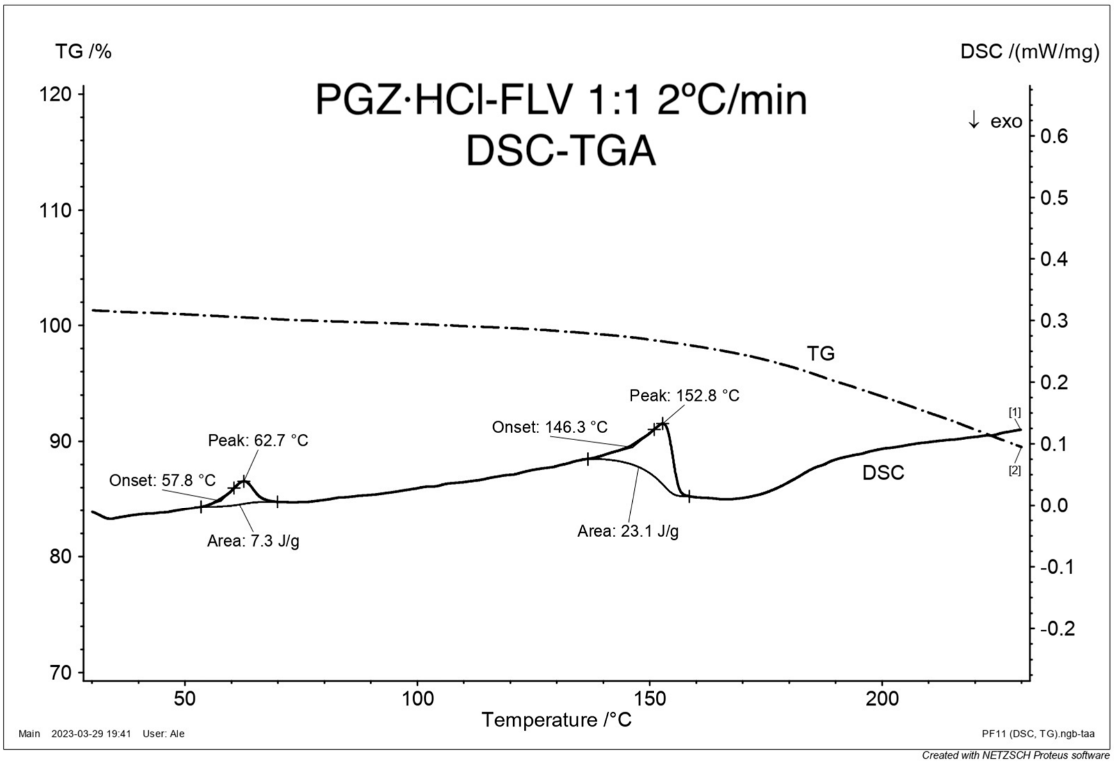

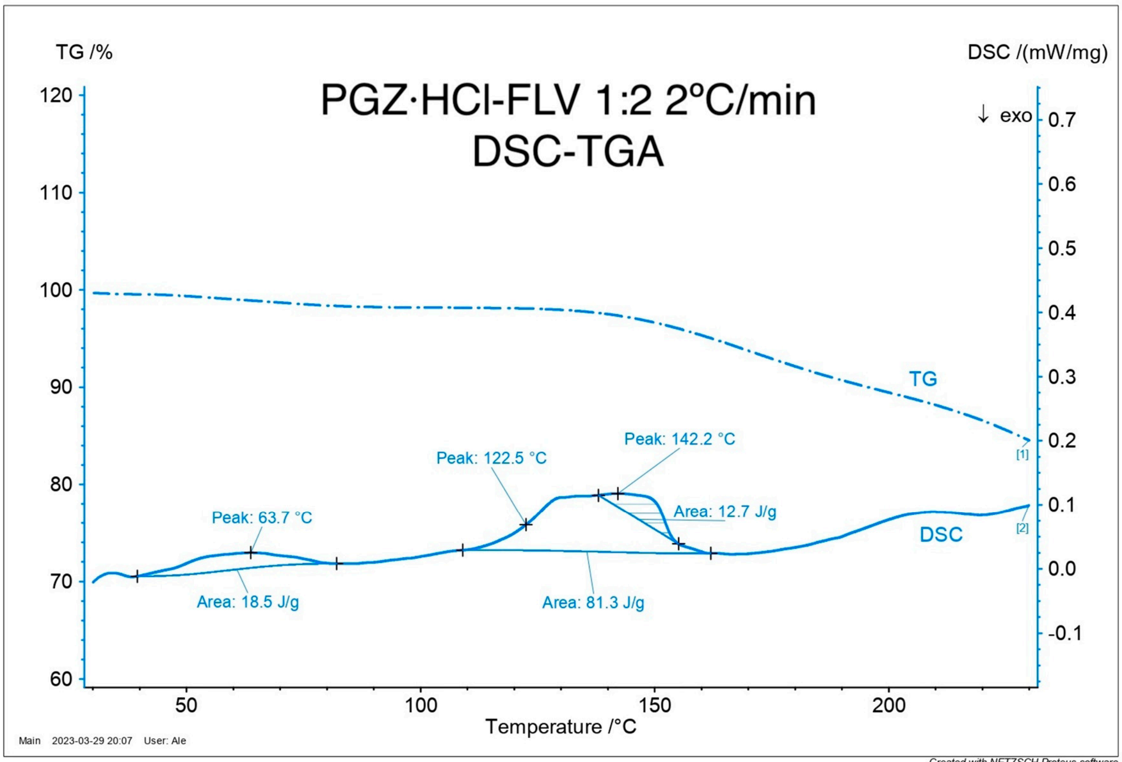

Created with NETZSCH Proteus software

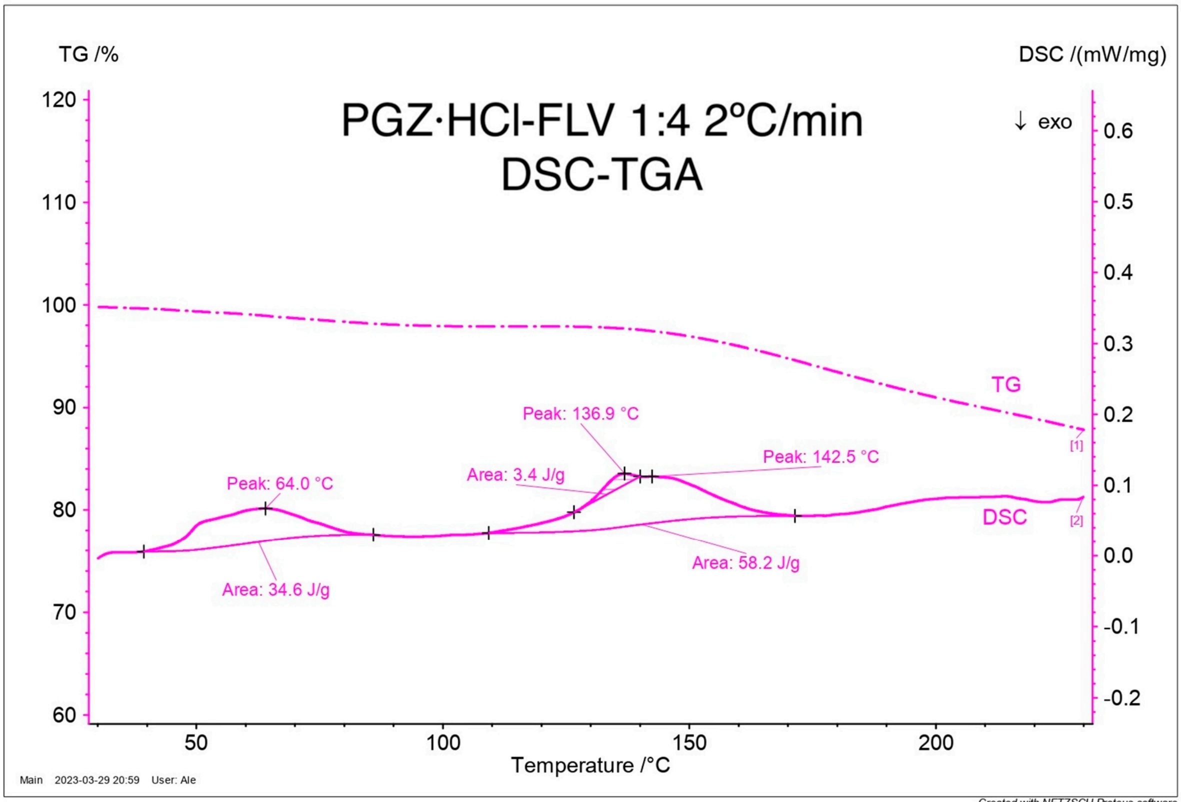

Created with NETZSCH Proteus software

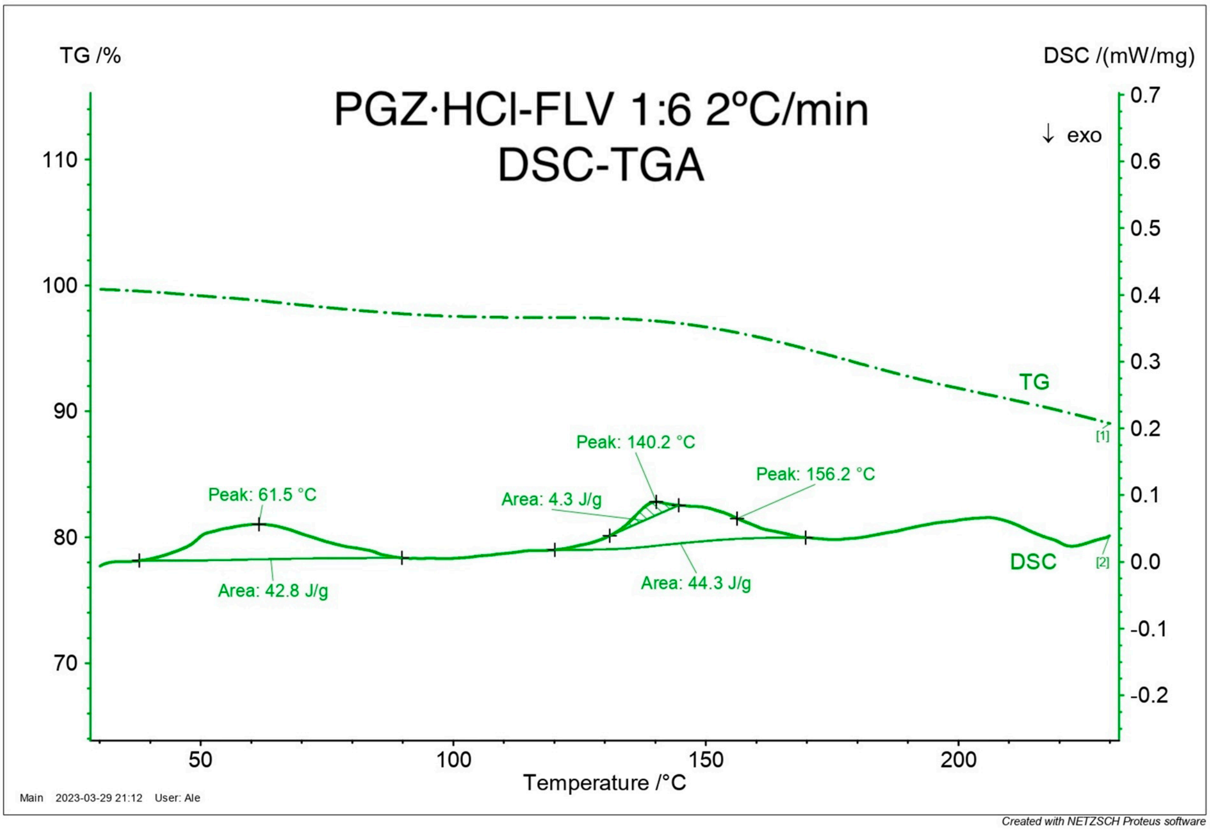
TG /%
DSC /(mW/mg)
PGZ·HCl-FLV 1:6 2ºC/min
DSC-TGA
↓ exo
TG
DSC
Peak: 61.5 °C
Area: 42.8 J/g
Peak: 140.2 °C
Area: 4.3 J/g
Peak: 156.2 °C
Area: 44.3 J/g
Temperature /°C
Main 2023-03-29 21:12 User: Ale
Created with NETZSCH Proteus software

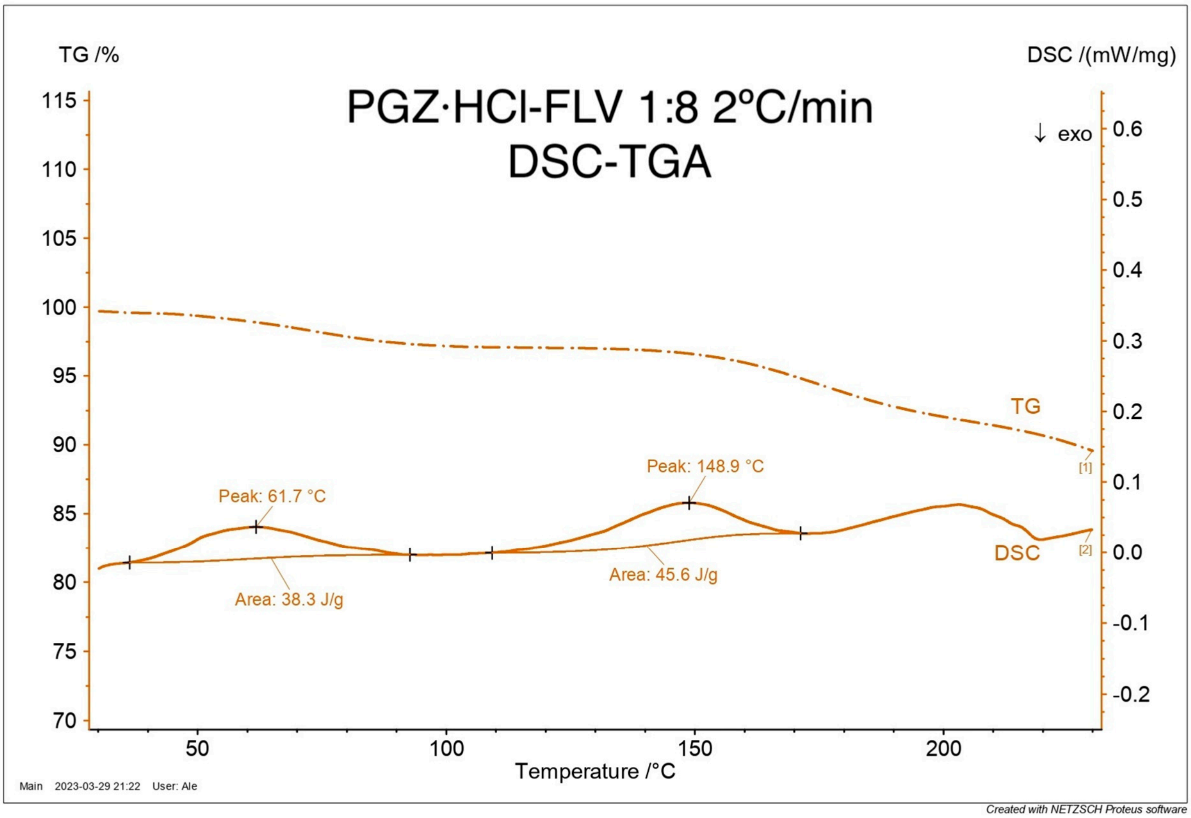
TG /%
DSC /(mW/mg)
PGZ·HCl-FLV 1:8 2ºC/min
DSC-TGA
↓ exo
TG
DSC
Peak: 61.7 °C
Area: 38.3 J/g
Peak: 148.9 °C
Area: 45.6 J/g
Temperature /°C
Main 2023-03-29 21:22 User: Ale
Created with NETZSCH Proteus software

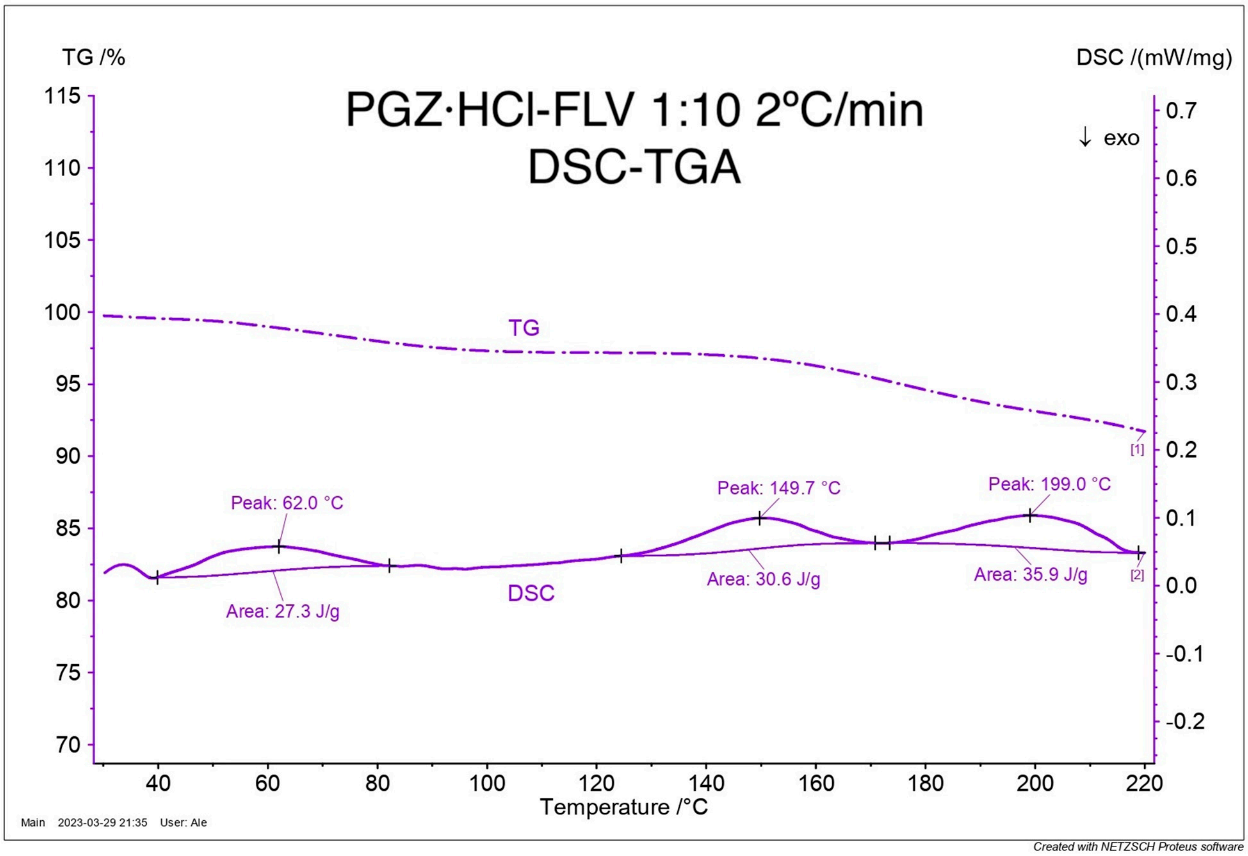
PGZ·HCl-FLV 1:10 2°C/min
DSC-TGA
TG /%
DSC /(mW/mg)
↓ exo
TG
DSC
Peak: 62.0 °C
Area: 27.3 J/g
Peak: 149.7 °C
Area: 30.6 J/g
Peak: 199.0 °C
Area: 35.9 J/g
Temperature /°C
Main 2023-03-29 21:35 User: Ale
Created with NETZSCH Proteus software

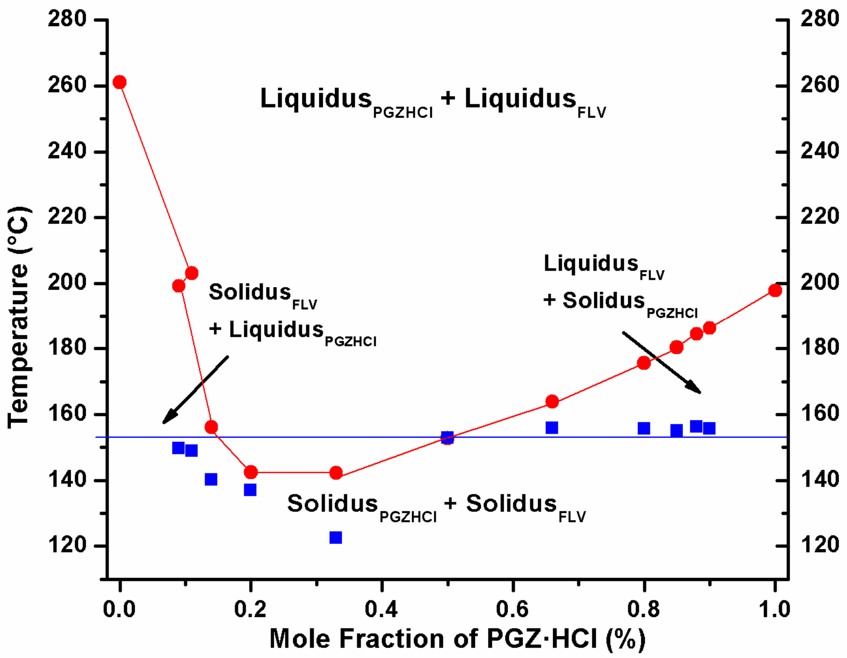
Liquidus$_{PGZHCl}$ + Liquidus$_{FLV}$
Solidus$_{FLV}$ + Liquidus$_{PGZHCl}$
Liquidus$_{FLV}$ + Solidus$_{PGZHCl}$
Solidus$_{PGZHCl}$ + Solidus$_{FLV}$
Temperature (°C)
Mole Fraction of PGZ·HCl (%)

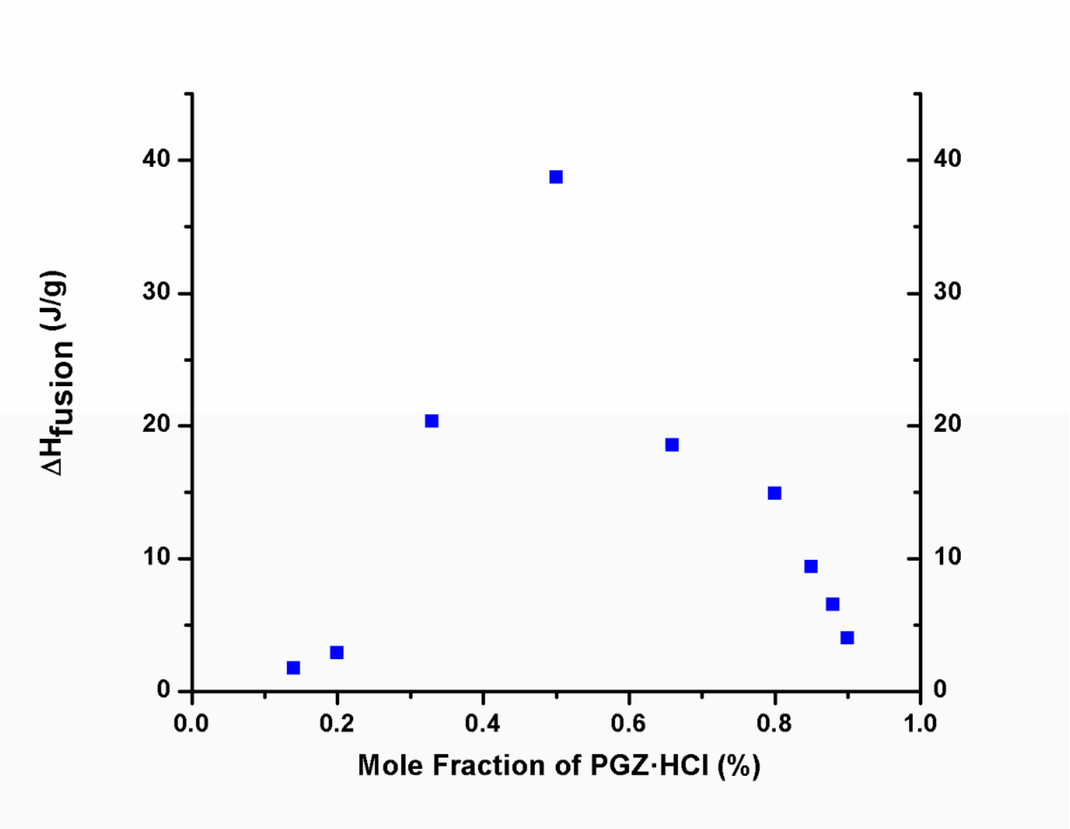

Figure SM12. PGZ·HCl-FLV binary phase diagram and Tammann's plot triangle. Thermograms corresponding to the molar ratios 1:1; 1:2; 1:4; 1:6; 1: and 1:10 at 2 °C/min.

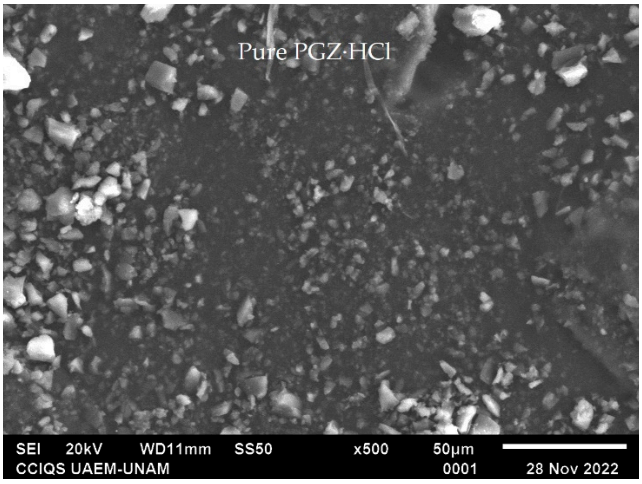

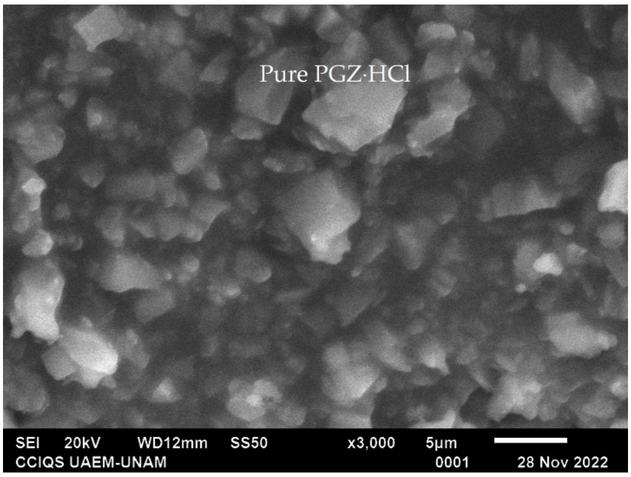

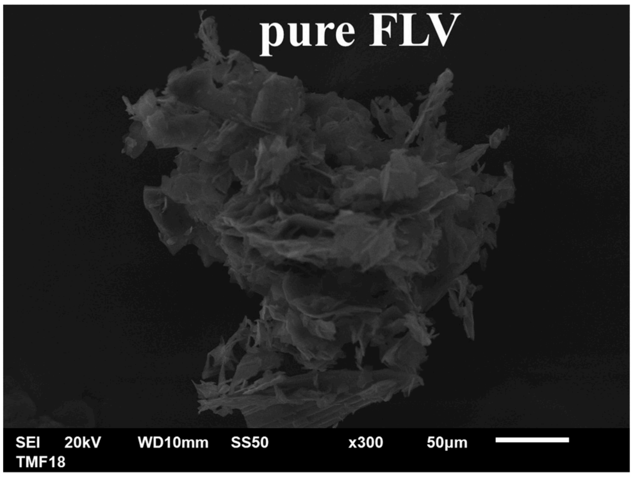
pure FLV
SEI 20kV WD10mm SS50 x300 50μm
TMF18

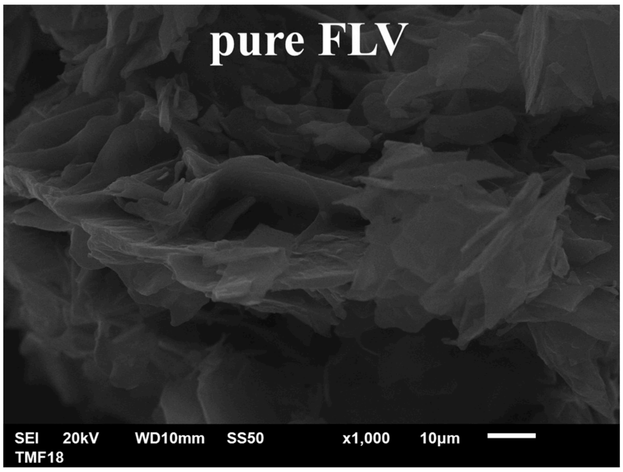
pure FLV
SEI 20kV WD10mm SS50 x1,000 10μm
TMF18

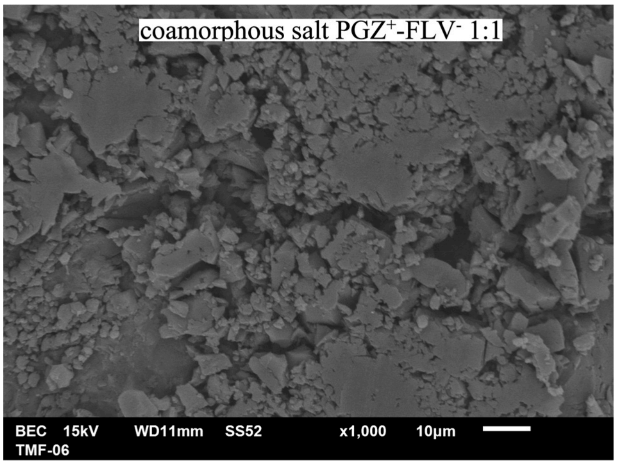
coamorphous salt PGZ^{+}-FLV^{-} 1:1
BEC 15kV WD11mm SS52 x1,000 10μm
TMF-06

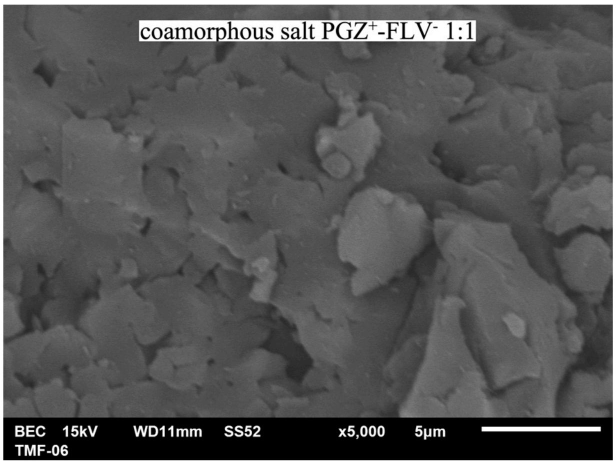
coamorphous salt PGZ^{+}-FLV^{-} 1:1
BEC 15kV WD11mm SS52 x5,000 5μm
TMF-06

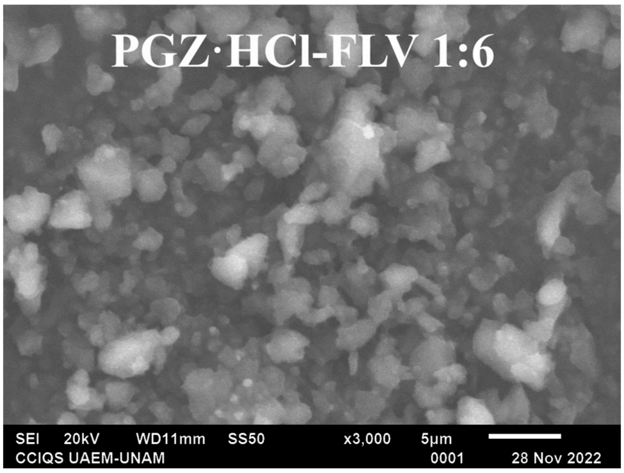
PGZ·HCl-FLV 1:6
SEI 20kV WD11mm SS50 x3,000 5μm
CCIQS UAEM-UNAM 0001 28 Nov 2022

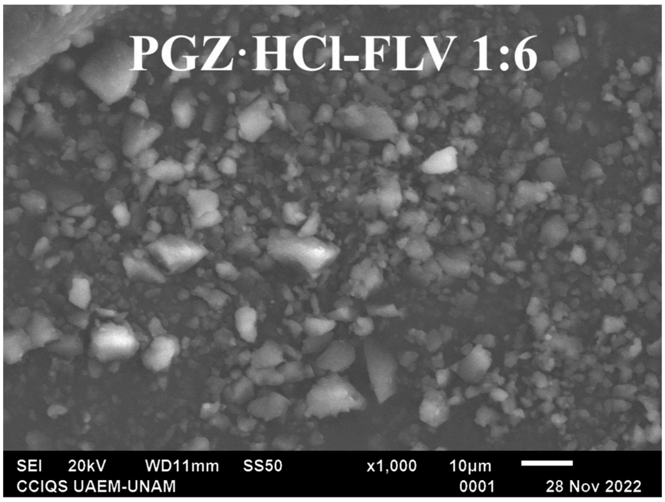
PGZ·HCl-FLV 1:6
SEI 20kV WD11mm SS50 x1,000 10μm
CCIQS UAEM-UNAM 0001 28 Nov 2022

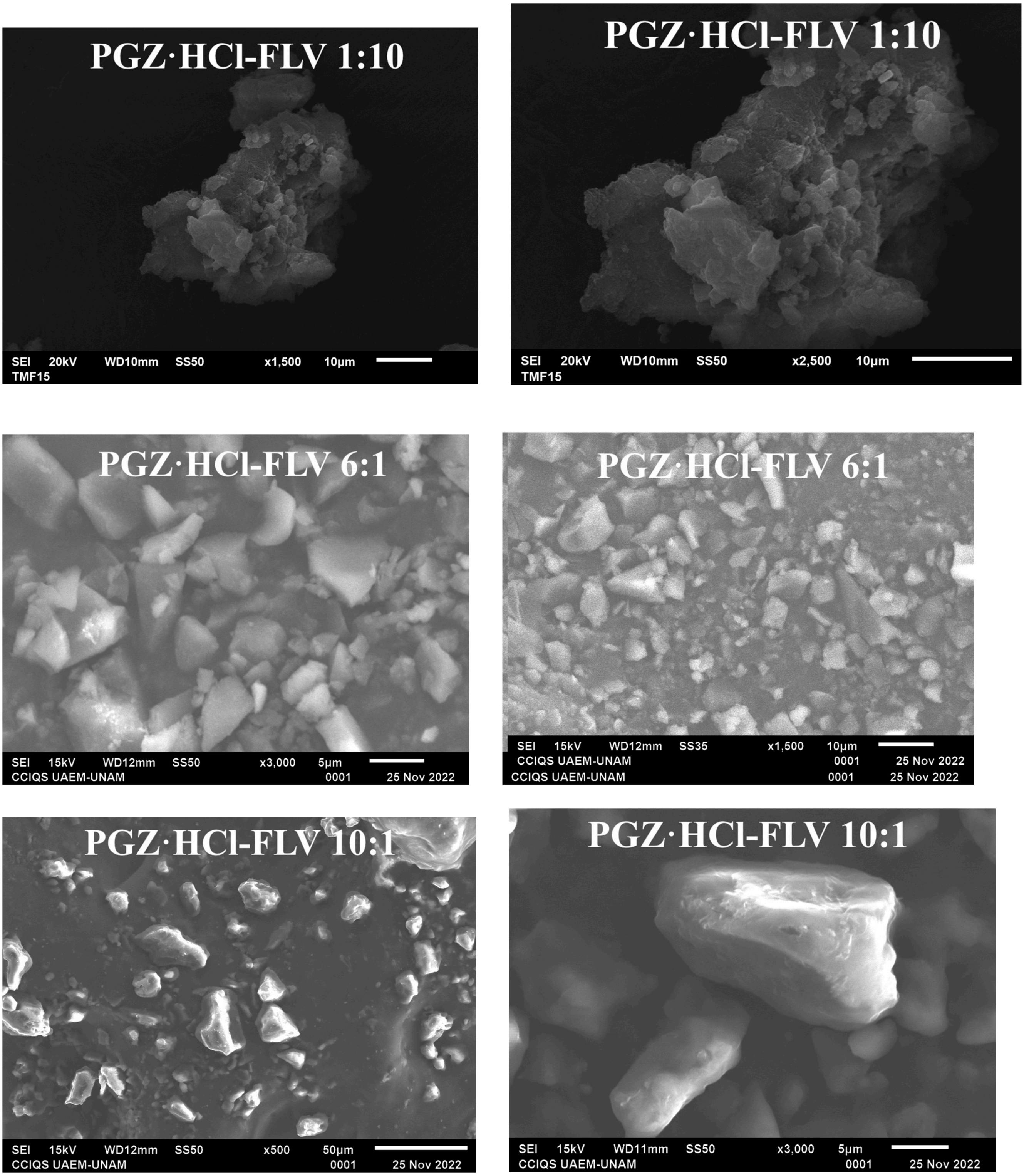

Figure SM13. Grain morphology images of the solid forms (1:1; 1:6; 1:10; 6:1; and 10:1)

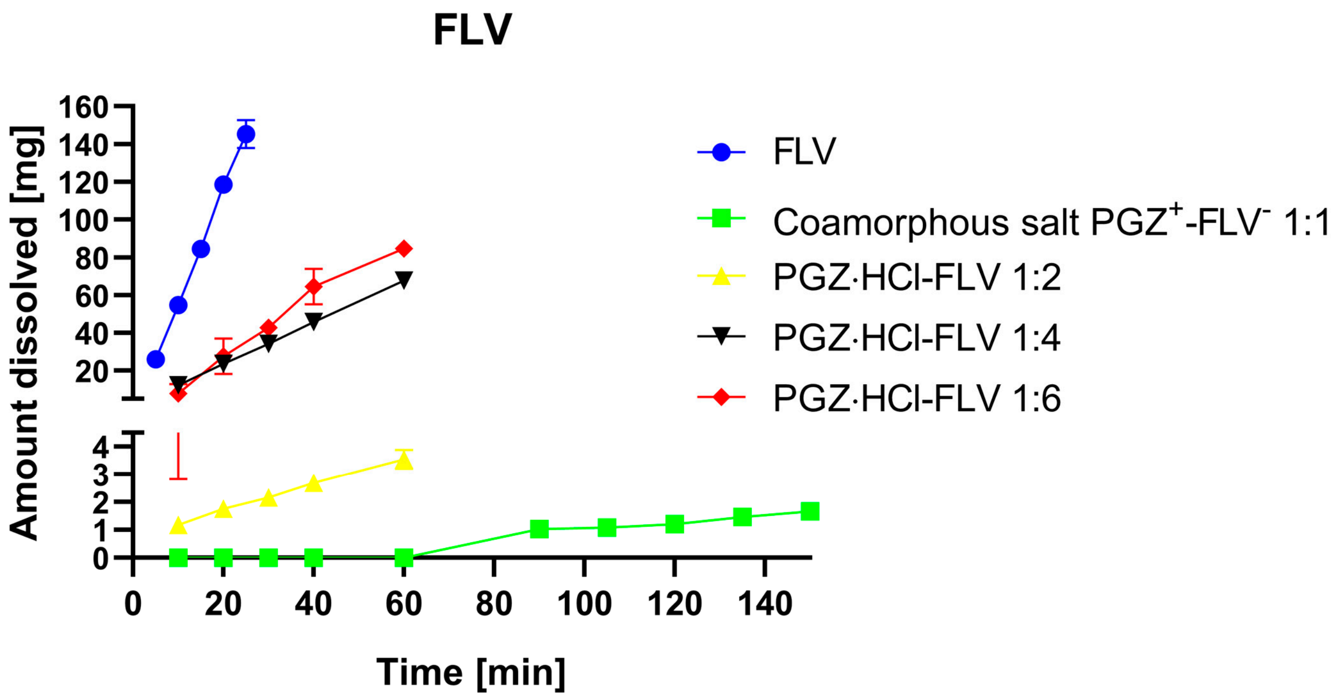

Figure SM14. Dissolution profiles with another scale on the y-axis show the plots in more detail.
